# Comparative Effects of Emerging Lp(a)‐Lowering Agents and PCSK9‐Directed Therapies on Lipoprotein(a): A Network Meta‐Analysis of Randomised Clinical Trials

**DOI:** 10.1111/dom.70944

**Published:** 2026-06-04

**Authors:** Jihad Abu Zayed, Nada A. Al‐Awamleh, Mahmoud Hamad, Mohammad Ibrahim Mahmoud Hdaib, Hazem Ayesh

**Affiliations:** ^1^ School of Medicine University of Jordan Amman Jordan; ^2^ Deaconess Health System Evansville Indiana USA

**Keywords:** cardiovascular disease, drug development, dyslipidaemia, experimental pharmacology, network meta‐analysis

## Abstract

**Aims:**

Elevated lipoprotein(a) [Lp(a)] is a genetic ASCVD risk factor that often persists despite intensive LDL‐C lowering. We compared the efficacy and safety of emerging Lp(a)‐targeted therapies (siRNAs, antisense oligonucleotides and an oral assembly inhibitor) with PCSK9‐directed therapies.

**Materials and Methods:**

We searched PubMed, Embase, Web of Science and Cochrane CENTRAL through December 6, 2025, for randomised trials in adults (≥ 18 years) with ≥ 8‐week follow‐up reporting Lp(a). The primary outcome was placebo‐adjusted mean difference (MD) in percent change from baseline in Lp(a) (percentage points, pp). Secondary outcomes included LDL‐C, other lipid parameters and safety outcomes (injection‐site reactions, serious adverse events (SAEs), discontinuations). We performed a frequentist random‐effects network meta‐analysis in R (netmeta) and ranked interventions using P‐scores.

**Results:**

Fifty‐one trials (17 810 participants) formed a 16‐node network. Olpasiran 225 mg Q12W was associated with the greatest Lp(a) reduction versus placebo (MD −98.94 pp, 95% CI −114.36 to −83.52); pelacarsen, muvalaplin, zerlasiran and lepodisiran were also associated with large reductions. PCSK9‐directed therapies were associated with more modest Lp(a) reductions (evolocumab 140 mg Q2W: MD −31.58 pp), but greater LDL‐C lowering. The primary Lp(a) network showed high heterogeneity (*I* ^2^ = 90.9%) and funnel plot asymmetry, although treatment rankings remained directionally consistent across sensitivity analyses. No therapy was associated with higher SAEs or discontinuations versus placebo; alirocumab 150 mg was associated with more injection‐site reactions.

**Conclusions:**

Lp(a)‐targeted therapies were associated with larger Lp(a) reductions than PCSK9‐directed therapies, while PCSK9‐directed therapies had greater LDL‐C lowering. Given high heterogeneity, funnel plot asymmetry and low certainty for several estimates, these findings should be interpreted cautiously pending cardiovascular outcome trials.

## 1 Introduction

Lipoprotein(a) [Lp(a)] is a genetically determined lipoprotein and an independent risk factor for atherosclerotic cardiovascular disease (ASCVD), including coronary artery disease and ischemic stroke [1, 2, 3, 4]. Elevated Lp(a) carries a substantial cardiovascular burden: levels around 100 mg/dL have been associated with an approximately twofold higher ASCVD risk and levels around 175–180 mg/dL may confer ASCVD or myocardial infarction risk comparable to familial hypercholesterolemia; approximately 20%–25% of the global population have Lp(a) levels ≥ 50 mg/dL [5, 6]. Structurally, Lp(a) resembles an LDL particle but includes apolipoprotein(a) [apo(a)] covalently linked to apolipoprotein B100, which contributes to its atherogenic and prothrombotic properties [2, 3, 4, 7, 8]. In clinical practice, elevated Lp(a) often persists despite intensive guideline‐directed lipid lowering and may contribute to residual cardiovascular risk even among patients with otherwise well‐controlled LDL‐C [2, 3, 4].

Historically, pharmacologic options for reducing Lp(a) have been limited. Although statins remain essential for ASCVD prevention, they do not lower Lp(a) significantly, creating the demand for alternative strategies in patients with markedly elevated baseline levels [3, 4]. In contrast, therapies targeting the proprotein convertase subtilisin/kexin type 9 (PCSK9) pathway were developed primarily to lower LDL‐C by increasing hepatic LDL receptor recycling and enhancing clearance of apoB‐containing lipoproteins [4]. Monoclonal antibody PCSK9‐directed therapies (evolocumab, alirocumab) and the siRNA‐based PCSK9 synthesis inhibitor inclisiran have demonstrated clinically meaningful LDL‐C lowering in randomised trials and have become important adjunctive options for patients requiring additional reduction in atherogenic lipoproteins [4, 9]. Although not designed to target Lp(a), PCSK9‐directed therapies have also been associated with reductions in Lp(a); however, available estimates suggest the effect is generally modest and varies across agents, study populations, baseline Lp(a) concentrations and follow‐up duration [4, 10, 11]. The mechanisms underlying Lp(a) lowering with PCSK9‐directed therapies remain incompletely understood and it is unclear whether observed changes primarily reflect altered apo(a) production, enhanced clearance pathways, or broader remodelling of apoB‐containing lipoproteins [4, 10].

In parallel, several Lp(a)‐targeted therapies have emerged with mechanisms designed to directly suppress apo(a) production or disrupt Lp(a) particle assembly. Small‐interfering RNA (siRNA) agents, including olpasiran, zerlasiran and lepodisiran, reduce hepatic production of apo(a) and have shown large and durable reductions in Lp(a) in randomised trials [12, 13, 14, 15]. Antisense oligonucleotide therapy with pelacarsen similarly targets Lp(a) mRNA and has shown dose‐dependent lowering of Lp(a) in patients with established cardiovascular disease [16]. In addition, an oral approach has advanced with muvalaplin, a small‐molecule agent that inhibits Lp(a) formation by disrupting the apo(a)–apoB interaction and preventing its assembly, with randomised trials demonstrating substantial Lp(a) reduction and an overall tolerability profile that warrants comparison with injectable approaches [17, 18].

Given limited head‐to‐head data and cross‐trial differences in design and reporting, network meta‐analysis (NMA) can integrate direct and indirect evidence to estimate comparative effects [3, 11, 19]. Therefore, we conducted an NMA to compare the efficacy of emerging Lp(a)‐lowering therapies (olpasiran, zerlasiran, lepodisiran, pelacarsen and muvalaplin) with established PCSK9‐directed therapies (evolocumab, alirocumab and inclisiran) in reducing Lp(a) levels in adults with elevated Lp(a) and/or ASCVD, as defined by individual trial eligibility criteria. Secondary objectives included evaluating LDL‐C, total cholesterol, triglycerides and HDL‐C, as well as safety outcomes, including injection‐site reactions, serious adverse events and adverse events leading to discontinuation.

## 2 Materials and Methods

### 2.1 Protocol and Reporting Standards

This NMA was conducted in accordance with PRISMA‐NMA guidelines. The protocol was registered on the Open Science Framework (OSF Registration: 10.17605/OSF.IO/BDMKT) [20].

### 2.2 Eligibility Criteria

We included randomised controlled trials (RCTs) enrolling adults (≥ 18 years) with ≥ 8 weeks' follow‐up that reported Lp(a) as a primary or secondary outcome and evaluated at least one eligible intervention (alirocumab, evolocumab, inclisiran, olpasiran, zerlasiran, lepodisiran, pelacarsen or muvalaplin) versus placebo or standard background therapy. Among PCSK9‐directed therapies, only alirocumab, evolocumab and inclisiran were included, as these agents are the most extensively studied in RCTs, provide comparable data on lipoprotein(a) and allow for a connected evidence‐based network.

The primary outcome was the placebo‐adjusted mean difference in percent change from baseline in Lp(a), expressed in percentage points (pp). Secondary outcomes included placebo‐adjusted mean differences in percent change from baseline for LDL‐C, total cholesterol, triglycerides and HDL‐C and risk ratios for safety outcomes.

We excluded non‐randomised studies (including observational designs and case reports), trials not reporting Lp(a) and full texts lacking sufficient quantitative data to derive effect estimates (e.g., missing change values/dispersion or statistics to compute them) for the NMA. Detailed inclusion and exclusion criteria are provided in the study protocol [20].

### 2.3 Search Strategy and Study Selection

**FIGURE 1:**
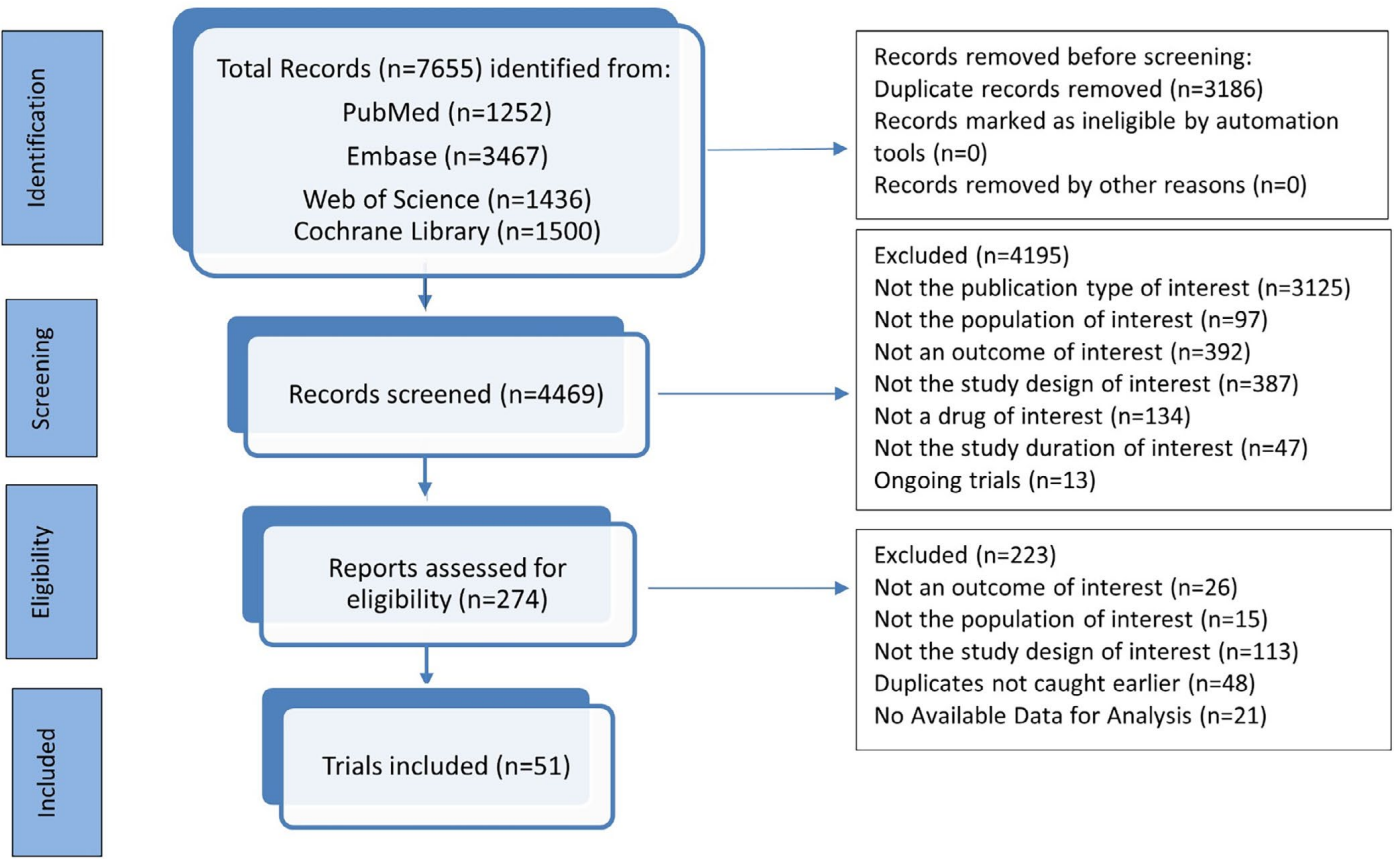
PRISMA flow diagram. Study identification, screening, eligibility and inclusion for the network meta‐analysis.

We systematically searched PubMed, Web of Science, Embase and the Cochrane Central Register of Controlled Trials (CENTRAL) from inception to 6 December 2025. The search strategy combined terms related to randomised controlled trials, PCSK9‐directed therapies and emerging Lp(a)‐lowering therapies, including antisense oligonucleotides, small interfering RNA (siRNA) agents and small‐molecule inhibitors, as well as terms related to Lp(a). For each intervention, the search included generic and brand names and employed free‐text keywords and, where available, controlled vocabulary terms (e.g., MeSH), combined using Boolean operators. No language or publication status restrictions were applied. Two independent reviewers screened titles/abstracts and full texts, resolving disagreements by consensus with a third reviewer as needed. The study selection process is shown in Figure 1 and the full electronic search strategy for all databases is provided in Supporting Information 1.

### 2.4 Data Extraction and Risk of Bias Assessment

Two independent reviewers extracted data using a standardised form covering study characteristics (trial phase, year, sample size, interventions/comparators), baseline patient characteristics and outcomes. In addition, we prospectively extracted study‐level covariates (mean age, baseline LDL‐C, baseline Lp(a), follow‐up duration, background lipid‐lowering therapy and study population characteristics) to enable exploratory meta‐regression analyses to investigate sources of heterogeneity and potential effect modification by baseline Lp(a). When trials reported outcomes in other formats (e.g., absolute change or endpoint values), we converted them to percent change from baseline; when SDs were not reported, we derived them from available statistics (e.g., SEs, CIs, medians/IQRs) using established methods [21, 22].

The Cochrane Risk of Bias 2 (RoB 2) tool was used to assess the risk of bias for each outcome across five domains: randomisation process, deviations from intended interventions, missing outcome data, measurement of the outcome and selection of the reported result. For each domain, the risk of bias was classified as low risk, some concerns, or high risk [23]. Overall confidence in the network estimates was evaluated using the Confidence in Network Meta‐Analysis (CINeMA) framework [24].

### 2.5 Statistical Analysis

We performed a frequentist random‐effects NMA using netmeta in R [25]. For each comparison, we calculated risk ratios (RRs) for dichotomous outcomes and mean differences (MDs) for continuous outcomes (pp for lipid outcomes) with 95% confidence intervals, using intention‐to‐treat data where available and applying a continuity correction of 0.5 for zero‐event cells. A random‐effects NMA with a common between‐study variance (τ^2^, estimated by the DerSimonian–Laird method) was fitted [26], synthesising direct and indirect evidence under the standard consistency assumption. Interventions were analysed as regimen‐specific nodes (dose + dosing interval). Placebo, administered alone or on background lipid‐lowering therapy per trial protocol, served as the reference intervention and treatments were ranked using P‐scores (0–1) from the netrank function, where higher values indicate more favourable outcomes.

### 2.6 Assessment of Heterogeneity, Publication Bias and Inconsistency

Heterogeneity across studies was assessed using the Cochran's *Q* test, the *I* ^2^ statistic and the between‐study variance (*τ* ^2^). We considered *I* ^2^ values of < 25% as low, 25%–49% as moderate, 50%–74% as substantial and ≥ 75% as high heterogeneity and interpreted *Q* with a significance threshold of *p* < 0.10 [27]. For key efficacy outcomes, we explored sources of heterogeneity using exploratory univariable meta‐regression models with trial phase, mean age, sample size, follow‐up duration, baseline Lp(a), background lipid‐lowering therapy and treatment class as study‐level covariates, alongside population‐based subgroup analyses, leave‐one‐out analyses and restriction to larger studies and/or trials at low risk of bias. The treatment class was interpreted cautiously because it was closely related to the primary comparison of interest. Global inconsistency was evaluated using the design‐by‐treatment interaction model. Prespecified sensitivity analyses included sequential leave‐one‐out analyses and exclusion of studies at high risk of bias to evaluate the influence of individual trials on overall estimates. Phase‐stratified analyses of the primary Lp(a) outcome were performed for phase 2 and phase 3 trials and are presented in Supporting Information 8; phase 1 and phase 4 trials were not analysed separately because of the limited number of studies and sparse network structure. Further, publication bias was assessed using comparison‐adjusted funnel plots. Where applicable, funnel‐plot asymmetry was further assessed using Egger's regression test.

## 3 Results

### 3.1 Study Characteristics

In total, 51 randomised controlled trials comprising 17 810 participants were included [13, 14, 15, 16, 18, 28, 29, 30, 31, 32, 33, 34, 35, 36, 37, 38, 39, 40, 41, 42, 43, 44, 45, 46, 47, 48, 49, 50, 51, 52, 53, 54, 55, 56, 57, 58, 59, 60, 61, 62, 63, 64, 65, 66, 67, 68, 69, 70, 71, 72]. Follow‐up duration ranged from 8 to 78 weeks (median 24 weeks; IQR 12–48). The primary outcome was the placebo‐adjusted mean difference in percent change in Lp(a) (pp) and the same metric was used for all continuous lipid outcomes. Network plots for lipid and safety outcomes are provided in Supporting Information 2. Total cholesterol and HDL‐C results are reported in Supporting Information 8.2 and Supporting Information 8.3, respectively.

The mean age in the included studies was 59.31 years. For Lp(a), baseline levels were reported in two different units; the mean baseline level was 96.72 nmol/L (SD 98.45) in studies reporting nmol/L and 31.67 mg/dL (SD 43.75) in studies reporting mg/dL. The mean baseline LDL‐C level was 118.41 mg/dL (SD 36.84). The detailed characteristics of studies and patients' baseline characteristics are presented in Supporting Information 3.

### 3.2 Lp(a)

**FIGURE 2:**
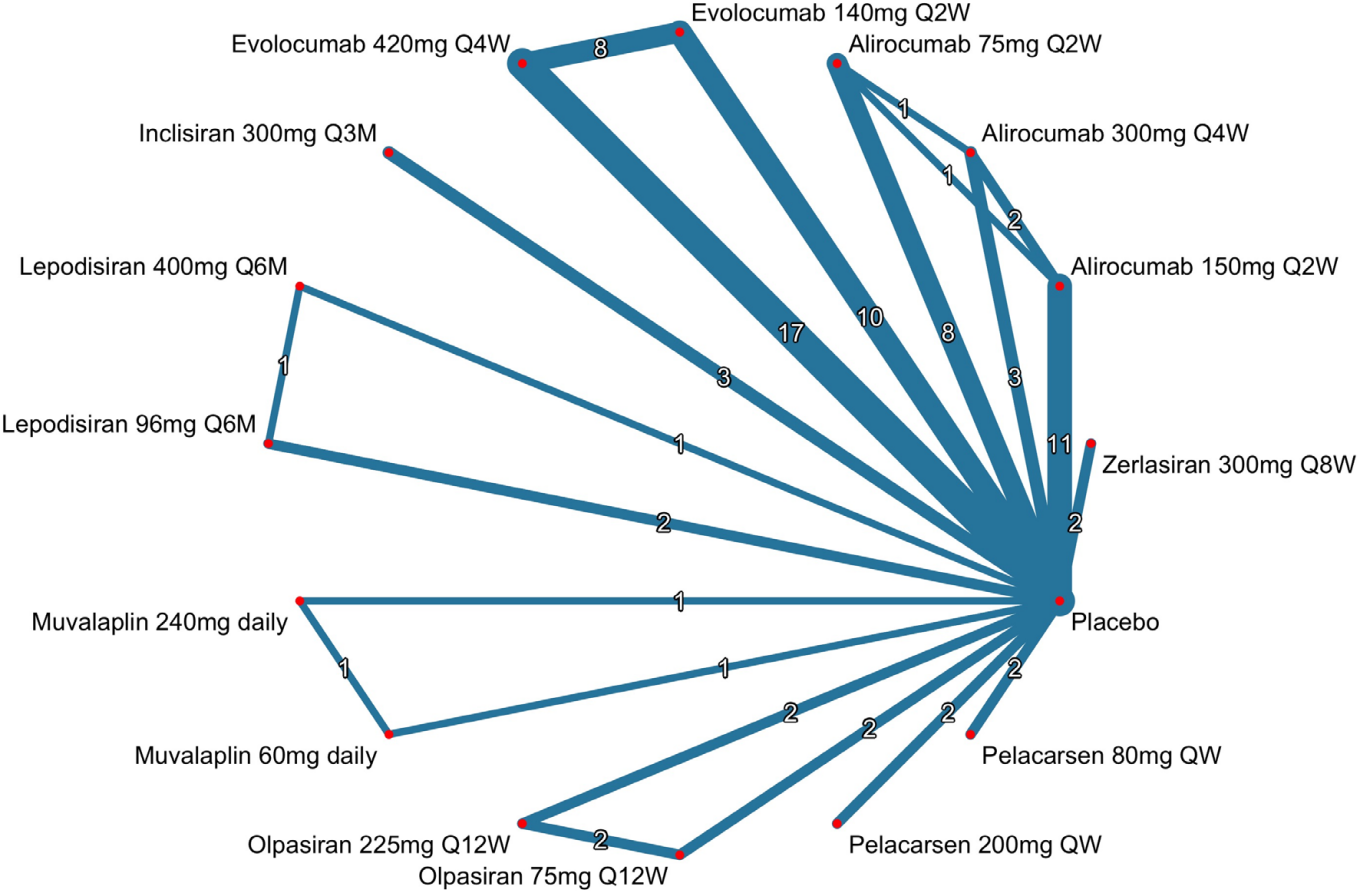
Treatment network for lipoprotein(a). Nodes represent interventions. Lines denote direct comparisons between interventions; numbers on the lines indicate the number of trials contributing to each comparison and line thickness is proportional to that number.

**FIGURE 3:**
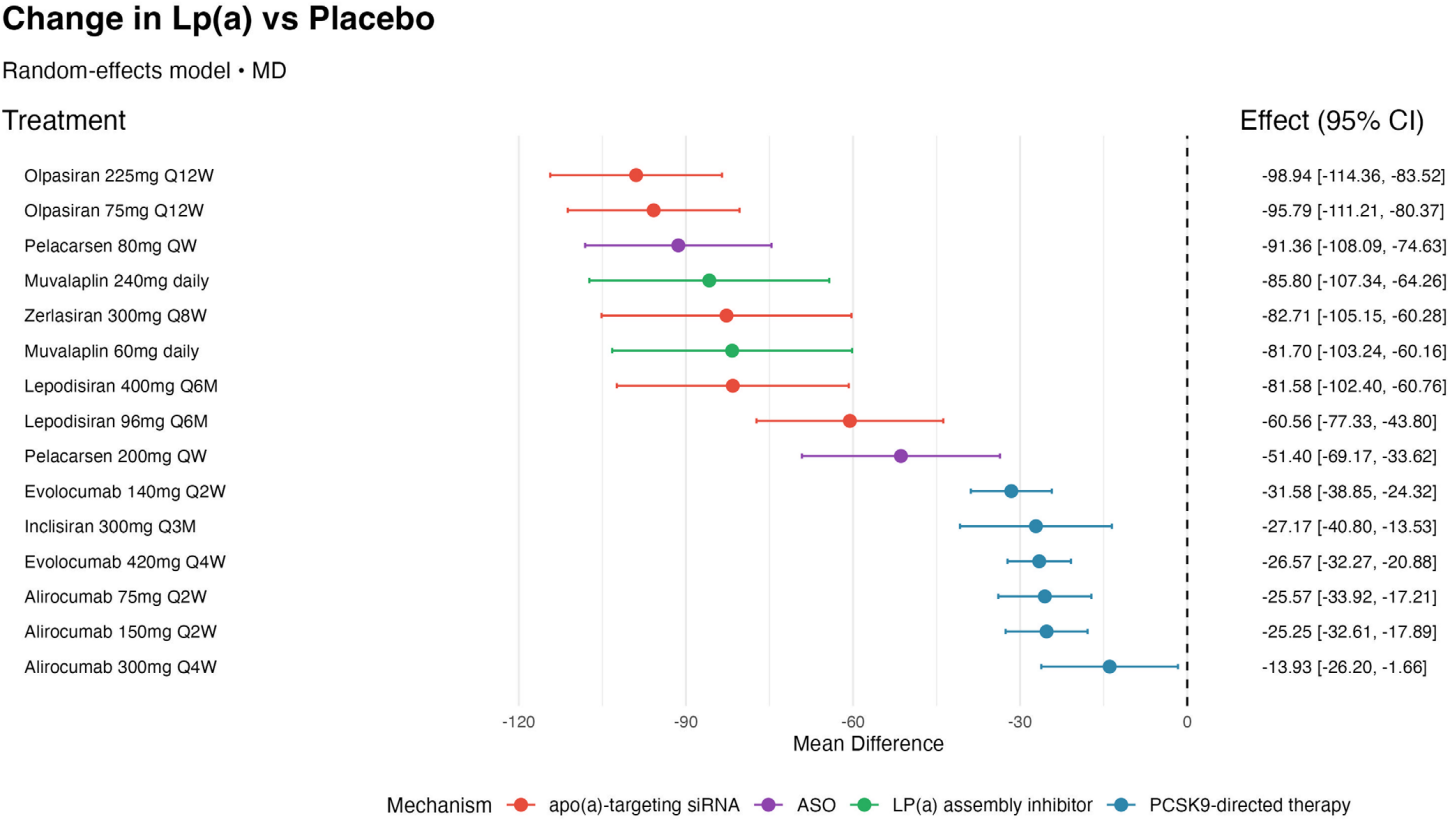
Network meta‐analysis estimates for Lp(a) versus placebo. Effect sizes are presented as placebo‐adjusted mean differences (MDs) in percent change from baseline, expressed in percentage points (pp), with 95% confidence intervals (CIs). More negative values indicate greater Lp(a) reduction. Treatments are colour‐coded by drug class/mechanism; inclisiran was classified as a PCSK9‐directed therapy. QW weekly; Q2W every 2 weeks; Q4W every 4 weeks; Q8W every 8 weeks; Q12W every 12 weeks; Q3M every 3 months; Q6M every 6 months.

The treatment comparison network consists of 16 nodes representing 15 active therapies and a placebo (Figure 2; pairwise NMA results are presented in Supporting Information 6). Full regimen‐specific estimates versus placebo are shown in Figure 3. Because head‐to‐head active–active trials were limited, several active–active comparisons were informed primarily by indirect evidence through the placebo node (Figure 2). Olpasiran 225 mg Q12W was associated with the greatest Lp(a) reduction (MD −98.94, 95% CI −114.36 to −83.52), followed by olpasiran 75 mg Q12W (MD −95.79, 95% CI −111.21 to −80.37). Pelacarsen 80 mg QW was also associated with a large reduction (MD −91.36, 95% CI −108.09 to −74.63), along with muvalaplin 240 mg daily (MD −85.80, 95% CI −107.34 to −64.26) and zerlasiran 300 mg Q8W (MD −82.71, 95% CI −105.15 to −60.28). Lepodisiran was associated with substantial dose‐related effects, with 400 mg Q6M (MD −81.58, 95% CI −102.40 to −60.76) exceeding 96 mg Q6M (MD −60.56, 95% CI −77.33 to −43.80), while pelacarsen 200 mg QW was associated with a more modest reduction (MD −51.40, 95% CI −69.17 to −33.62). Among PCSK9‐directed therapies, evolocumab 140 mg Q2W was associated with the greatest reduction (MD −31.58, 95% CI −38.85 to −24.32), followed by inclisiran 300 mg Q3M (MD −27.17, 95% CI −40.80 to −13.53). These findings were mirrored by ranking analyses, with olpasiran 225 mg Q12W and olpasiran 75 mg Q12W ranking highest among interventions (P‐scores 0.93 and 0.89, respectively), followed by pelacarsen 80 mg QW (P‐score 0.83) and muvalaplin 240 mg daily (P‐score 0.77). Higher P‐scores indicate larger placebo‐adjusted reductions in Lp(a).

Between‐study heterogeneity was high (*I* ^2^ = 90.9%, 95% CI 88.8%–92.5%; *τ* ^2^ = 119.16). Nevertheless, treatment effects and rankings remained directionally consistent across multiple sensitivity analyses, including exclusion of high–risk–of–bias trials (*k* = 50), restriction to larger studies (*k* = 29), common‐effects modelling and leave‐one‐out analyses. Notably, heterogeneity was similar after exclusion of high‐risk‐of‐bias trials with evidence of between‐design inconsistency (*Q* = 103.45, df = 8, *p* < 0.001), whereas no between‐design inconsistency was detected in the large‐study restriction (*Q* = 2.95, df = 2, *p* = 0.229). This pattern suggests that inconsistency may be influenced by smaller and/or earlier‐phase trials included in the full network. Phase‐stratified findings are provided in Supporting Information 8. Exploratory meta‐regression identified trial phase, baseline Lp(a), background PCSK9 therapy and treatment class as covariates associated with treatment effect, while mean age, sample size, follow‐up duration, background statin use and background ezetimibe use were not statistically significant. Exploratory population‐based subgroup analyses showed that heterogeneity varied across clinical populations, with lower heterogeneity in the CAD subgroup (*I* ^2^ = 59.3%) and no detected heterogeneity in familial hypercholesterolemia (*I* ^2^ = 0%), but persistent high heterogeneity in the elevated Lp(a) subgroup (*I* ^2^ = 92.7%) and hypercholesterolemia subgroup (*I* ^2^ = 79.5%). Full meta‐regression and subgroup analyses are provided in Supporting Information 8.

Overall certainty of evidence was low for placebo‐controlled comparisons and very low for most active–active comparisons using the CINeMA framework, mainly due to heterogeneity and imprecision, with additional concerns for reporting bias in RNA‐targeting therapy comparisons (Supporting Information 11). Egger's test indicated significant funnel‐plot asymmetry (test statistic = −7.28, *p* < 0.001), raising concern for small‐study effects, including possible publication bias (Supporting Information 5). Accordingly, active–active estimates and rankings should be interpreted cautiously, particularly where confidence intervals are wide.

### 3.3 LDL‐C

**FIGURE 4:**
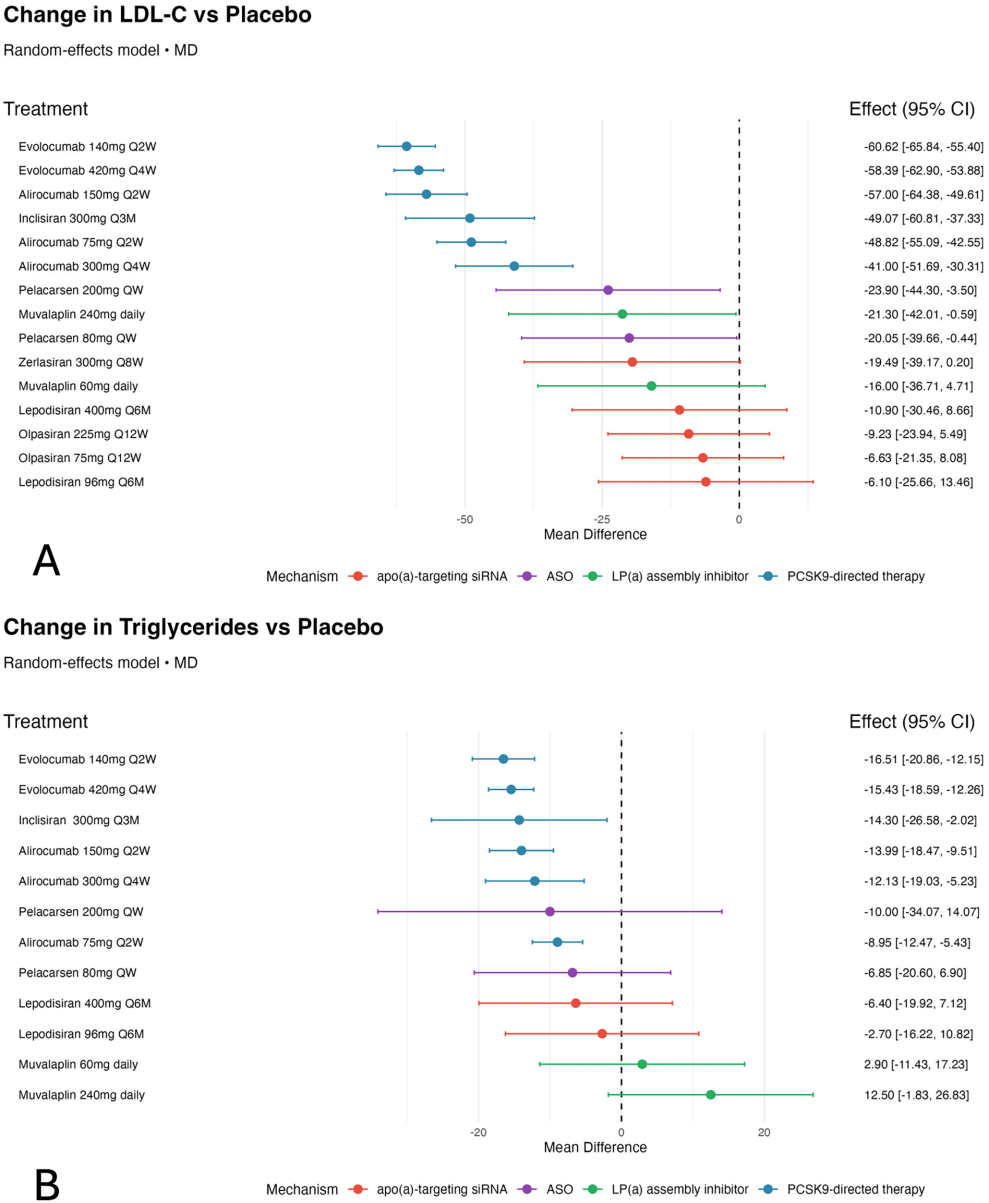
(A) Network meta‐analysis estimates for LDL‐C versus placebo. Effect sizes are presented as placebo‐adjusted MDs in percent change from baseline (pp) with 95% CIs. More negative values indicate greater LDL‐C reduction. Treatments are colour‐coded by drug class/mechanism; inclisiran was classified as a PCSK9‐directed therapy. Abbreviations: QW weekly; Q2W every 2 weeks; Q4W every 4 weeks; Q8W every 8 weeks; Q12W every 12 weeks; Q3M every 3 months; Q6M every 6 months. (B) Network meta‐analysis estimates for triglycerides versus placebo. Effect sizes are presented as placebo‐adjusted MDs in percent change from baseline (pp) with 95% CIs. More negative values indicate greater triglyceride reduction. Treatments are colour‐coded by drug class/mechanism; inclisiran was classified as a PCSK9‐directed therapy. QW weekly; Q2W every 2 weeks; Q4W every 4 weeks; Q12W every 12 weeks; Q3M every 3 months; Q6M every 6 months.

The LDL‐C treatment network consists of 16 nodes representing 15 therapies and a placebo (full regimen‐specific estimates are shown in Figure 4A; detailed pairwise and network estimates are provided in Supporting Information 2 and 6). Evolocumab 140 mg Q2W was associated with the greatest LDL‐C reduction versus placebo (MD −60.62, 95% CI −65.84 to −55.40), followed by evolocumab 420 mg Q4W (MD −58.39, 95% CI −62.90 to −53.88) and alirocumab 150 mg Q2W (MD −57.00, 95% CI −64.38 to −49.61). Among emerging Lp(a) therapies, LDL‐C reductions were smaller but statistically significant with pelacarsen 200 mg QW (MD −23.90, 95% CI −44.30 to −3.50), muvalaplin 240 mg daily (MD −21.30, 95% CI −42.01 to −0.59) and pelacarsen 80 mg QW (MD −20.05, 95% CI −39.66 to −0.44). In contrast, other Lp(a)‐targeted agents were not associated with statistically significant LDL‐C lowering. These findings were reflected in treatment rankings, with evolocumab 140 mg Q2W ranking highest (P‐score 0.97), followed by evolocumab 420 mg Q4W (P‐score 0.92) and alirocumab 150 mg Q2W (P‐score 0.89).

Between‐study heterogeneity was high (*I* ^2^ = 87.6%, 95% CI 84.6%–90.1%; *τ* ^2^ = 80.27). However, sensitivity analyses (high–risk–of–bias exclusion, large‐study restriction, common‐effects and leave‐one‐out) yielded robust, directionally consistent findings. Exploratory meta‐regression identified trial phase as a significant modifier of treatment effect (*β* = −15.10, SE = 4.20, *p* < 0.001), indicating systematically larger LDL‐C reductions in earlier‐phase trials, while other prespecified covariates (mean age, sample size and follow‐up duration) were not statistically significant. The trial phase explained part of the variability in effect estimates across studies. CINeMA confidence was low to very low (Supporting Information 11) and funnel‐plot asymmetry suggested small‐study effects (Supporting Information 5).

### 3.4 Triglycerides (TG)

The TG treatment network consisted of 13 nodes, representing 12 active therapies and placebo (full regimen‐specific estimates are shown in Figure 4B; detailed pairwise and network estimates are available in Supporting Information 2 and 6). Evolocumab 140 mg Q2W was associated with the greatest reduction versus placebo (MD −16.51, 95% CI −20.86 to −12.15), followed by evolocumab 420 mg Q4W (MD −15.43, 95% CI −18.59 to −12.26) and inclisiran 300 mg Q3M (MD −14.30, 95% CI −26.58 to −2.02). In contrast, emerging Lp(a) therapies were not associated with statistically significant triglyceride lowering. Ranking analyses supported these results, with evolocumab 140 mg Q2W ranked highest (P‐score 0.87), followed by evolocumab 420 mg Q4W (P‐score 0.82) and inclisiran 300 mg Q3M (P‐score 0.73).

Between‐study heterogeneity was moderate (*I* ^2^ = 42.7%, 95% CI 17.2%–60.4%; *τ* ^2^ = 13.72), largely due to within‐design variability in large placebo–evolocumab and placebo–alirocumab comparisons. Sensitivity and leave‐one‐out analyses produced consistent estimates and rankings. No between‐design inconsistency was detected; funnel‐plot asymmetry suggested small‐study effects (Supporting Information 5). Compared with Lp(a) and LDL‐C networks, heterogeneity was lower for TG, likely reflecting more consistent measurement and broader evidence within large placebo‐controlled PCSK9 comparisons. CINeMA confidence was low to very low across most comparisons (Supporting Information 11), primarily due to imprecision and heterogeneity, while no major concerns for incoherence or indirectness were identified.

### 3.5 Safety Outcomes

#### 3.5.1 Injection Site Reactions

**FIGURE 5:**
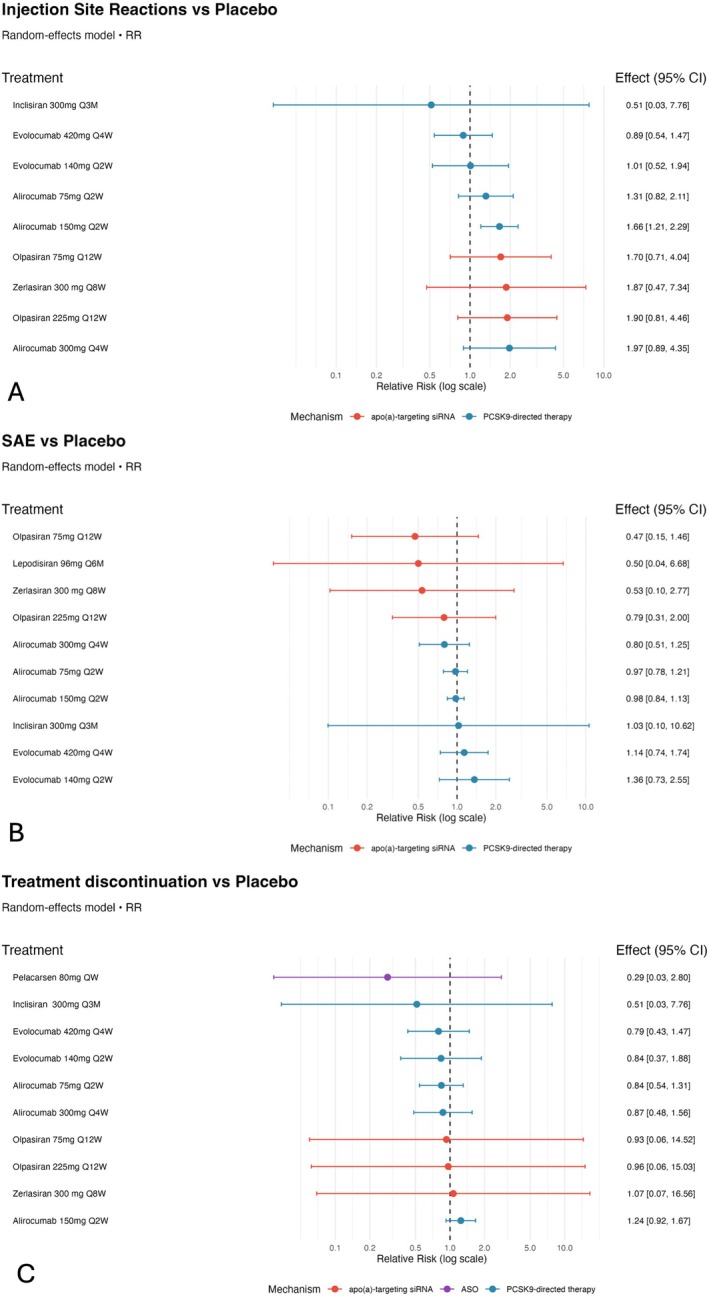
(A) Network meta‐analysis of injection‐site reactions versus placebo. Effect sizes are presented as risk ratios (RRs) with 95% CIs; RR > 1 indicates higher risk. Events reflect participants with ≥ 1 event as defined by each trial. Treatments are colour‐coded by drug class/mechanism; inclisiran was classified as a PCSK9‐directed therapy. Q2W every 2 weeks; Q4W every 4 weeks; Q8W every 8 weeks; Q12W every 12 weeks; Q3M every 3 months. (B) Serious adverse events versus placebo. Effect sizes are presented as RRs with 95% CIs; RR > 1 indicates higher risk. Events reflect participants with ≥ 1 event as defined by each trial. Treatments are colour‐coded by drug class/mechanism; inclisiran was classified as a PCSK9‐directed therapy. Q2W every 2 weeks; Q4W every 4 weeks; Q8W every 8 weeks; Q12W every 12 weeks; Q3M every 3 months; Q6M every 6 months. (C) Treatment discontinuation due to adverse events versus placebo. Effect sizes are presented as RRs with 95% CIs; RR > 1 indicates higher risk. Events reflect participants with ≥ 1 event as defined by each trial. Treatments are colour‐coded by drug class/mechanism; inclisiran was classified as a PCSK9‐directed therapy. QW weekly; Q2W every 2 weeks; Q4W every 4 weeks; Q8W every 8 weeks; Q12W every 12 weeks; Q3M every 3 months.

Injection‐site reactions were reported in 32 randomised controlled trials. Injection‐site reaction analyses included injectable therapies only; oral therapy was not applicable. Alirocumab 150 mg Q2W was the only agent associated with a statistically significant increase in injection‐site reactions compared with placebo (RR 1.66, 95% CI 1.21 to 2.29). Effect estimates with 95% CIs for each intervention versus placebo are shown in Figure 5A and Supporting Information 6.

Heterogeneity and inconsistency were negligible (*τ* ^2^ = 0; *I* ^2^ = 0.0% [0.0%–43.9%]). Findings were consistent in sensitivity analyses (excluding high‐risk‐of‐bias and small studies and leave‐one‐out analyses) and meta‐regression did not identify effect modification (all *p* > 0.05), supporting the robustness of these results.

#### 3.5.2 Serious Adverse Events (SAEs)

SAEs were reported in 33 trials. In the random‐effects NMA, all agents showed no statistically significant difference in SAEs compared with placebo. Effect estimates with 95% CIs for each intervention versus placebo are shown in Figure 5B and Supporting Information 6. Given the wide confidence intervals and low event counts typical of SAE outcomes across trials, these estimates should be interpreted cautiously. Between‐study heterogeneity and inconsistency were negligible (*τ* ^2^ = 0.000; *I* ^2^ = 0.0% [0.0%–38.7%]; *Q* = 21.94, df = 33, *p* = 0.929).

#### 3.5.3 Treatment Discontinuation due to Adverse Events (DAEs)

Thirty‐two randomised controlled trials were included for DAEs. No agents showed a statistically significant difference in DAEs compared with placebo. Effect estimates with 95% CIs are shown in Figure 5C and Supporting Information 6.

Between‐study heterogeneity and inconsistency were negligible (*τ* ^2^ = 0; *I* ^2^ = 0%). Sensitivity analyses (excluding high‐risk‐of‐bias and small studies and leave‐one‐out analyses) yielded consistent findings and meta‐regression did not identify significant effect modification (all *p* > 0.05), supporting the robustness of these findings. Because DAE events were uncommon, these rankings should be interpreted cautiously and alongside the corresponding confidence intervals.

## 4 Discussion

To our knowledge, this is the first NMA comparing emerging Lp(a)‐lowering therapies with PCSK9‐directed therapies. Emerging therapies included RNA‐based agents that suppress hepatic apo(a) production (pelacarsen; siRNAs such as olpasiran, zerlasiran and lepodisiran) and the oral Lp(a) assembly inhibitor muvalaplin. Three main themes emerged from this pooled analysis. First, emerging therapies were associated with larger placebo‐adjusted Lp(a) reductions than PCSK9‐directed therapies; between‐class differences were inferred largely from indirect evidence. Second, LDL‐C and total cholesterol reductions were largely driven by PCSK9‐directed therapies. Among emerging therapies, only pelacarsen was associated with modest LDL‐C reductions; other Lp(a)‐targeted agents were not. Third, PCSK9‐directed therapies were associated with modest triglyceride reductions and HDL‐C increases, whereas emerging therapies were not associated with statistically significant effects. Accordingly, the distinct therapeutic classes exhibit different effects across lipid parameters, consistent with their distinct mechanisms of action.

In exploratory P‐score rankings, olpasiran, pelacarsen and muvalaplin ranked highest for Lp(a) lowering. These findings were concordant with the existing literature, in which olpasiran achieved placebo‐adjusted Lp(a) reductions approaching 90% to 100% in the OCEAN[a]‐DOSE trial, while pelacarsen reported reductions of approximately 80% to 90% in a phase 2B trial and muvalaplin demonstrated dose‐dependent reductions of 45%–65% in a phase 1 trial. Zerlasiran and lepodisiran followed, while PCSK9‐directed therapies were associated with more modest reductions [17, 66, 73].

In contrast, PCSK9‐directed therapies showed a more predictable lipid profile, characterised by substantial LDL‐C reductions consistent with the current analysis. Evolocumab (140 mg, 420 mg), alirocumab (75 mg, 150 mg) and inclisiran (300 mg) were among the most effective therapies in LDL‐C reduction, consistent with findings from FOURIER/earlier studies, ODYSSEY OUTCOMES and ORION‐10/11 trials, respectively [9, 74, 75]. Clinically, these findings suggest that PCSK9‐directed therapies and emerging Lp(a)‐lowering agents may have complementary rather than interchangeable roles. PCSK9‐directed therapies remain most relevant for patients with residual LDL‐C–mediated risk, whereas emerging Lp(a)‐lowering therapies may be considered as ‘add‐on precision’ therapies aimed at lowering Lp(a) in patients with markedly elevated Lp(a) despite optimised LDL‐C lowering. For patients with mixed dyslipidemia, the broader lipid effects across LDL‐C, triglycerides and HDL‐C may also influence therapeutic selection. Accordingly, these clinical implications should be considered hypothesis‐generating [7, 76].

Regarding safety, no PCSK9‐directed or Lp(a)‐targeting therapy was associated with an increased risk of SAEs or DAEs versus placebo; heterogeneity was negligible and findings were consistent across sensitivity analyses and meta‐regression. This is consistent with findings from the FOURIER and ODYSSEY trials [74, 75]. Furthermore, injection‐site reactions were generally similar to placebo; however, alirocumab 150 mg was associated with an increased risk of injection‐site reactions. This aligns with prior PCSK9 inhibitor trials, in which injection‐site reactions were among the more common adverse events but rarely led to discontinuation [77].

Across outcomes, results were directionally consistent in sensitivity analyses, although heterogeneity varied by endpoint and was high in the primary Lp(a) network. Nonetheless, the wide prediction intervals suggest that variations could stem from different clinical settings and trial populations, namely baseline Lp(a), background lipid‐lowering therapy, trial phase and population characteristics. Furthermore, inter‐individual variability may plausibly relate to apo(a) isoform size, a genetically determined feature that may influence baseline Lp(a), as larger isoforms have been associated with greater proportional reduction in Lp(a) following administration of PCSK9‐directed therapies, as suggested by the CHORD study [78]. Heterogeneity was further explored through meta‐regression and subgroup analyses, which suggested that some of these factors may partly contribute to between‐study variability, although residual heterogeneity remained. Moreover, funnel plot asymmetry was observed, which may reflect small‐study effects or publication bias. Together, these findings suggest that treatment rankings and indirect comparisons should be interpreted cautiously, highlighting the need for larger phase 3 trials to refine effect estimates and reduce uncertainty.

Our NMA comprised several strengths. First, a comprehensive search was conducted on a variety of databases, with independent screening and data extraction to minimise bias. Second, our approach allowed comparison of multiple interventions and assessment of consistency across evidence sources. Third, results remained directionally consistent after extensive sensitivity analysis and meta‐regression, with no material changes in conclusions.

Nonetheless, this analysis was limited by the inclusion of early‐phase trials with relatively short follow‐up, sparse evidence for some treatment nodes and residual heterogeneity despite sensitivity and consistency analyses. The very low certainty ratings in CINeMA indicate limited confidence in many comparative estimates, particularly active–active comparisons; therefore, these findings should be viewed as hypothesis‐generating rather than sufficient to guide clinical selection between therapies. Lastly, our analysis highlights lipid outcomes and lipoprotein biomarkers, which represent surrogate outcomes rather than clinical cardiovascular outcomes. Definitive evidence is still emerging and awaits the results of ongoing phase 3 outcome trials [79, 80].

In conclusion, emerging Lp(a)‐targeted therapies were associated with substantially larger Lp(a) reductions than PCSK9‐directed therapies, whereas PCSK9‐directed therapies showed broader LDL‐C–lowering effects. These findings support a potentially complementary role for these therapeutic classes, but they should be interpreted cautiously given the high heterogeneity, funnel‐plot asymmetry and low certainty for several comparisons. Future cardiovascular outcome data will be essential to determine whether these lipid changes translate into clinical benefit.

## Funding

The authors have nothing to report.

## Conflicts of Interest

The authors declare no conflicts of interest.

## Supporting information


**Data S1:** Supporting Information.

**Supporting Information: 1.** Search strategy.
**Supporting Information: 2** Network figures.
**Supporting Information: 3** Study and population characteristics table.
**Supporting Information: 4** Risk of bias assessment.
**Supporting Information: 5** Publication bias (funnel plot).
**Supporting Information: 6** Network meta‐analysis results (league tables).
**Supporting Information: 7** Sensitivity analysis.
**Supporting Information: 8** Supplementary results and exploratory heterogeneity analyses.
**Supporting Information: 9** PRISMA flow diagram.
**Supporting Information: 10** PRISMA checklist.

## Data Availability

The data supporting the findings of this study were extracted from previously published randomised clinical trials. Extracted data used for the analyses are provided in the article and Supporting Information. Additional extracted datasets and analytic code are available from the corresponding author upon reasonable request.
