# Supplementary material for: Comparative Effects of Emerging Lp(a)‐Lowering Agents and PCSK9‐Directed Therapies on Lipoprotein(a): A Network Meta‐Analysis of Randomised Clinical Trials
Source: Diabetes Obes Metab. 2026 Jun 4;28(8):7403–15. doi: 10.1111/dom.70944 (PMC13341347; doi:10.1111/dom.70944)
Supplement: Supplementary file 2 — Supporting Information: 1. Search strategy. Supporting Information: 2 Network figures. Supporting Information: 3 Study and population characteristics table. Supporting Information: 4 Risk of bias assessment. Supporting Information: 5 Publication bias (funnel plot). Supporting Information: 6 Network meta‐analysis results (league tables). Supporting Information: 7 Sensitivity analysis. Supporting Information: 8 Supplementary results and exploratory heterogeneity analyses. Supporting Information: 9 PRISMA flow diagram. Supporting Information: 10 PRISMA checklist. [file DOM-28-7403-s001.docx]

**Supplementary Material**

Table of Contents

[**Supplement 1. Search strategy** 4](#_Toc228838624)

[**Supplement 2. Network figures** 7](#_Toc228838625)

[**2.1. Lp(a)** 7](#_Toc228838626)

[**2.2. Lp(a) - large sample** 8](#_Toc228838627)

[**2.3. Lp(a) - low risk** 9](#_Toc228838628)

[**2.4. LDL-C** 11](#_Toc228838629)

[**2.7. TG** 12](#_Toc228838630)

[**2.10. Total cholesterol** 13](#_Toc228838631)

[**2.13. HDL** 14](#_Toc228838632)

[**2.16. Adverse events** 15](#_Toc228838633)

[2.16.1. Injection site reactions 15](#_Toc228838634)

[2.16.2. Injection site reactions - large sample 15](#_Toc228838635)

[2.16.3 Injection site reactions - low risk 16](#_Toc228838636)

[2.16.4. Serious adverse events 18](#_Toc228838637)

[2.16.5. Serious adverse events - large sample 19](#_Toc228838638)

[2.16.6. Serious adverse events - low risk 19](#_Toc228838639)

[2.16.7. Treatment discontinuation due to adverse events 20](#_Toc228838640)

[2.16.8. Treatment discontinuation due to adverse events - large sample 21](#_Toc228838641)

[2.16.9. Treatment discontinuation due to adverse events - low risk 22](#_Toc228838642)

[**Supplement 3. Study and Population Characteristics Table** 24](#_Toc228838643)

[**Supplement 4. Risk of Bias Assessment** 31](#_Toc228838644)

[**Supplement 5. Publication bias (funnel plot)** 35](#_Toc228838645)

[**5.1. Lp(a)** 35](#_Toc228838646)

[**5.2. Lp(a) fixed** 36](#_Toc228838647)

[**5.3. Lp(a) Low risk** 37](#_Toc228838648)

[**5.4. Lp(a) Large sample** 38](#_Toc228838649)

[**5.5. LDL-C** 39](#_Toc228838650)

[**5.6. LDL-C fixed** 40](#_Toc228838651)

[**5.7. LDL-C Low risk** 41](#_Toc228838652)

[**5.8. LDL-C Large sample** 42](#_Toc228838653)

[**5.9. TG** 43](#_Toc228838654)

[**5.10. TG fixed** 44](#_Toc228838655)

[**5.11. TG Low risk** 45](#_Toc228838656)

[**5.12. TG Large sample** 46](#_Toc228838657)

[**5.13. Total Cholesterol** 47](#_Toc228838658)

[**5.14. Total Cholesterol fixed** 48](#_Toc228838659)

[**5.15. Total Cholesterol Low risk** 49](#_Toc228838660)

[**5.16. Total Cholesterol Large sample** 50](#_Toc228838661)

[**5.17. HDL** 51](#_Toc228838662)

[**5.18. HDL fixed** 52](#_Toc228838663)

[**5.19. HDL Low risk** 53](#_Toc228838664)

[**5.20. HDL Large sample** 54](#_Toc228838665)

[**5.21. Adverse events** 55](#_Toc228838666)

[**5.21.1. Injection site reactions** 55](#_Toc228838667)

[**5.21.2. Injection site reactions- large sample** 56](#_Toc228838668)

[5.21.3. Injection site reactions – low-risk 56](#_Toc228838669)

[5.21.4. Injection site reactions- fixed 57](#_Toc228838670)

[5.21.5. Serious adverse events 59](#_Toc228838671)

[5.21.6. Serious adverse events- fixed 60](#_Toc228838672)

[5.21.7. Serious adverse events- large sample 61](#_Toc228838673)

[5.21.8. Serious adverse events- low risk 63](#_Toc228838674)

[5.21.9. Treatment discontinuation due to adverse events 65](#_Toc228838675)

[5.21.10. Treatment discontinuation due to adverse events- fixed 66](#_Toc228838676)

[5.21.11. Treatment discontinuation due to adverse events- large sample 67](#_Toc228838677)

[5.21.12. Treatment discontinuation due to adverse events - low risk 68](#_Toc228838678)

[**Supplement 6. Network meta-analysis results (league tables)** 70](#_Toc228838679)

[**6.1. Network meta-analysis results for change in Lp(a)** 70](#_Toc228838680)

[**6.2. Network meta-analysis results for change in LDL-C** 73](#_Toc228838681)

[**6.3. Network meta-analysis results for change in TG** 76](#_Toc228838682)

[**6.4. Network meta-analysis results for change in Total Cholesterol** 78](#_Toc228838683)

[**6.5. Network meta-analysis results for change in HDL-C** 80](#_Toc228838684)

[**6.6. Adverse events** 82](#_Toc228838685)

[6.6.1 Injection site reactions 82](#_Toc228838686)

[6.6.2 Serious adverse events 83](#_Toc228838687)

[6.6.3 Treatment discontinuation due to adverse events 84](#_Toc228838688)

[**Supplement 7. Sensitivity Analysis** 86](#_Toc228838689)

[**Supplement 8. Supplementary Results and Exploratory Heterogeneity Analyses** 87](#_Toc228838690)

[**8.1.** **Exploratory heterogeneity analyses for the primary Lp(a) outcome** 87](#_Toc228838691)

[**8.1.1. Phase-stratified analyses** 88](#_Toc228838692)

[**8.1.2. Univariable meta-regression analyses** 94](#_Toc228838693)

[**8.1.3. Population-based subgroup analyses** 95](#_Toc228838694)

[**8.2. Total cholesterol** 96](#_Toc228838695)

[**8.3. HDL-C** 98](#_Toc228838696)

[**Supplement 9. PRISMA Flow Diagram** 100](#_Toc228838697)

[**Supplement 10. PRISMA Checklist** 100](#_Toc228838698)

# **Supplement 1. Search strategy**

| **Database** | **Search Dates** | **Search Query** | **Records Retrieved**  **(Total number= 7655)** |
| --- | --- | --- | --- |
| **PubMed** | Start date: NA  End date:  December 6, 2025 | ("PCSK9 Inhibitors" OR "PCSK9 inhibitor" OR "PCSK9 antibody") OR ("Alirocumab" OR "REGN727" OR "SAR236553" OR "Praluent" OR "Evolocumab" OR "Repatha" OR "AMG145" OR "AMG 145" OR "Inclisiran" OR "Leqvio" OR "ALN-PCSsc" OR "ALN PCSSC" OR "Olpasiran" OR "AMG 890" OR "Zerlasiran" OR "SLN360" OR "SLN-360" OR "Lepodisiran" OR "LY3819469" OR "Pelacarsen" OR "TQJ230" OR "AKCEA-APO(a)-LRx" OR "IONIS-APO(a)-LRx" OR "Muvalaplin" OR "LY3473329") AND ("Lipoprotein(a)" OR "Lipoprotein a" OR "Lp(a)" OR "Low-Density Lipoprotein" OR "LDL") AND (Clinical Trial OR randomized OR "trial") | 1252 |
| **Web of Science** | Start date: NA  End date:  December 6, 2025 | TS=( "PCSK9 Inhibitors" OR "PCSK9 inhibitor" OR "PCSK9 antibody" OR "Alirocumab" OR "REGN727" OR "SAR236553" OR "Praluent" OR "Evolocumab" OR "Repatha" OR "AMG145" OR "AMG 145" OR "Inclisiran" OR "Leqvio" OR "ALN-PCSsc" OR "ALN PCSSC" OR "Olpasiran" OR "AMG 890" OR "Zerlasiran" OR "SLN360" OR "SLN-360" OR "Lepodisiran" OR "LY3819469" OR "Pelacarsen" OR "TQJ230" OR "AKCEA-APO(a)-LRx" OR "IONIS-APO(a)-LRx" OR "Muvalaplin" OR "LY3473329" ) AND TS=( "Lipoprotein(a)" OR "Lipoprotein a" OR "Lp(a)" OR "Low-Density Lipoprotein" OR "LDL" ) AND TS=( Clinical Trial OR randomized OR "trial" ) | 1436 |
| **Embase** | Start date: NA  End date: December 6, 2025 | ("PCSK9 Inhibitors" OR "PCSK9 inhibitor" OR "PCSK9 antibody") OR ("Alirocumab" OR "REGN727" OR "SAR236553" OR "Praluent" OR "Evolocumab" OR "Repatha" OR "AMG145" OR "AMG 145" OR "Inclisiran" OR "Leqvio" OR "ALN-PCSsc" OR "ALN PCSSC" OR "Olpasiran" OR "AMG 890" OR "Zerlasiran" OR "SLN360" OR "SLN-360" OR "Lepodisiran" OR "LY3819469" OR "Pelacarsen" OR "TQJ230" OR "AKCEA-APO(a)-LRx" OR "IONIS-APO(a)-LRx" OR "Muvalaplin" OR "LY3473329") AND ("Lipoprotein(a)" OR "Lipoprotein a" OR "Lp(a)" OR "Low-Density Lipoprotein" OR "LDL") AND (Clinical Trial OR randomized OR "trial") | 3467 |
| **Cochrane Library** | Start date: NA  End date: December 6, 2025 | ("PCSK9 Inhibitors" OR "PCSK9 inhibitor" OR "PCSK9 antibody") OR ("Alirocumab" OR "REGN727" OR "SAR236553" OR "Praluent" OR "Evolocumab" OR "Repatha" OR "AMG145" OR "AMG 145" OR "Inclisiran" OR "Leqvio" OR "ALN-PCSsc" OR "ALN PCSSC" OR "Olpasiran" OR "AMG 890" OR "Zerlasiran" OR "SLN360" OR "SLN-360" OR "Lepodisiran" OR "LY3819469" OR "Pelacarsen" OR "TQJ230" OR "AKCEA-APO(a)-LRx" OR "IONIS-APO(a)-LRx" OR "Muvalaplin" OR "LY3473329") AND ("Lipoprotein(a)" OR "Lipoprotein a" OR "Lp(a)" OR "Low-Density Lipoprotein" OR "LDL") AND (Clinical Trial OR randomized OR "trial") | 1500 |

# **Supplement 2. Network figures**

## **2.1. Lp(a)**

**
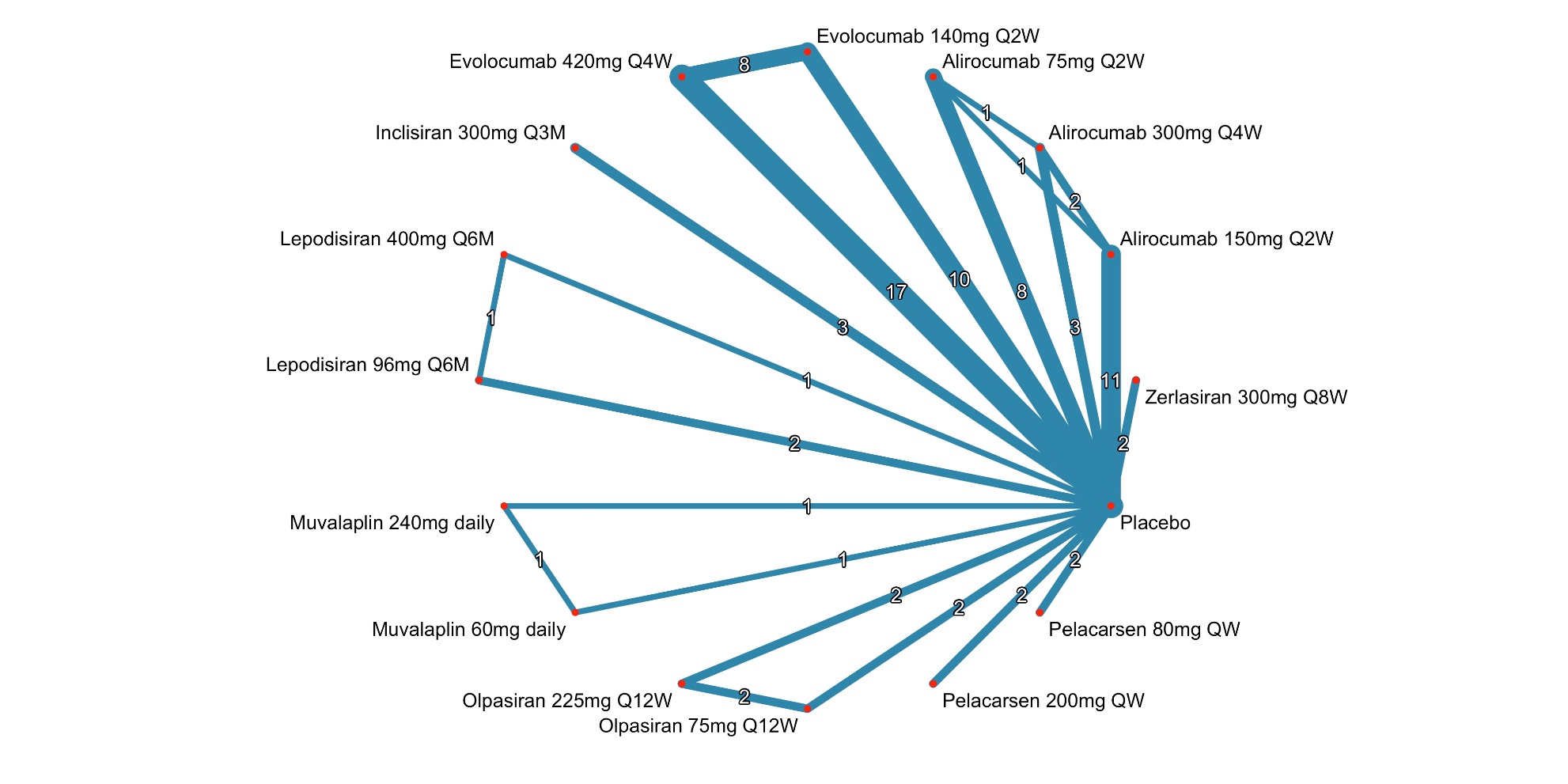
**

## **2.2. Lp(a) - large sample**


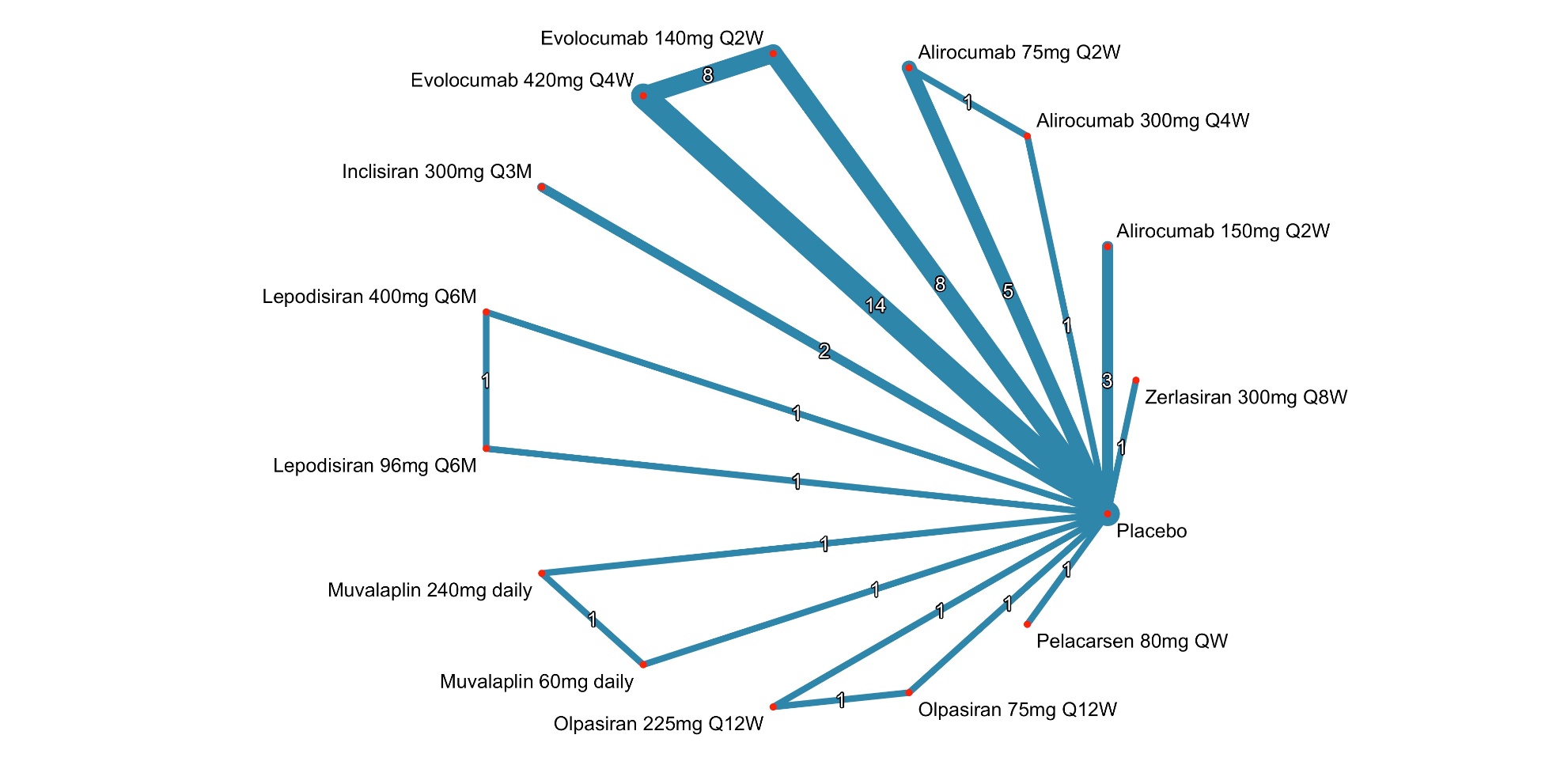


## **2.3. Lp(a) - low risk**

**
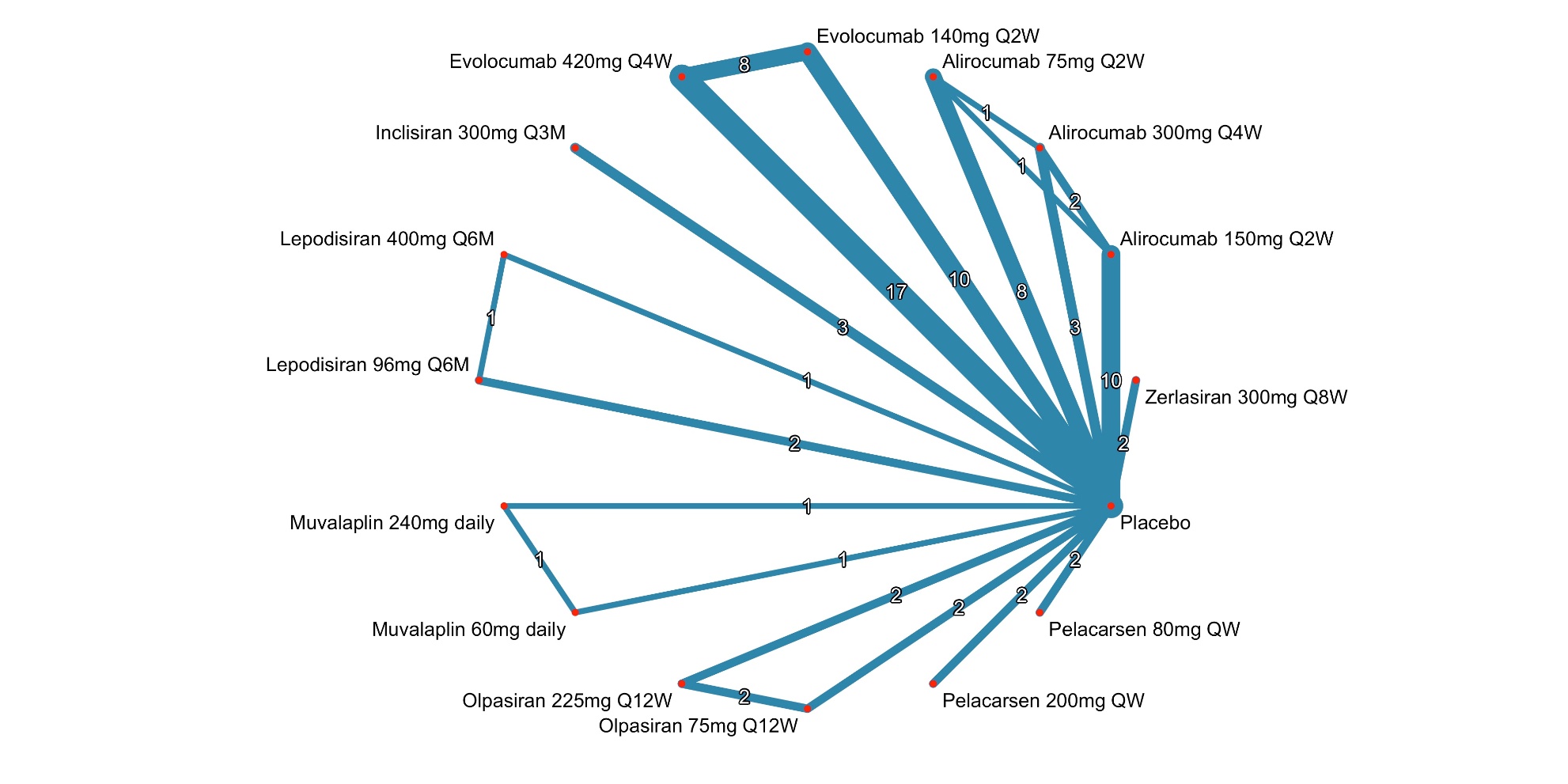
**

## **2.4. LDL-C**

**
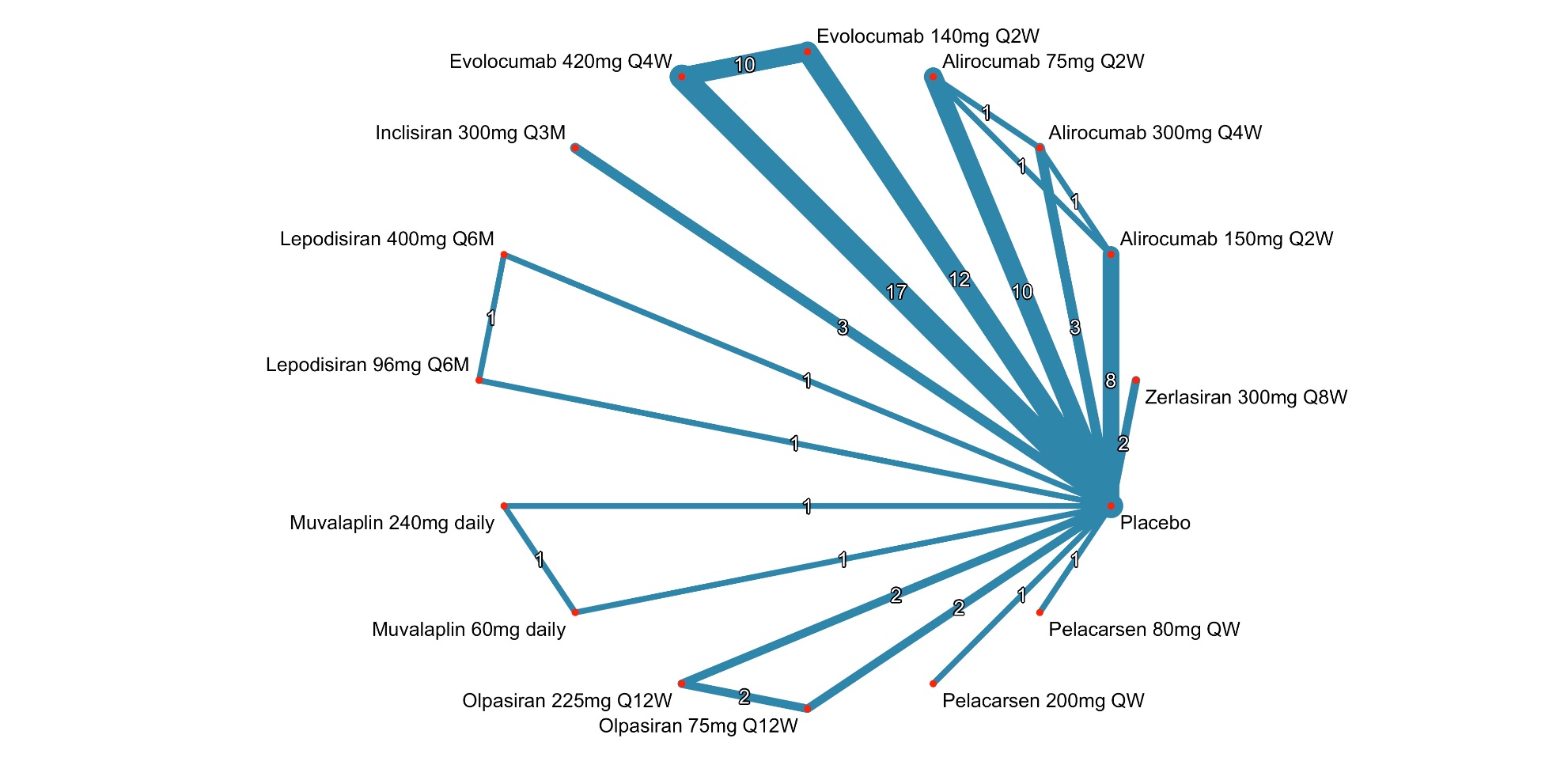
**

## **2.7. TG**

**
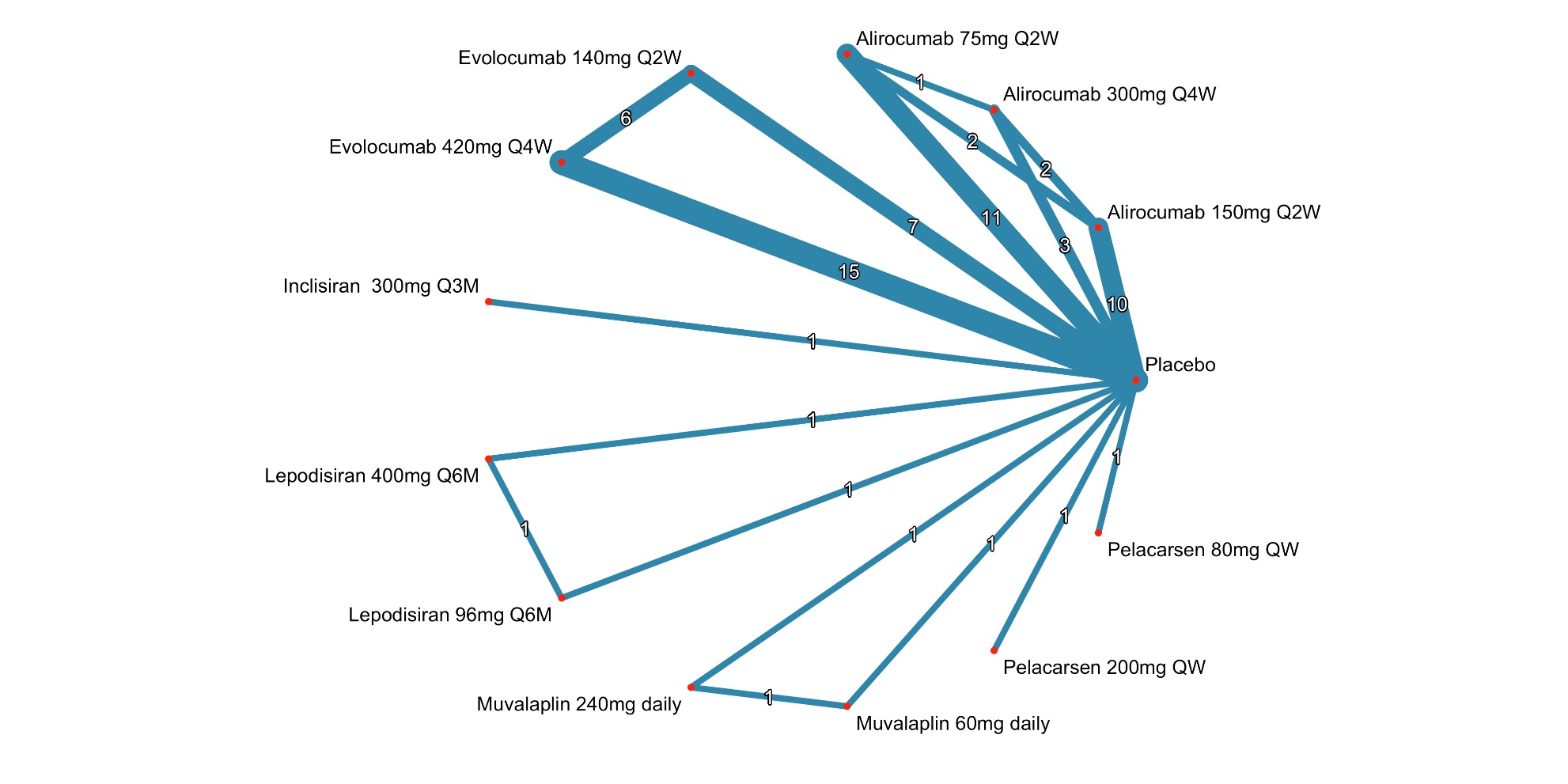
**

## **2.10. Total cholesterol**

**
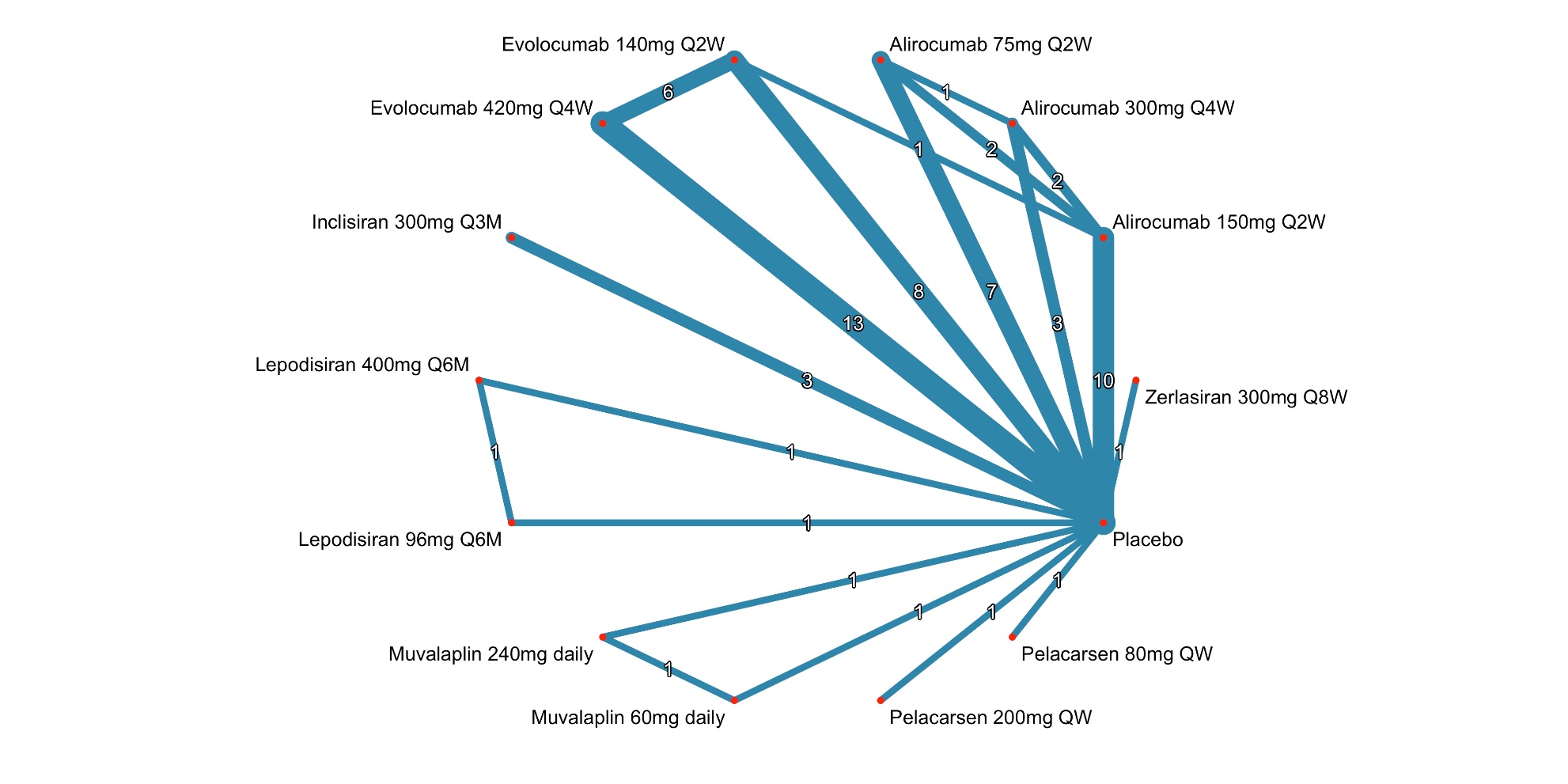
**

## **2.13. HDL**

**
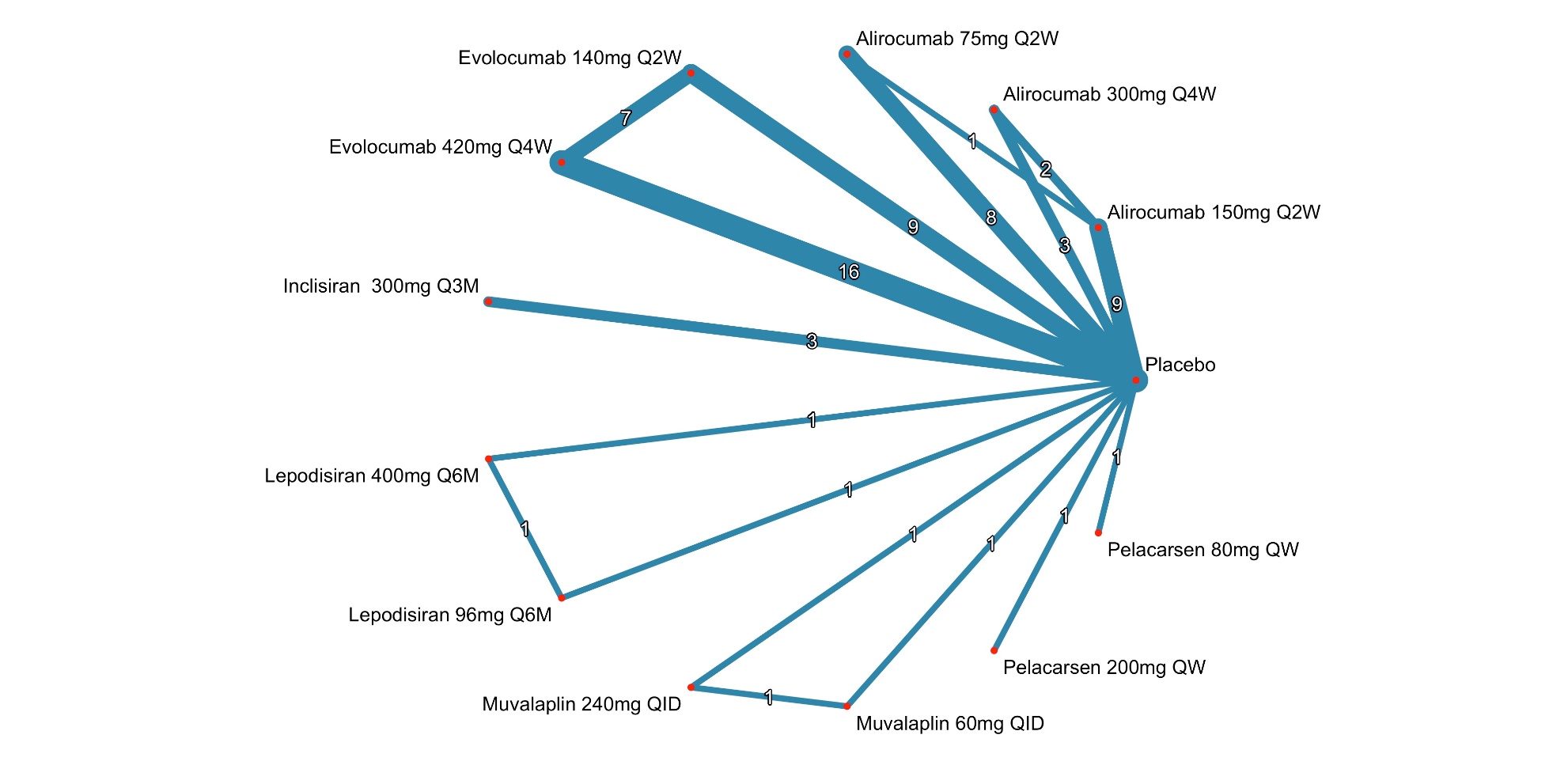
**

## **2.16. Adverse events**

### **2.16.1. Injection site reactions**

**
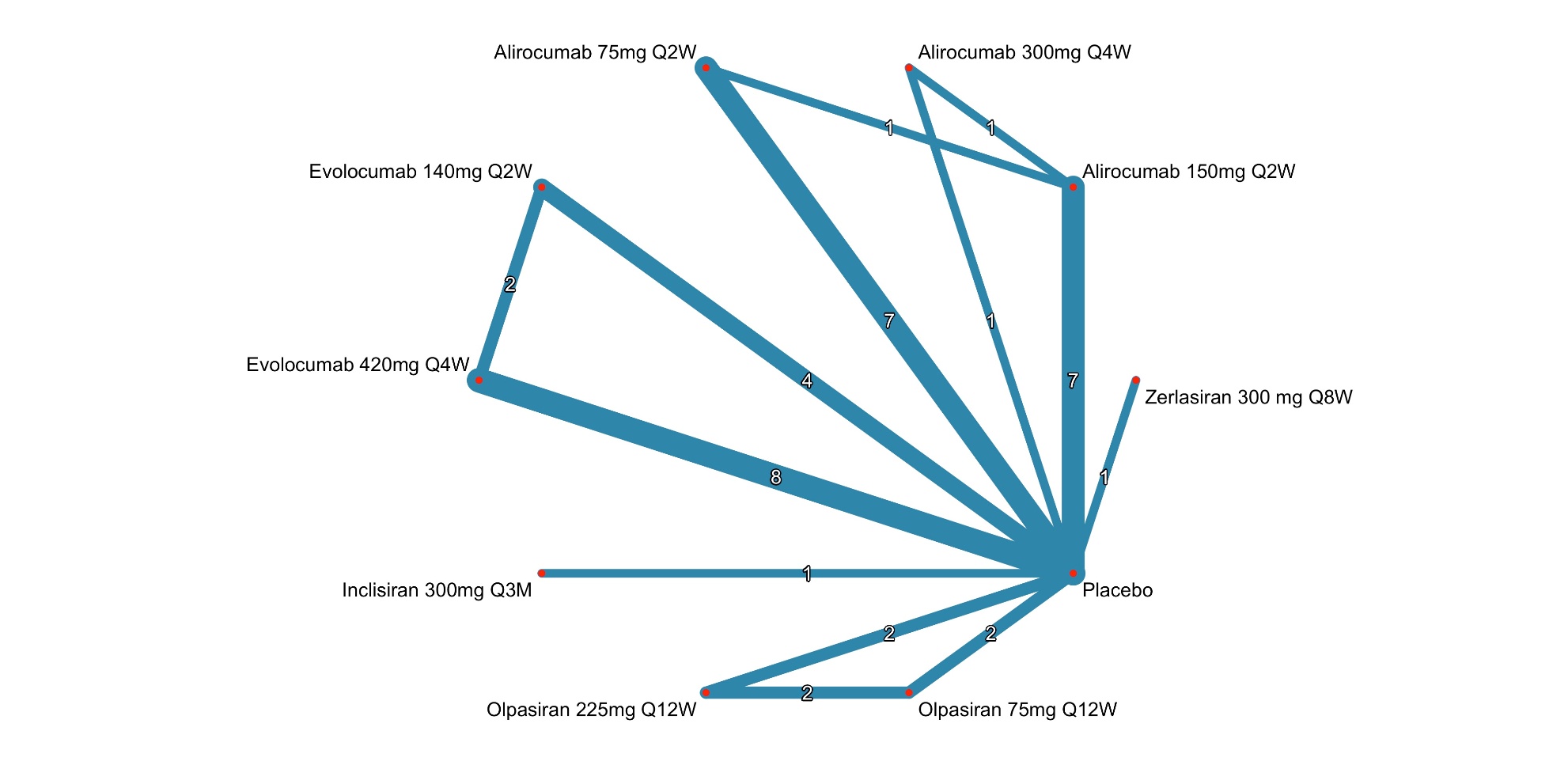
**

### **2.16.2. Injection site reactions - large sample**

**
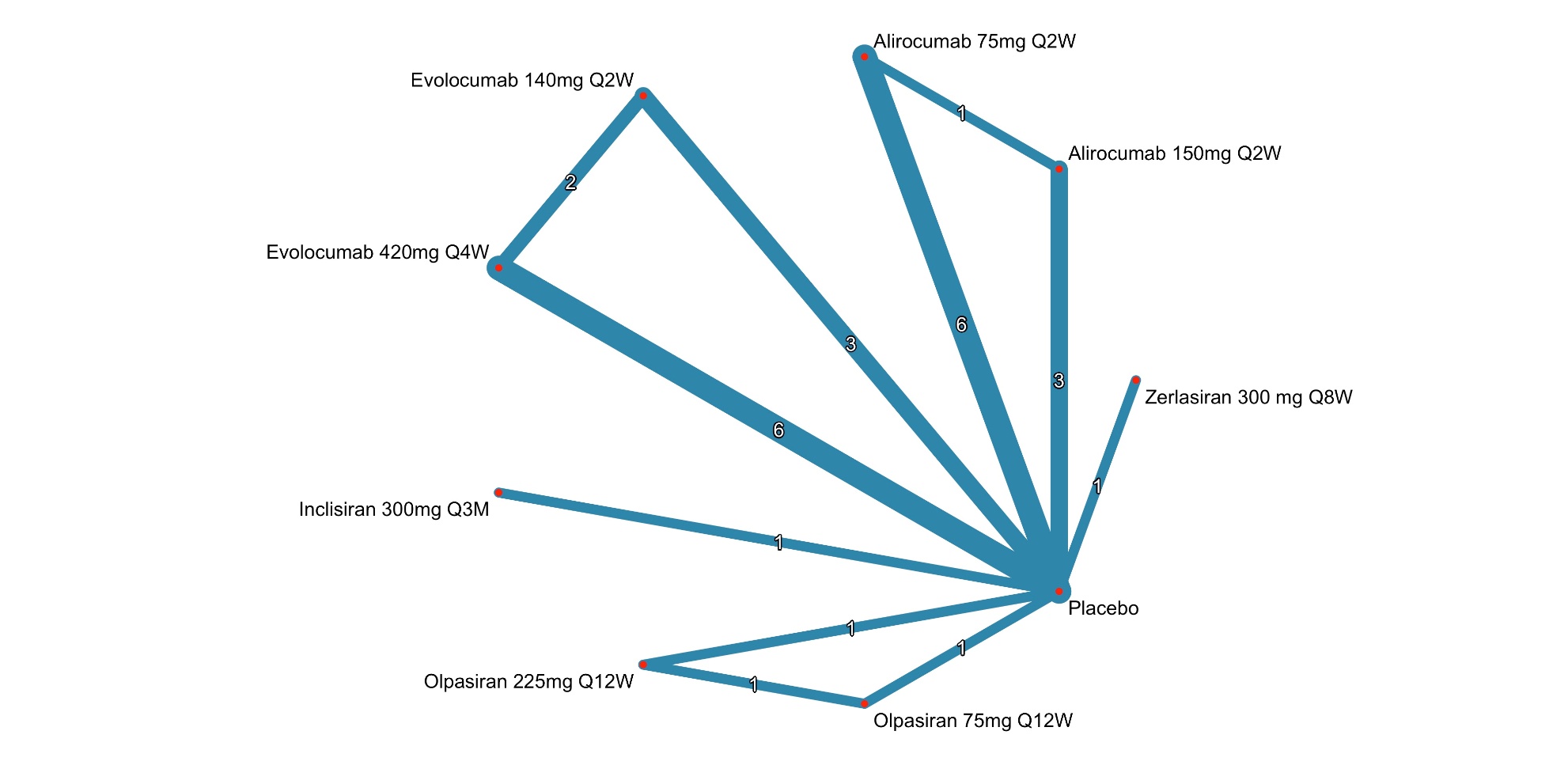
**

### **2.16.3 Injection site reactions - low risk**

**
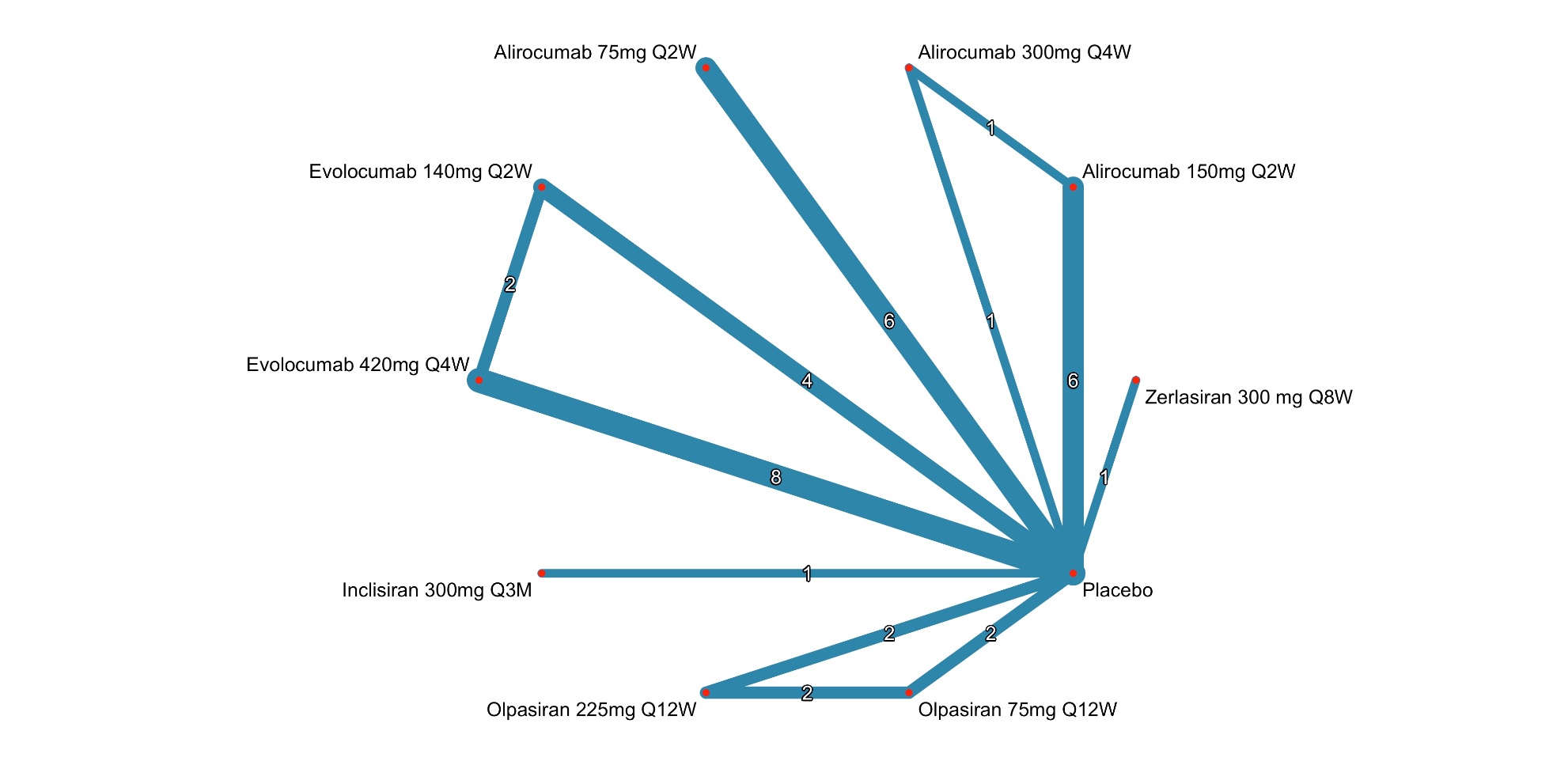
**

### **2.16.4. Serious adverse events**

**
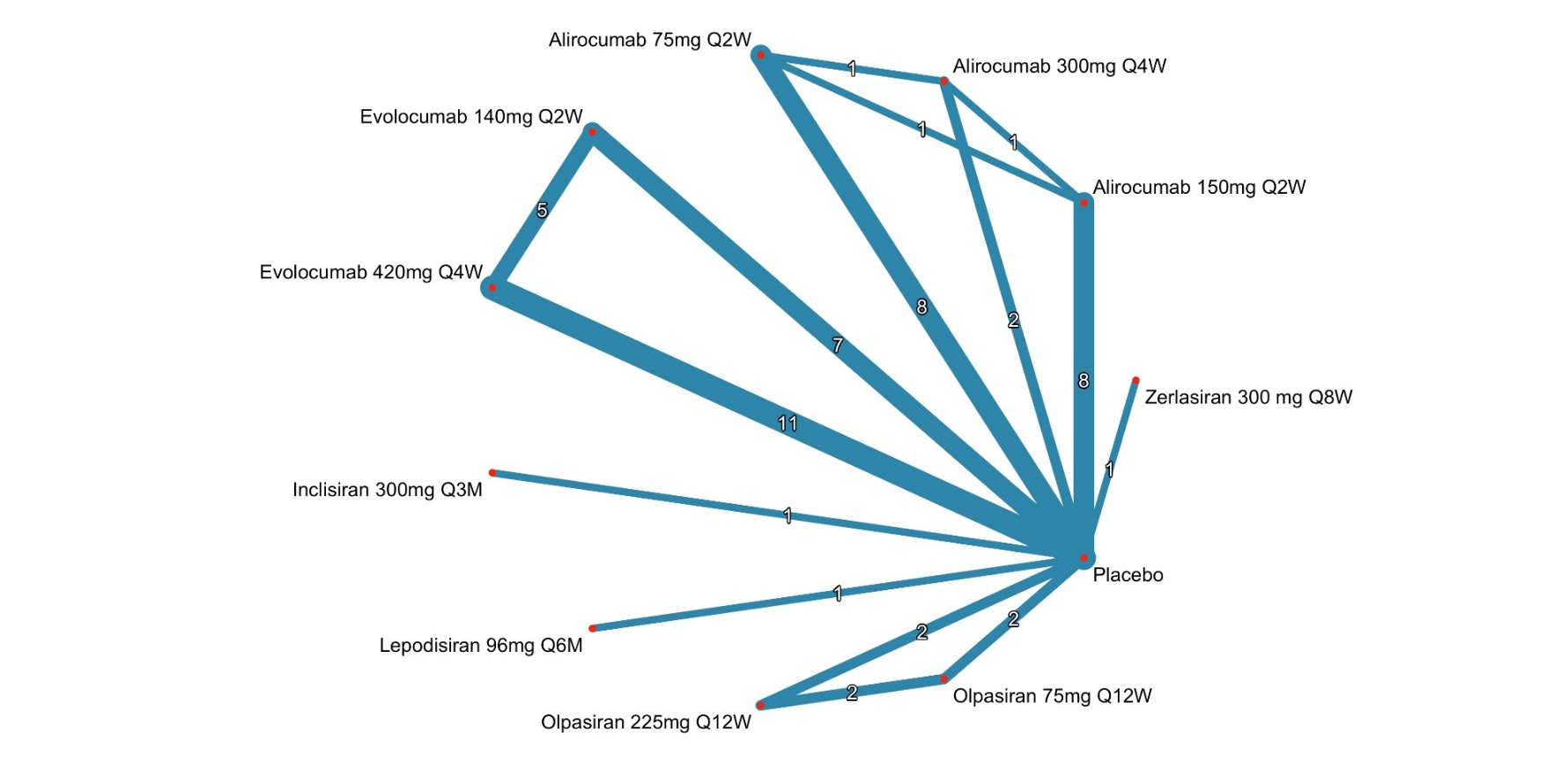
**

### **2.16.5. Serious adverse events - large sample**

**
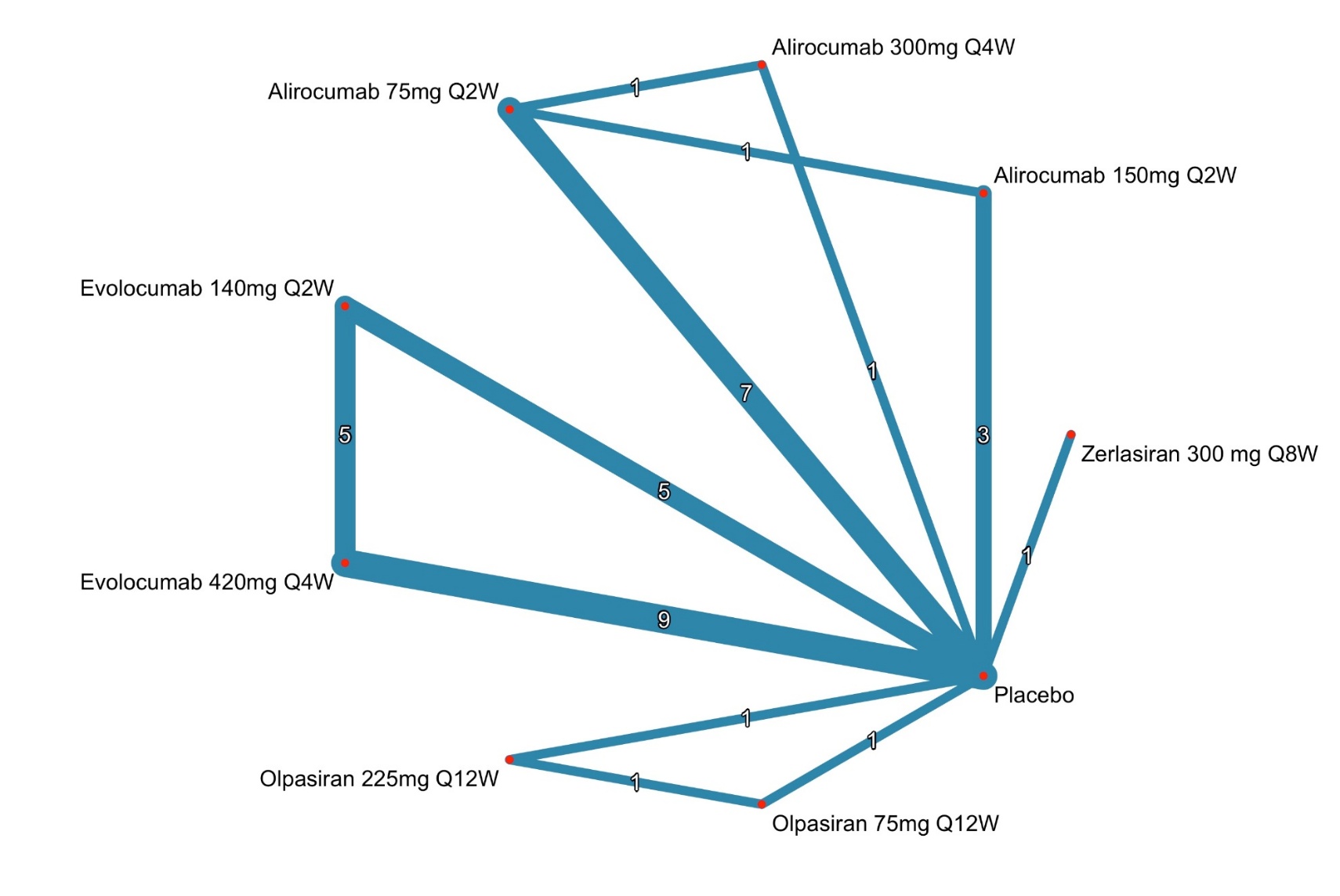
**

### **2.16.6. Serious adverse events - low risk**

**
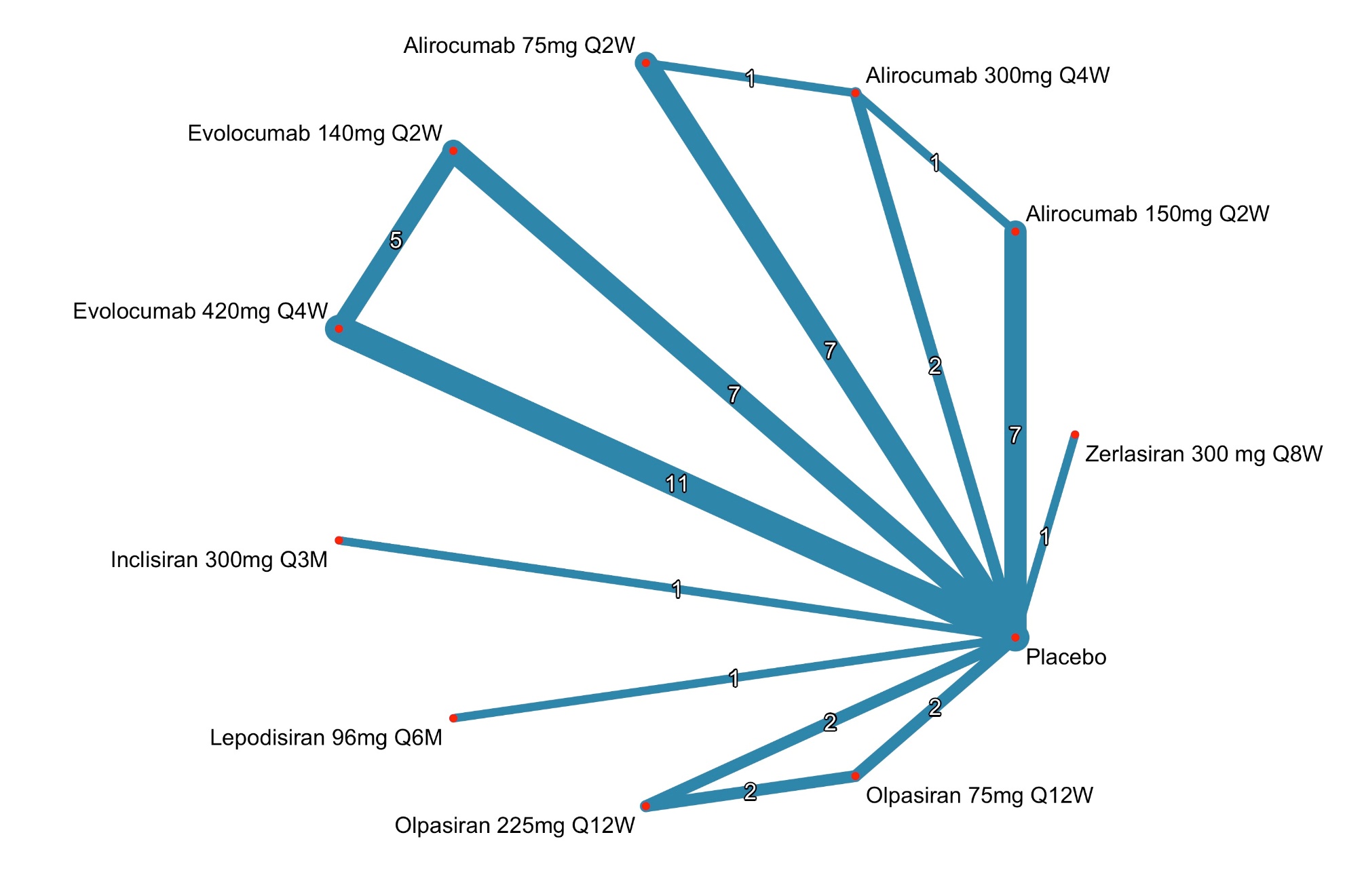
**

### **2.16.7. Treatment discontinuation due to adverse events**

**
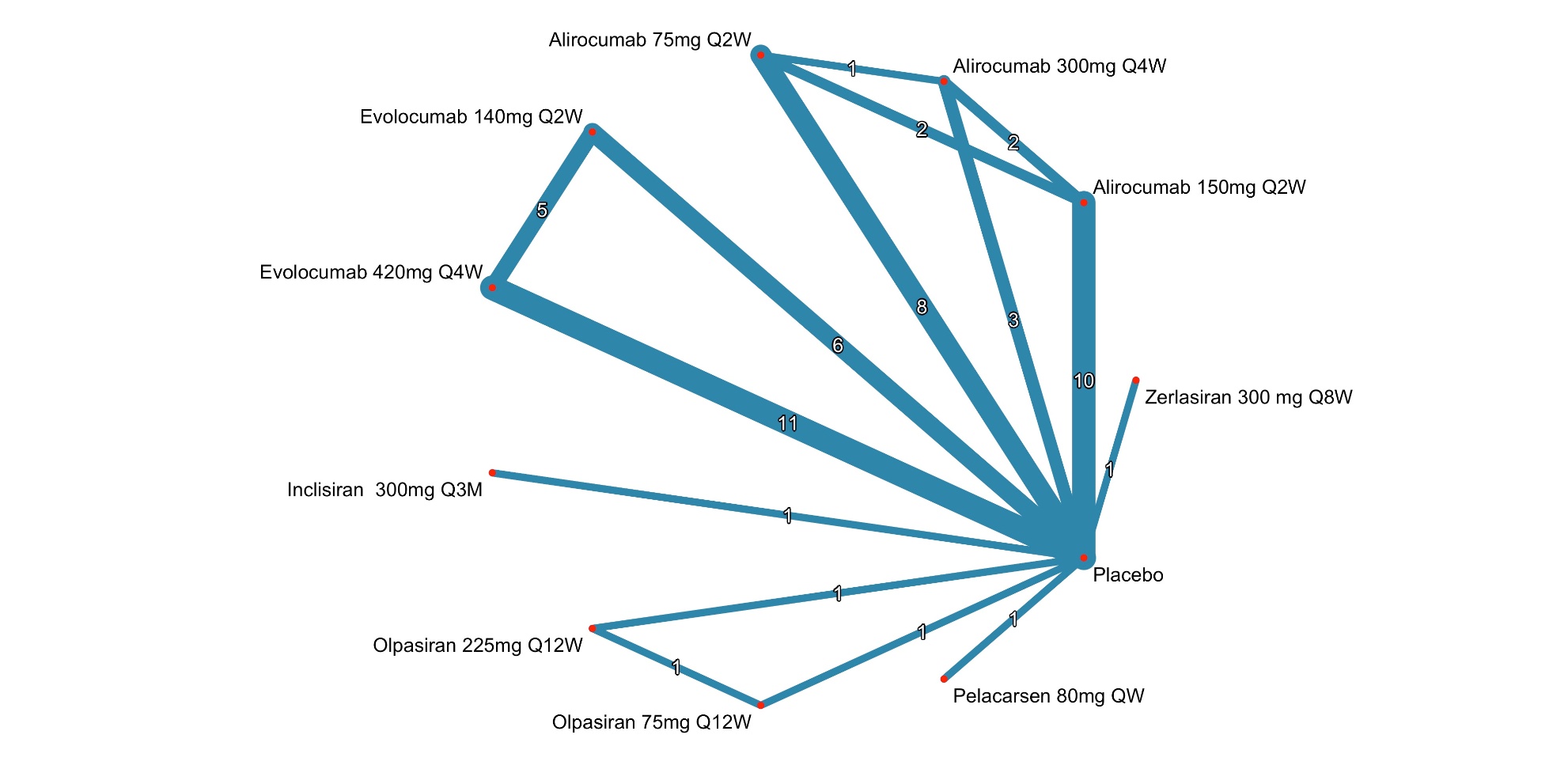
**

### **2.16.8. Treatment discontinuation due to adverse events - large sample**

**
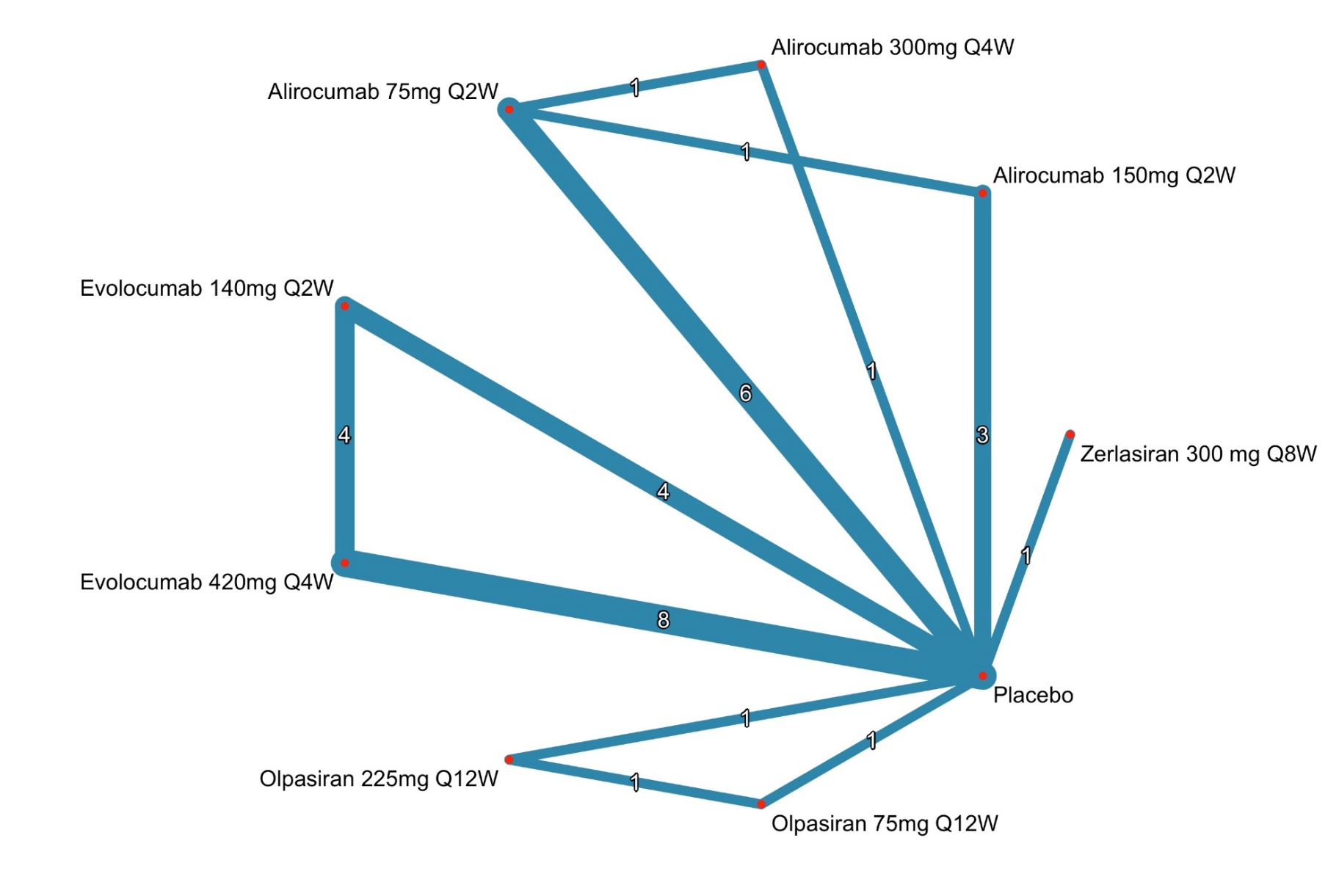
**

### **2.16.9. Treatment discontinuation due to adverse events - low risk**

**
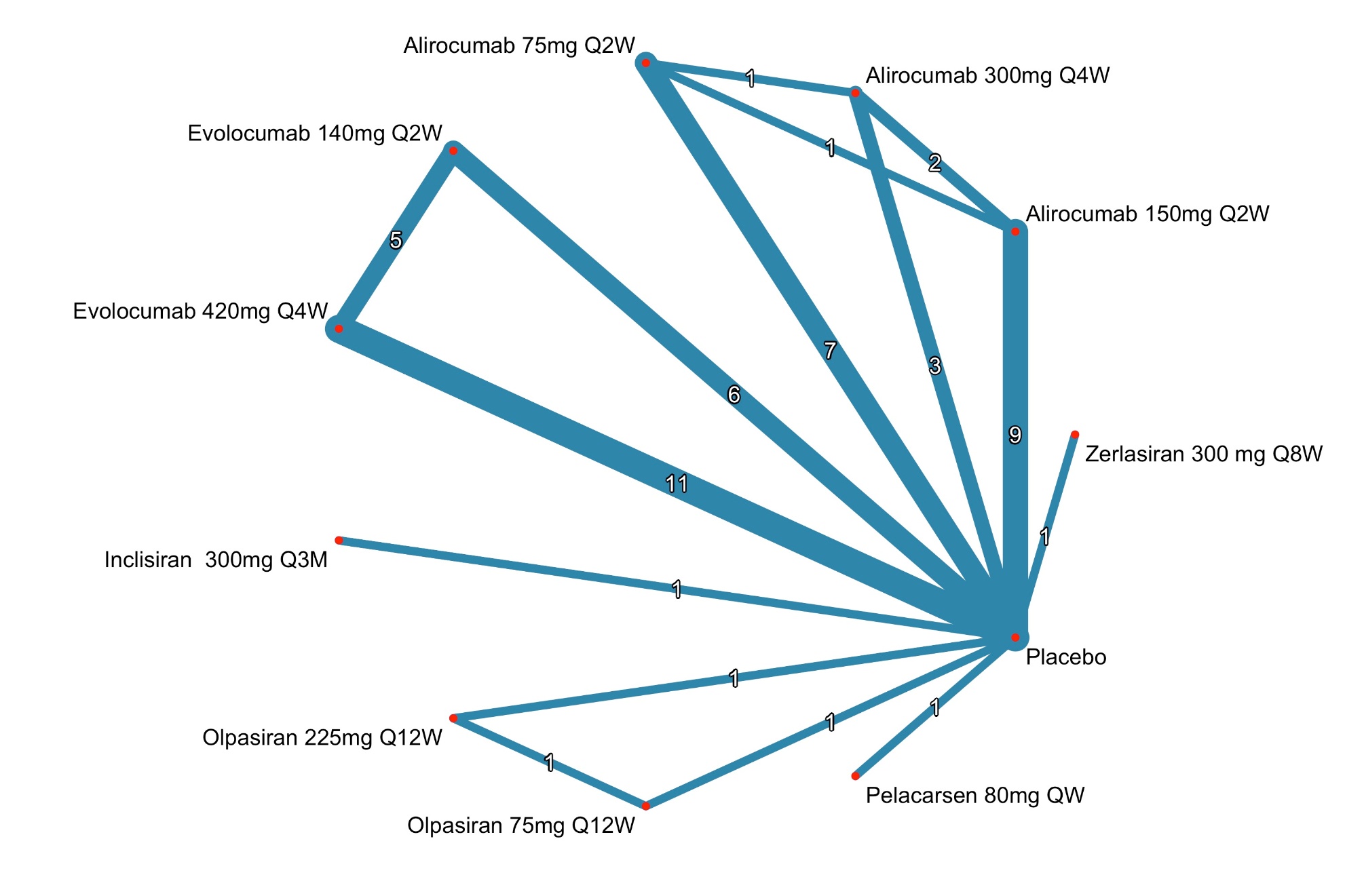
**

# **Supplement 3. Study and Population Characteristics Table**

| **Study ID** | **Year** | **Phase** | **N** | **Population** | **Age (±SD), y** | **Mean Baseline Lp(a) (SD), mg/dL** | **Baseline Lp(a), nmol/L** | **Baseline LDL-C, mg/dL** | **Drug (Dose and Frequency)** | **Duration of Treatment** | **Comparator** | **Primary Outcome** | **Duration (weeks)** |
| --- | --- | --- | --- | --- | --- | --- | --- | --- | --- | --- | --- | --- | --- |
| **Blom,2014** | 2014 | Phase 3 | 901 | Adults with LDL cholesterol >75 mg/dl and a fasting triglyceride <400 mg/dl | 56.2 ± 10.6 | NA | 85.8 ± 102 | 100.3 ± 22.1 | Evolocumab (420mg, QM) | 52 weeks | Placebo, Atorvastatin 10mg, Atorvastatin 80mg, Atorvastatin 80mg+Ezetimibe 10mg | Percent change in LDL-C | 52 |
| **Blom,2020** | 2020 | 3 | 69 | HoFH | 43.38 ± 3.83 | 36.01 ± 40.56 | NA | 282.69 ± 12.72 | Alirocumab (150mg,Q2W) | 12 weeks | Placebo | Percent change in LDL-C | 32 |
| **Boccara,2020** | 2020 | 3 | 464 | People living with human immunodeficiency virus (PLHIV) with hypercholesterolemia/mixed dyslipidemia | 56.4 ± 8.7 | NA | 86.2 ±125 | 133.3 ± 40.1 | Evolocumab (420mg,Q4W)+Atorvastatin 10mg “maximally tolerated statin” | 24 weeks | Placebo+Atorvastatin 10mg “maximally tolerated statin” | Percent change in LDL-C | 24 |
| **Desai,2013** | 2013 | 2 | 626 | Patients with hypercholesterolemia and LDL-C ≥85 mg/dL | 61 ± 9.5 | NA | NA | 123 ± 27.8 | AMG145 (70mg,Q2W) ,(105mg,Q2W) ,(140mg,Q2W) ,(280mg,Q4W) ,(350mg,Q4W) ,(420mg,Q4W) | 12 weeks | Simvastatin 80mg, Atorvastatin ≥40mg, Rosuvastatin ≥20mg, or any statin in combination with ezetimibe. | Percent change in Lp(a) | 12 |
| **Farnier,2016** | 2016 | 3 | 305 | High cardiovascular risk patients | 60.9 ± 10.3 | 38.37 ± 48.34 | NA | 106.8 ± 38.1 | Alirocumab (75mg,Q2W) | 24 weeks | Rosuvastatin (20mg,Q2W) ,(40mg,Q2W) | Percent change in LDL-C | 24 |
| **Gao,2022** | 2022 | Phase 4 | 51 | CAD patients | 61.5 ± 9.1 | NA | 25.9 ± 13.7 | 119.9 ± 34.8 | Alirocumab (75mg,Q2W) | 36 weeks | Atorvastatin 20mg or Rosuvastatin 10mg | Coronary artery calcification (CAC) progression | 36 |
| **Ginsberg,2016** | 2016 | Phase 3 | 107 | HeFH & LDL-C > 160mg/dl | 50.5 ± 13.4 | 26.99 ± 29.47 | NA | 197.8± 53.7 | Alirocumab(150mg, Q2W) + Statin | 78 weeks | Placebo + Statin | Percent change in LDL-C | 78 |
| **Hirayama,2014** | 2014 | Phase 2 | 310 | High CV risk Japanese patients on statins | 61.5 ± 9.7 | NA | 37.89 ± 38.92 | 143.1 ± 19.3 | Evolocumab(70mg,Q2W),(140mg,Q2W),Evolocumab(280mg,QM),(420mg,QM) | 12 weeks | Placebo | Percent change in LDL-C | 12 |
| **Kiyosue,2016** | 2016 | Phase 3 | 404 | hyperlipidemia or mixed dyslipidemia and high cardiovascular risk | 61.5 ± 10.5 | NA | 33.7 ± 31.6 | 105.9 ± 31.7 | Evolocumab (140mg,Q2W),Evolocumab (420mg,QM) | 12 weeks | Placebo+Atrovastatin 5mg, Placebo+Atrovastatin 20mg | Percent change in LDL-C | 12 |
| **Koh,2018** | 2018 | Phase 3 | 199 | High CV risk, hypercholesterolemia | 60.7 ± 9.8 | 30.60 ± 32.77 | NA | 98.18 ± 26.50 | Alirocumab (75mg,Q2W) | 24 weeks | Placebo | Percent change in LDL-C | 24 |
| **Koren,2012** | 2012 | Phase 2 | 406 | Hypercholesterolemia | 50.4 ± 11.7 | NA | 71.2 ± 74.4 | 143.2 ± 22.8 | AMG 145 (70mg Q2W), AMG 145 (105mg,Q2W), AMG 145 (140mg,Q2W), AMG 145 (280mg,Q4W), AMG 145 (350mg,Q4W), AMG 145 (420mg,Q4W) | 12 weeks | Placebo Q2W, Placebo Q4W, Ezetimibe 10mg QD | Percent change in LDL-C | 12 |
| **Koren,2014** | 2014 | Phase 3 | 614 | Hypercholesterolemia | 53 ± 12 | NA | 36.9 ± 54.8 | 142.9 ± 22.8 | Evolocumab (140mg,Q2W),Evolocumab (420mg,QM) | 12 weeks | Placebo (Q2W,QM),Ezetimibe QD | Percent change in LDL-C | 12 |
| **Koren,2022** | 2022 | Phase 1 | 64 | Lp(a) > 70 | 48 | NA | 190.2 ± 57.2 | 113.2 ± 32 | Olpasiran (3mg,single dose), Olpasiran (9mg,single dose), Olpasiran (30mg,single dose), Olpasiran (75mg,single dose), Olpasiran (225 mg,single dose) | 1 dose only | Placebo | Safety and Lp(a) reduction | 32 |
| **Koskinas,2019** | 2019 | 3 | 308 | Patients hospitalized for ACS | 60.8 ± 11.4 | NA | NA | 135.7 ± 37.5 | Evolocumab (420mg,Q4W) | 8 weeks | Placebo+Atorvastatin (40mg,Q4W) | Percent change in LDL-C | 8 |
| **McKenney,2012** | 2012 | Phase 2 | 183 | Primary Hypercholesterolemia | 56.7 ± 10.0 | 35 ± 45 | NA | 127.3 ± 26.4 | Alirocumab (50mg,Q2W), Alirocumab (100mg,Q2W), Alirocumab (150mg,Q2W), Alirocumab (200mg,Q4W), Alirocumab (300mg,Q4W) | 12 weeks | Placebo Q2W | Percent change in LDL-C | 12 |
| **Nicholls,2018** | 2018 | Phase 3 | 968 | ASCVD | 59.8 ± 9.21 | 23.22 ± 36.97 | NA | 92.5 ± 27.2 | Evolocumab(420mg,QM) | 78 weeks | Placebo | Percent Atheroma Volume change | 76 |
| **Nissen,2022** | 2022 | Phase 1 | 32 | Lp(a) >150 nmol/L, no CVD | 50 ± 13.5 | NA | 237.48 ± 109.18 | 107.63 ± 43.73 | Zerlasiran (30mg,single dose), Zerlasiran (100mg,single dose), Zerlasiran (300mg,single dose), Zerlasiran (600mg,single dose) | 21.4 weeks | Placebo | Safety and Lp(a) reduction | 21.4 |
| **Nissen,2024** | 2024 | Phase 2 | 178 | ASCVD | 63.8 ± 9.46 | NA | 222.69 ± 86.25 | 68.2 ± 32 | Zerlasiran(450mg,Q24W), Zerlasiran (300mg,Q16W),Zerlasiran (300 mg,Q24W) | 60 weeks | Placebo | Percent change in Lp(a) | 60 |
|  |  |  | 70 | Patients with cardiovascular disease | 54.8 ± 12.5 | NA | 271.2 ± 122.4 | 77.6 ± 42.8 | Zerlasiran (200mg, Q2W,), Zerlasiran (300 mg, Q8W), Zerlasiran (450mg, Q8W) | 16 weeks | Placebo | Safety and tolerability of Zerlasiran | 52 |
| **O’Donoghue,2022** | 2022 | Phase 2 | 281 | ASCVD, Lp(a) >150 nmol/L | 61.9 ± 9.5 | NA | 272.66 ± 122.50 | 68 ± 24 | Olpasiran (10 mg,Q12W),( 75 mg,Q12W),(225 mg,Q12W),Olpasiran(225 mg,Q24W) | 48 weeks | Placebo | Percent change in Lp(a) | 48 |
| **Raal,2012** | 2012 | Phase 2 | 167 | HeFH on statins ± ezetimibe with LDL-C ≥100 mg/dL | 50.0 ± 13.0 | NA | 69.8 ± 110.2 | 153.4 ± 44.0 | AMG 145 (350mg,Q4W),AMG 145 (420mg,Q4W) | 12 weeks | Placebo | Percent change in LDL-C | 12 |
| **Raal,2015** | 2015 | Phase 3 | 331 | HeFH | 51.1 ± 12.6 | NA | 93.61 ± 125.61 | 155.96 ± 44.04 | Evolocumab(140mg,Q2W),Evolocumab(420mg,QM) | 12 weeks | Placebo | Percent change in LDL-C | 12 |
| **Räber,2022** | 2022 | Phase 3 | 300 | Acute MI | 58.5 ± 9.7 | 32.75 ± 41.15 | NA | 152.4 ± 33.8 | Alirocumab 150mg biweekly + Rosuvastatin 20mg daily | 52 weeks | Placebo | Change in percent atheroma volume | 52 |
| **Ray,2017** | 2017 | Phase 2 | 501 | High Cardiovascular with Elevated LDL Cholesterol | 63.3 ± 11.1 | NA | 63.1 ± 99 | 128.8 ± 52.4 | Inclisiran (200 mg,once), Inclisiran (300mg,once), Inclisiran (500 mg,once), Inclisiran (100mg,Q12W), Inclisiran (200 mg,Q12W), Inclisiran (300 mg,Q12W) | 12.8 weeks | Placebo | Percent change in LDL-C | 25.7 |
| **Ray,2018** | 2018 | 3b/4 | 413 | Patients with T2DM and mixed dyslipidemia | 63.2 ± 9.2 | 23.35 ± 32.99 | NA | 112.7 ± 41.5 | Alirocumab (75mg,Q2W) | 24 weeks | Placebo+Individual maximal tolerated usual care | Percent change in Non-HDL cholesterol | 24 |
| **Robinson,2014** | 2014 | Phase 3 | 1896 | Hypercholesterolemia | 59.9 ± 10.2 | NA | 68.91 ± 111.72 | 109.08 ± 41.13 | (Evolocumab (140mg,Q2W)+different intensities of Statin), (Evolocumab (420mg,QM)+different intensities of Statin) | 12 weeks | Placebo or Ezetimibe | Percent change in LDL-C | 12 |
| **Robinson,2015** | 2015 | Phase 3 | 2300 | IHD,HeFH | 60.5 ± 10.4 | 31.86 ± 44.06 | NA | 122.4 ± 42.2 | Alirocumab (150mg,Q2W) | 24 weeks | Placebo+Statin | Percent change in LDL-C | 24 |
| **Rosenson,2019** | 2019 | 3 | 421 | T2DM patients with hypercholesterolaemia or mixed dyslipidaemia | 62.4 ± 8.5 | NA | 91.8 ± 115.5 | 109.5 ± 14.8 | Evolocumab (420mg,Q4W)+ “maximally tolerated statin per ACC/AHA definitions” | 12 weeks | Placebo+ “Maximally tolerated statin per ACC/AHA definitions” | Percent change in LDL-C | 12 |
| **Rosenson,2024** | 2024 | Phase 4 | 41 | ASCVD+T2DM | 65.4 ± 8.1 | NA | 61.95 ± 73.40 | 102.5 ± 37.5 | Evolocumab 140mg (Q4W, 3 doses) | 12 weeks | Placebo | Percent change in LDL-C | 12 |
| **Roth,2016** | 2016 | Phase 3 | 803 | FH | 60.8 ± 10.1 | 29.3 ± 39.46 | NA | 125.5 ± 33.3 | Alirocumab(75mg,Q2W),(300mg,Q4W) | 48 weeks | Placebo | Percent change in LDL-C | 48 |
| **Rrapo-Kaso,2023** | 2023 | Phase 2 | 35 | PAD | 64.46 ± 8.03 | 81.43 ± 71.70 | NA | 107 ± 36 | Alirocumab 150mg (Q2W) | 52 weeks | Placebo | Plaque Volume change | 52 |
| **Stein,2012_a** | 2012 | Phase 2 | 77 | HeFH | 53.4 ± 9.7 | 55 ± 73 | NA | 155.17 ± 41.63 | Alirocumab (150mg,Q4W), Alirocumab (200mg,Q4W), Alirocumab (300mg,Q4W), Alirocumab (150mg,Q2W) | 12 weeks | Placebo Q2W | Percent change in LDL-C | 12 |
| **Stroes,2016** | 2016 | Phase 3 | 233 | FH | 63.1 ± 10.1 | 20.38 ± 27.69 | NA | 158 ± 16.2 | Alirocumab(75mg,Q2W),(150mg,Q4W) | 24 weeks | Placebo | Percent change in LDL-C | 24 |
| **Tan,2023** | 2023 | Phase 3 | 241 | High/Very High CV Risk | 60.2 ± 10.3 | 87.8 ± 96 | NA | 116.1 ± 34.6 | Evolocumab (140 mg Q2W or 420 mg QM) | 12 weeks | Placebo +Statin | Percent change in LDL-C | 12 |
| **Teramoto,2019** | 2019 | 3 | 163 | Japanese patients with LDL-C ≥100 mg/dL | 63.6 ± 10 | 20.12 ± 18.89 | NA | 150.9 ± 42.8 | Alirocumab (150mg,Q2W),(150mg,Q4W) | 12 weeks | Placebo+Atorvastatin 5mg or non-statin LLT | Percent change in LDL-C | 12 |
| **Yamashita,2024** | 2024 | Phase 2 | 312 | Japanese patients with hypercholesterolemia including HeFH | 63.6 ± 10.5 | NA | NA | 114.0 ± 35.72 | Inclisiran sodium (100mg,3 doses), Inclisiran sodium (200mg,3 doses), Inclisiran sodium (300mg,3 doses) | 38.5 weeks | Placebo | Percent change in LDL-C | 38.5 |
| **Nissen,2025** | 2025 | Phase 2 | 320 | ASCVD | 62.7 (±9.86) | NA | 259.03 (±89.2) | 83.55 (±38.44) | Lepodisiran (16mg,Q6M),(96mg,Q6M),(400mg,Q6M) | 540 days | Placebo | Lp(a) % change | 77.1 |
| **Nicholls,2025** | 2025 | Phase 2 | 233 | ASCVD,T2DM,FH | 64.53 (±9.94) | NA | NA | NA | Muvalaplin (10mg,QD),(60mg,QD),(240mg,QD) | 12 weeks | Placebo | Lp(a) % change | 12 |
| **Tsimikas,2020** | 2020 | Phase 2 | 286 | ASCVD, elevated Lp(a) | 60.02 (±10.04) | NA | 232.5 (±83.89) | 77.4 (±32.67) | Pelacarsen (20mg,Q4W), (40mg,Q4W), (60mg,Q4W), (20mg,Q2W), (20mg,QW) | 24 weeks | Placebo | % change in Lp(a) from baseline to 6 months | 25-27 |
| **Stiekema,2019** | 2019 | Phase 3b | 129 | ASCVD | 60.25 (±9.21) | NA | 219.4 (±109.1) | 145.14 (±38.9) | Evolocumab (420mg,QM) | 16 weeks | Placebo | % change in arterial wall inflammation (MDS TBR) | 16 |
| **Heidemann,2022** | 2022 | Phase 4 | 28 | familial dysbetalipoproteinemia | 62 (±9) | 14.23 (±21.79) | NA | NA | Evolocumab (140mg,Q2W) | 12 weeks | Placebo | 8-hour post-fat load non-HDL-C AUC change | 12 |
| **Raal,2024** | 2024 | Phase 3 | 56 | HoFH | 42.7 (±12.9) | NA | 90.33 (±105.01) | 315.3 (±134.0) | Inclisiran sodium (300mg,Q3M) | 24 weeks | Placebo | % change in LDL-C at Day 150 | 24 |
| **Teramoto_2016_a** | 2016 | Phase 2 | 100 | Primary hypercholesterolemia on atorvastatin | 57.7 (±10.7) | 16.7 (±18.3) | NA | 121.2 (±17.8) | Alirocumab (50,Q2W), (75,Q2W), (150mg,Q2W) | 12 weeks | Placebo | % change in LDL-C from baseline to week 12 | 12 |
| **Teramoto_2016_b** | 2016 | Phase 3 | 216 | heFH or high CV risk (CAD/JAS Cat III), T2DM | 60.8 (±9.5) | 20.28 (±19.23) | NA | 143.1 (±27.1) | Alirocumab (75mg,Q2W) (escalate to 150mg) | 52 weeks | Placebo | % change in LDL-C from baseline to week 24 (ITT) | 52 |
| **Stein_2012_b** | 2012 | Phase 1 | 61 | FH with Atorvastatin,non-FH with Atorvastatin,non-FH Diet Only | 48.2 | NA | NA | 129.5 (±26.9) | Alirocumab (50,Q2W), (100,Q2W), (150mg,Q2W) | 8 weeks | Placebo | Incidence of Adverse Events | 8 |
| **Viney,2016** | 2016 | Phase 2 | 61 | Elevated Lp(a) of 125-437 nmol/L, Elevated Lp(a) ≥ 438 nmol/L | 54.5 (±8.7), 61.3 (±8.0) | NA | 252.9 (±81.64), 456.7 (±97) | 125.78 (±43.6), 105.1 (±35.2) | Pelacarsen (200mg,QW) | 12 weeks | Placebo | % change in Lp(a) | 12 |
| **Tsimikas,2015** | 2015 | Phase 1 | 31 | Healthy, Lp(a) more than 25 nmol/L | 39 (±10.6) | NA | 105 (±74.6) | 112 (±28.6) | Pelacarsen (100mg) (200mg) (300mg) | 4 weeks | Placebo | % change in Lp(a) | 15.1 |
| **Karwatowska-Prokopczuk,2023** | 2023 | Phase 1 | 9 | Healthy | 46.9 (±8.1) | NA | Single dose: 57.3 (±49.7) Multiple dose: 81.1 (±137.5) | 128 (±39.6) | Pelacarsen(80mg,Q4W), (20mg,Single dose), (40mg,Single dose), (80mg,Single dose) | 85 days | Placebo | Safety | 12 |
| **Nicholls,2022** | 2022 | Phase 3 | 161 | ACS | 60.5 (±9.61) | NA | 101.9 (±105.6) | 141.3 (±33.2) | Evolocumab (420mg,Q4W) | 50 weeks | Placebo | Fibrous cap thickness | 50 |
| **Nissen,2023** | 2023 | Phase 1 | 48 | Healthy | 46.8 (±11.6) | NA | 115.3 (±52.7) | 132 (±33) | Lepodisiran (4mg, single dose) , (12mg, single dose) , (32mg, single dose) , (96mg, single dose) , (304mg, single dose) , (608mg, single dose) | 48 weeks | Placebo | Safety of lepodisiran | 48 |
| **Kereiakes,2015** | 2015 | Phase 3 | 316 | ASCVD,FH | 63 (±19.4) | 39.3 (±51.5) | NA | 102.2 (±66.1) | Alirocumab(75mg,Q2W) | 12 weeks | Placebo | LDL-C | 52 |
| **Lorenzatti,2019** | 2019 | Phase 3 | 981 | T2DM and dyslipidemia | 59.2 (±6.03) | NA | 69.4 | 92.8 | Evolocumab(420mg, Q4W)+Evolocumab(120mg,Q2W) | 12 weeks | Placebo | LDL-C | 12 |

# **Supplement 4. Risk of Bias Assessment**

| **Unique ID** | **Study ID** | **D1** | **D2** | **D3** | **D4** | **D5** | **Overall** |
| --- | --- | --- | --- | --- | --- | --- | --- |

| 1 | Blom,2014 | \|  \| \| --- \| | \|  \| \| --- \| | \|  \| \| --- \| | \|  \| \| --- \| | \|  \| \| --- \| | \|  \| \| --- \| |
| --- | --- | --- | --- | --- | --- | --- | --- | --- | --- | --- | --- | --- | --- |
| 2 | Blom,2020 |  |  |  |  |  |  |
| 3 | Boccara,2020 |  |  |  |  |  |  |
| 4 | Desai,2013 |  |  |  |  |  |  |
| 5 | Farnier,2016 |  |  |  |  |  |  |
| 6 | Gao,2022 |  |  |  |  |  |  |
| 7 | Ginsberg,2016 |  |  |  |  |  |  |
| 8 | Heidemann,2022 |  |  |  |  |  |  |
| 9 | Hirayama,2014 |  |  |  |  |  |  |
| 10 | Karwatowska-Prokopczuk,2023 |  |  |  |  |  |  |
| 11 | Kereiakes,2015 |  |  |  |  |  |  |
| 12 | Kiyosue,2016 |  |  |  |  |  |  |
| 13 | Koh,2018 |  |  |  |  |  |  |
| 14 | Koren,2012 |  |  |  |  |  |  |
| 15 | Koren,2014 |  |  |  |  |  |  |
| 16 | Koren,2022 |  |  |  |  |  |  |
| 17 | Koskinas,2019 |  |  |  |  |  |  |
| 18 | Lorenzatti,2019 |  |  |  |  |  |  |
| 19 | McKenney,2012 |  |  |  |  |  |  |
| 20 | Nicholls,2018 |  |  |  |  |  |  |
| 21 | Nicholls,2022 |  |  |  |  |  |  |
| 22 | Nicholls,2025 |  |  |  |  |  |  |
| 23 | Nissen,2022 |  |  |  |  |  |  |
| 24 | Nissen,2023 |  |  |  |  |  |  |
| 25 | Nissen,2024 |  |  |  |  |  |  |
| 26 | Nissen,2025 |  |  |  |  |  |  |
| 27 | O’Donoghue,2022 |  |  |  |  |  |  |
| 28 | Raal,2012 |  |  |  |  |  |  |
| 29 | Raal,2015 |  |  |  |  |  |  |
| 30 | Raal,2024 |  |  |  |  |  |  |
| 31 | Räber,2022 |  |  |  |  |  |  |
| 32 | Ray,2017 |  |  |  |  |  |  |
| 33 | Ray,2018 |  |  |  |  |  |  |
| 34 | Robinson,2014 |  |  |  |  |  |  |
| 35 | Robinson,2015 |  |  |  |  |  |  |
| 36 | Rosenson,2019 |  |  |  |  |  |  |
| 37 | Rosenson,2024 |  |  |  |  |  |  |
| 38 | Roth,2016 |  |  |  |  |  |  |
| 39 | Rrapo-Kaso,2023 |  |  |  |  |  |  |
| 40 | Stein,2012_a |  |  |  |  |  |  |
| 41 | Stein_2012_b |  |  |  |  |  |  |
| 42 | Stiekema,2019 |  |  |  |  |  |  |
| 43 | Stroes,2016 |  |  |  |  |  | **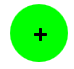** |
| 44 | Tan,2023 |  |  |  |  |  |  |
| 45 | Teramoto,2019 |  |  |  |  |  |  |
| 46 | Teramoto_2016_a |  |  |  |  |  |  |
| 47 | Teramoto_2016_b |  |  |  |  |  |  |
| 48 | Tsimikas,2015 |  |  |  |  |  |  |
| 49 | Tsimikas,2020 |  |  |  |  |  |  |
| 50 | Viney,2016 |  |  |  |  |  |  |
| 51 | Yamashita,2024 |  |  |  |  |  |  |

|  | Low risk |  |
| --- | --- | --- |
|  | Some concerns |  |
|  | High risk |  |
| D1 | Randomization process | |
| D2 | Deviations from the intended interventions | |
| D3 | Missing outcome data | |
| D4 | Measurement of the outcome | |
| D5 | Selection of the reported result | |

# **Supplement 5. Publication bias (funnel plot)**

## **5.1. Lp(a)**

**
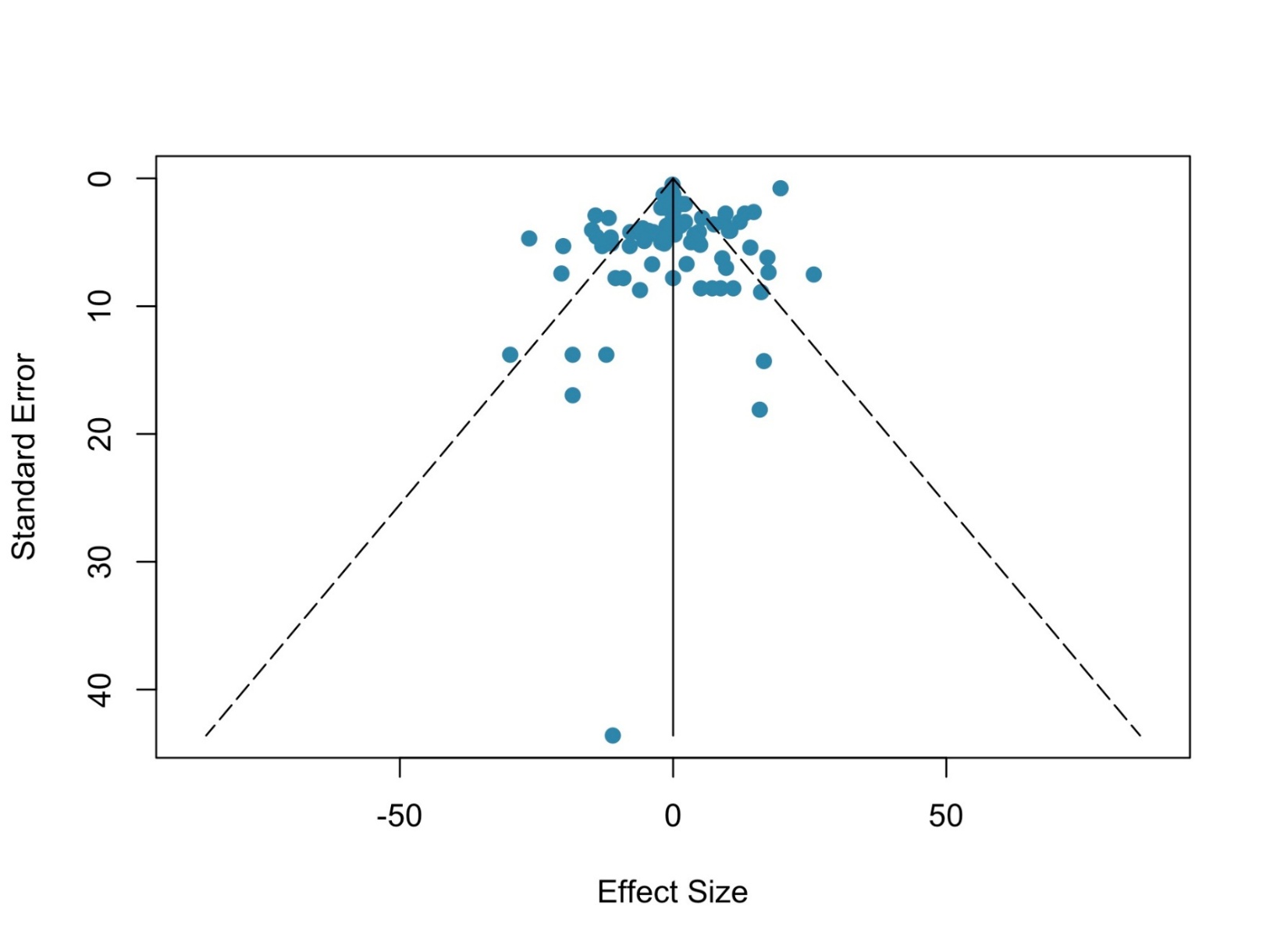
**

## **5.2. Lp(a) fixed**


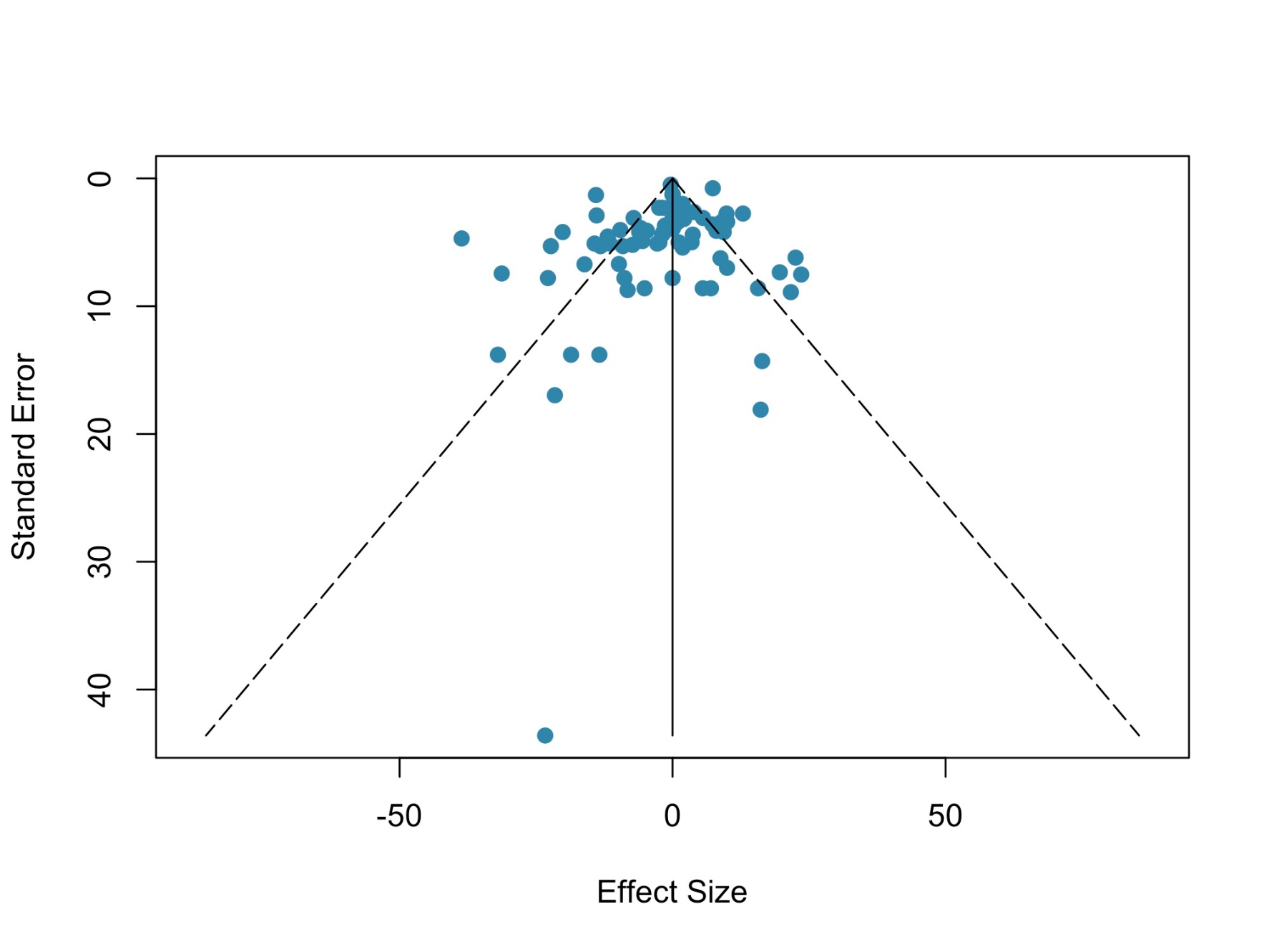


## **5.3. Lp(a) Low risk**


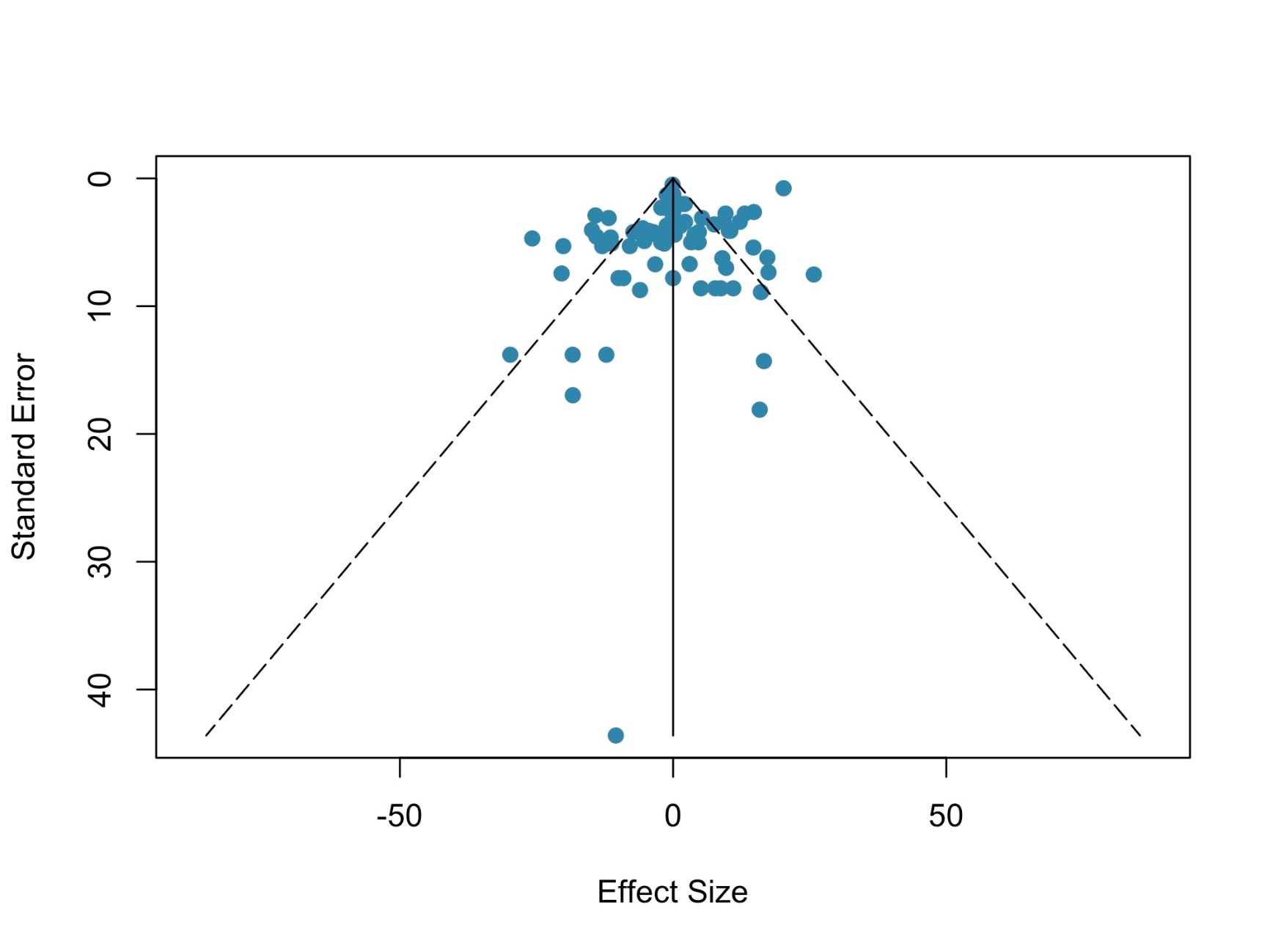


## **5.4. Lp(a) Large sample**


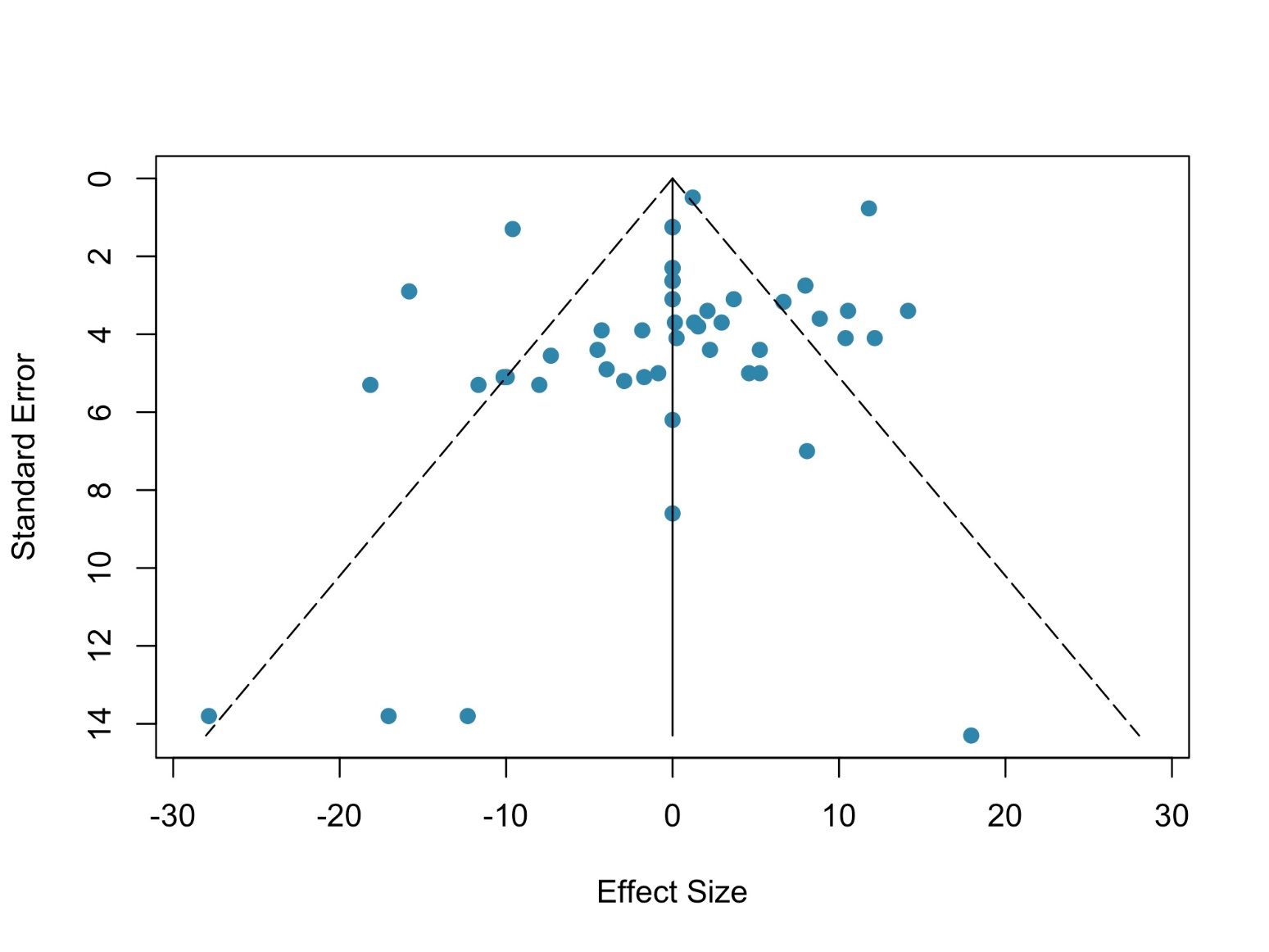


## **5.5. LDL-C**

**
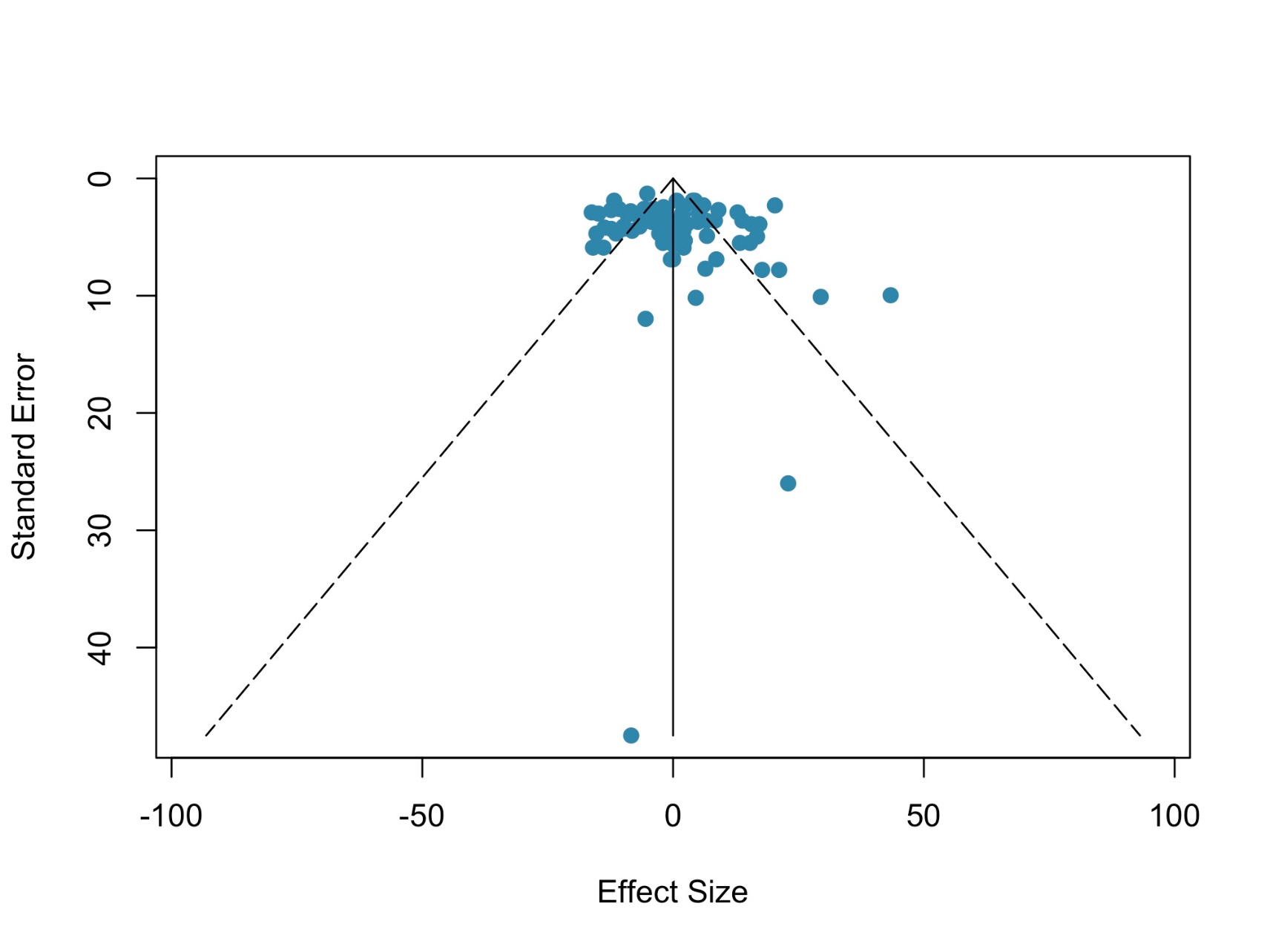
**

## **5.6. LDL-C fixed**


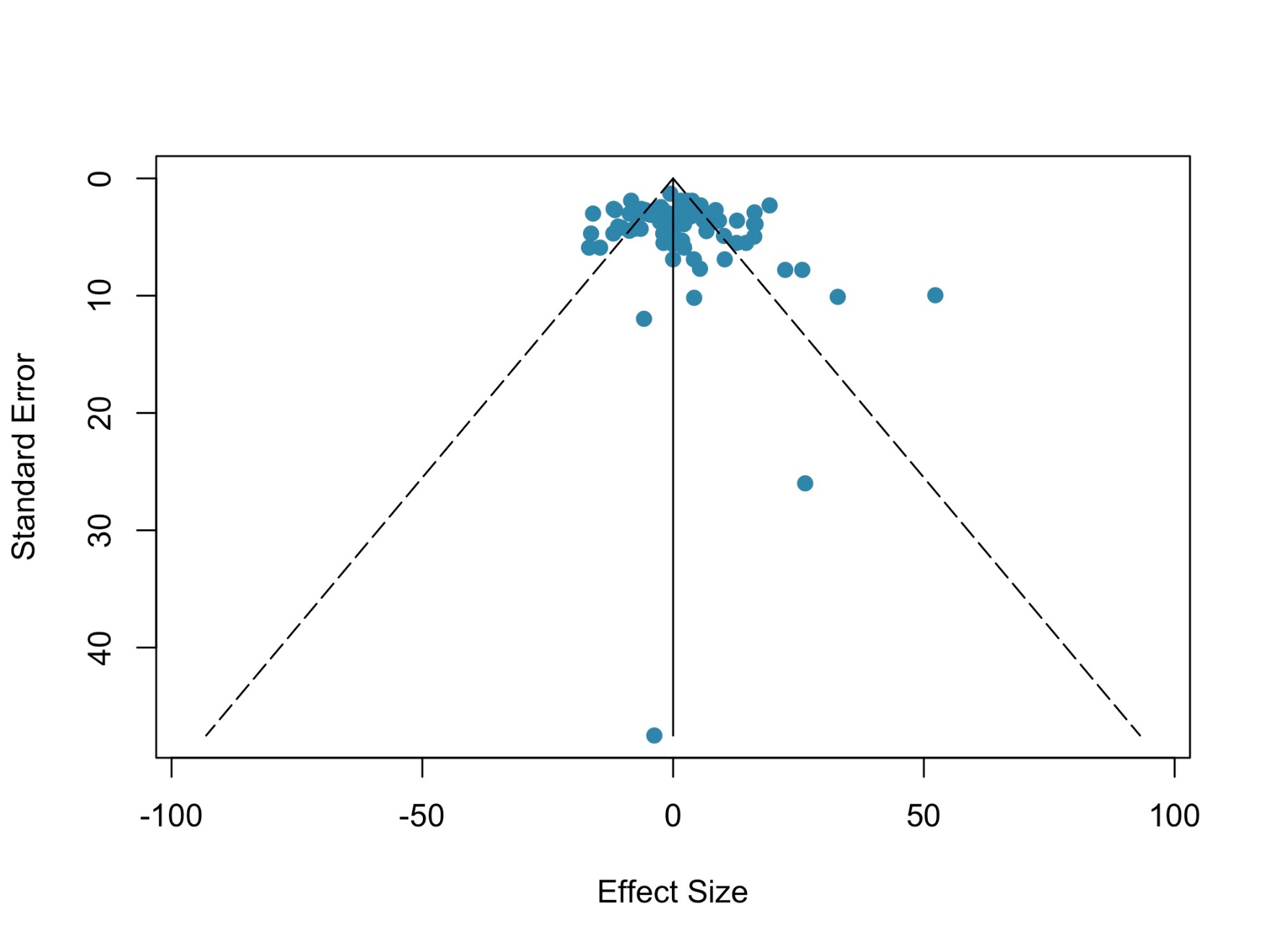


## **5.7. LDL-C Low risk**


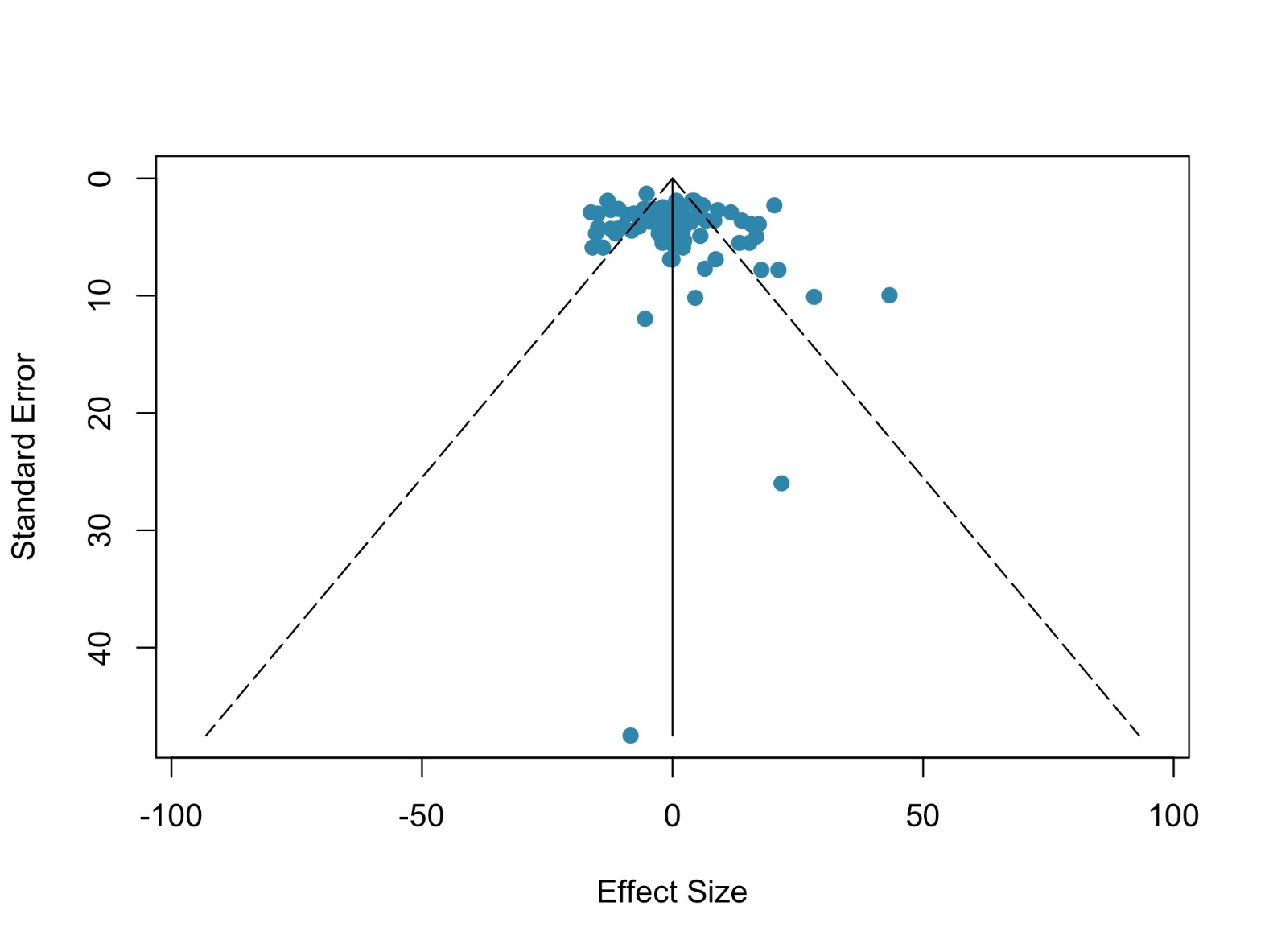


## **5.8. LDL-C Large sample**


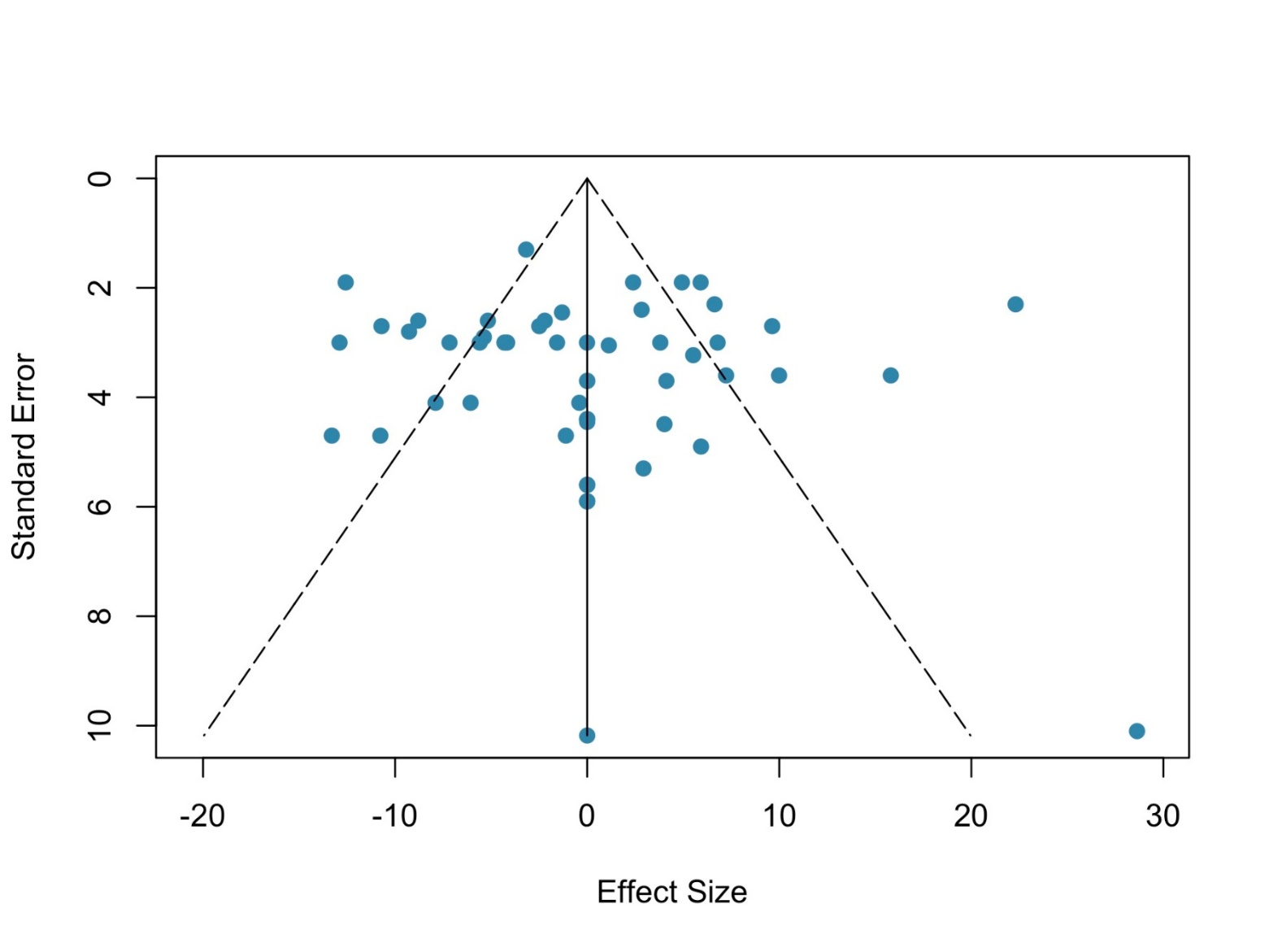


## **5.9. TG**

**
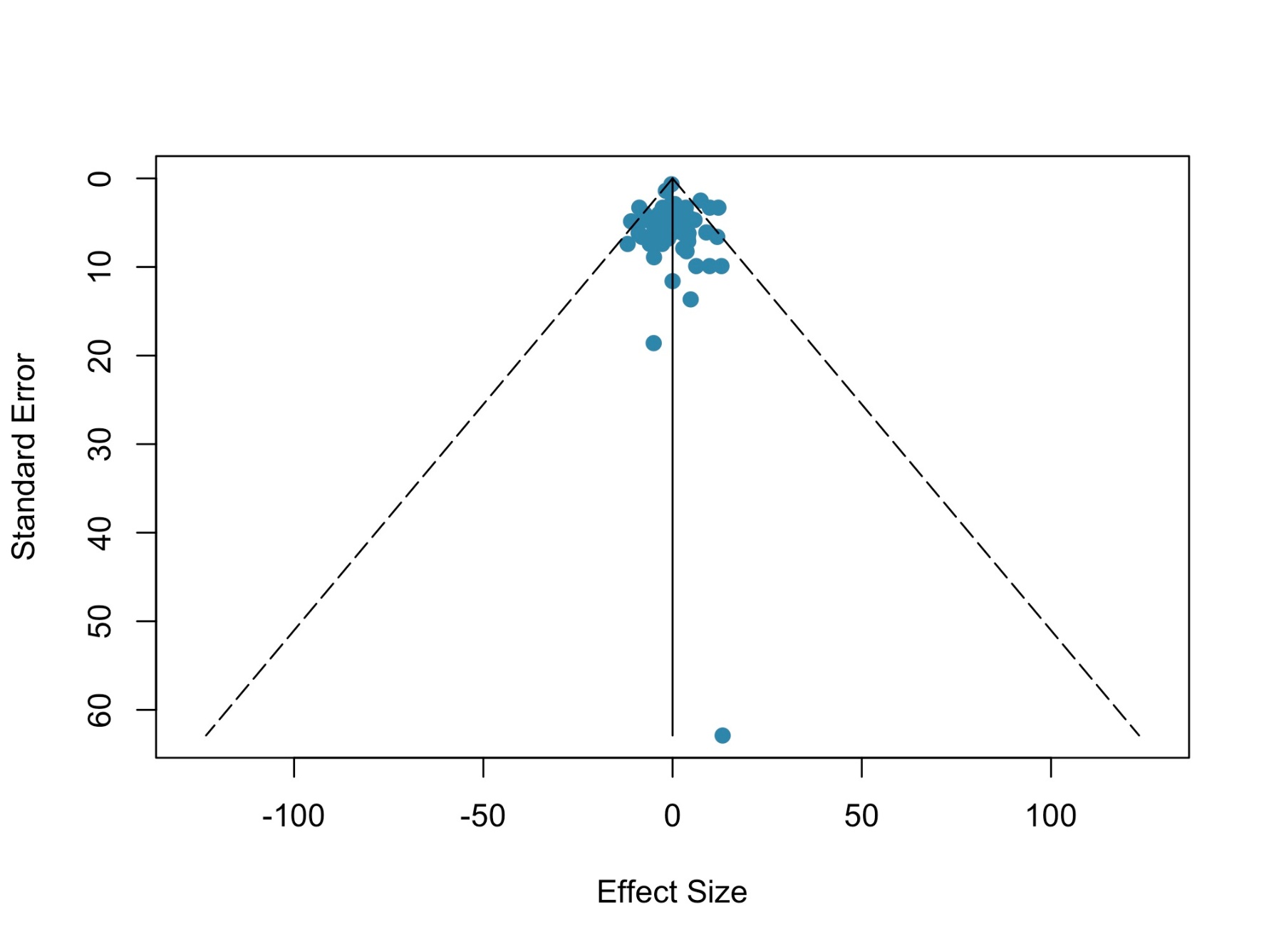
**

## **5.10. TG fixed**


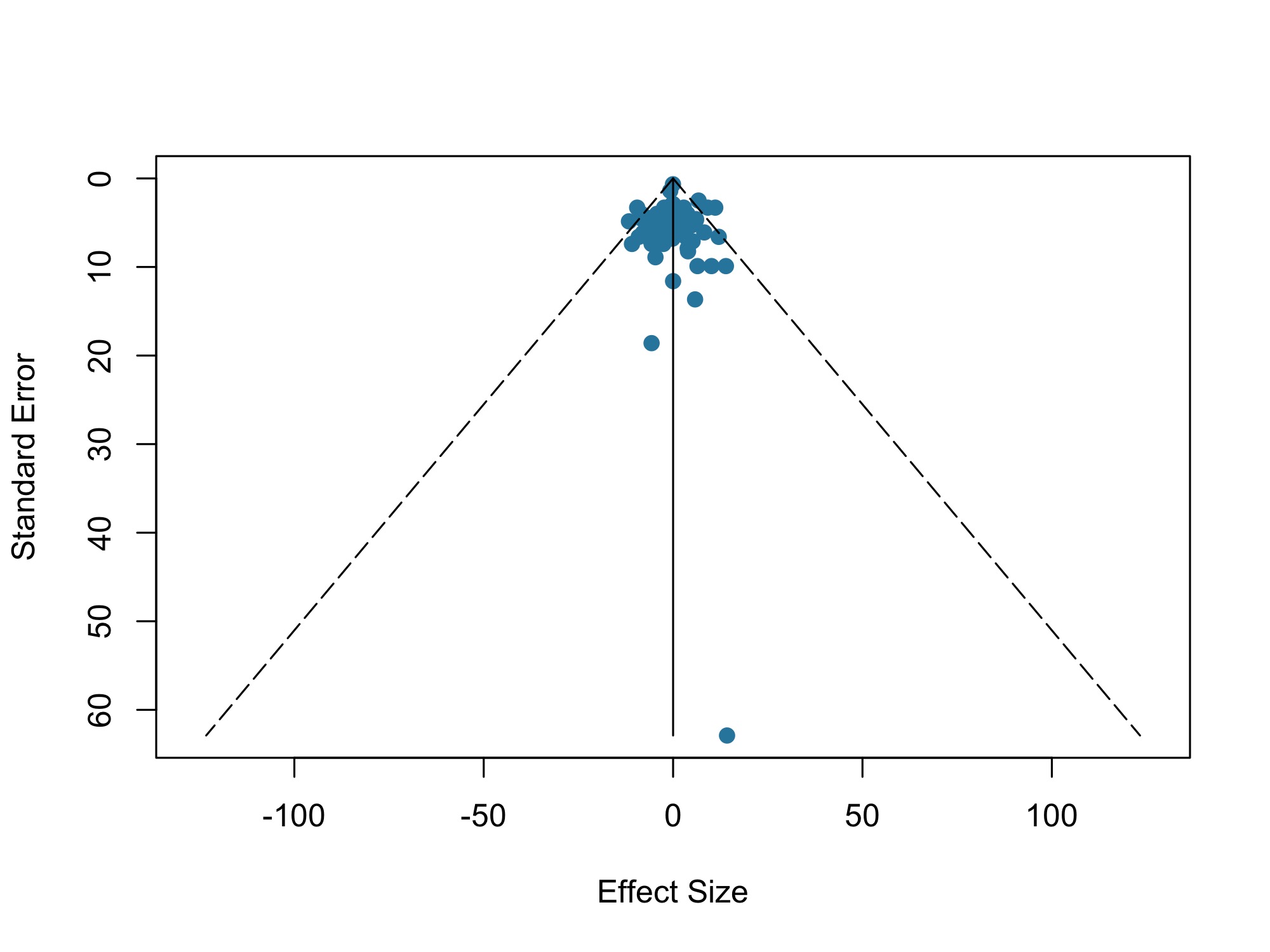


## **5.11. TG Low risk**


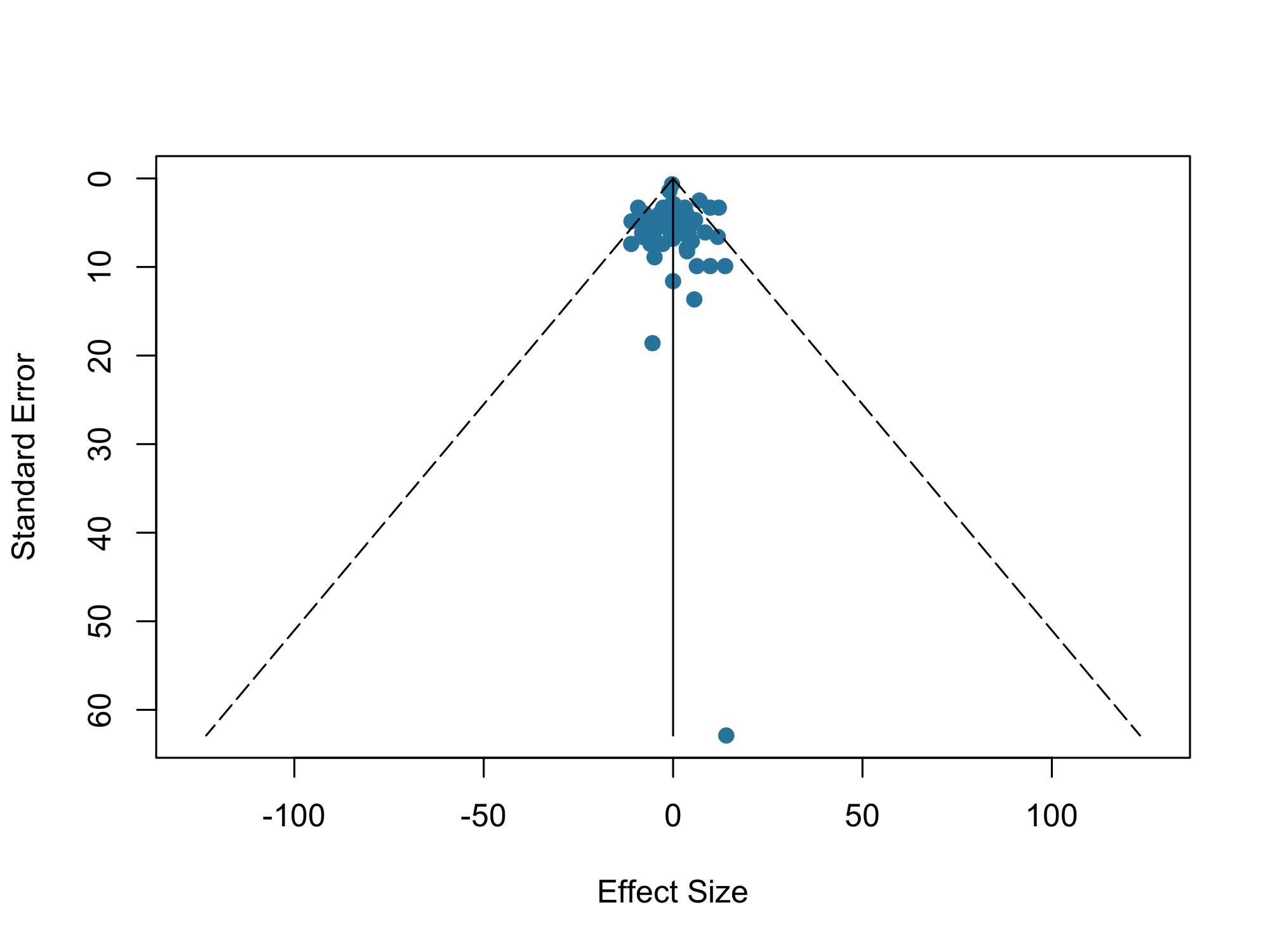


## **5.12. TG Large sample**


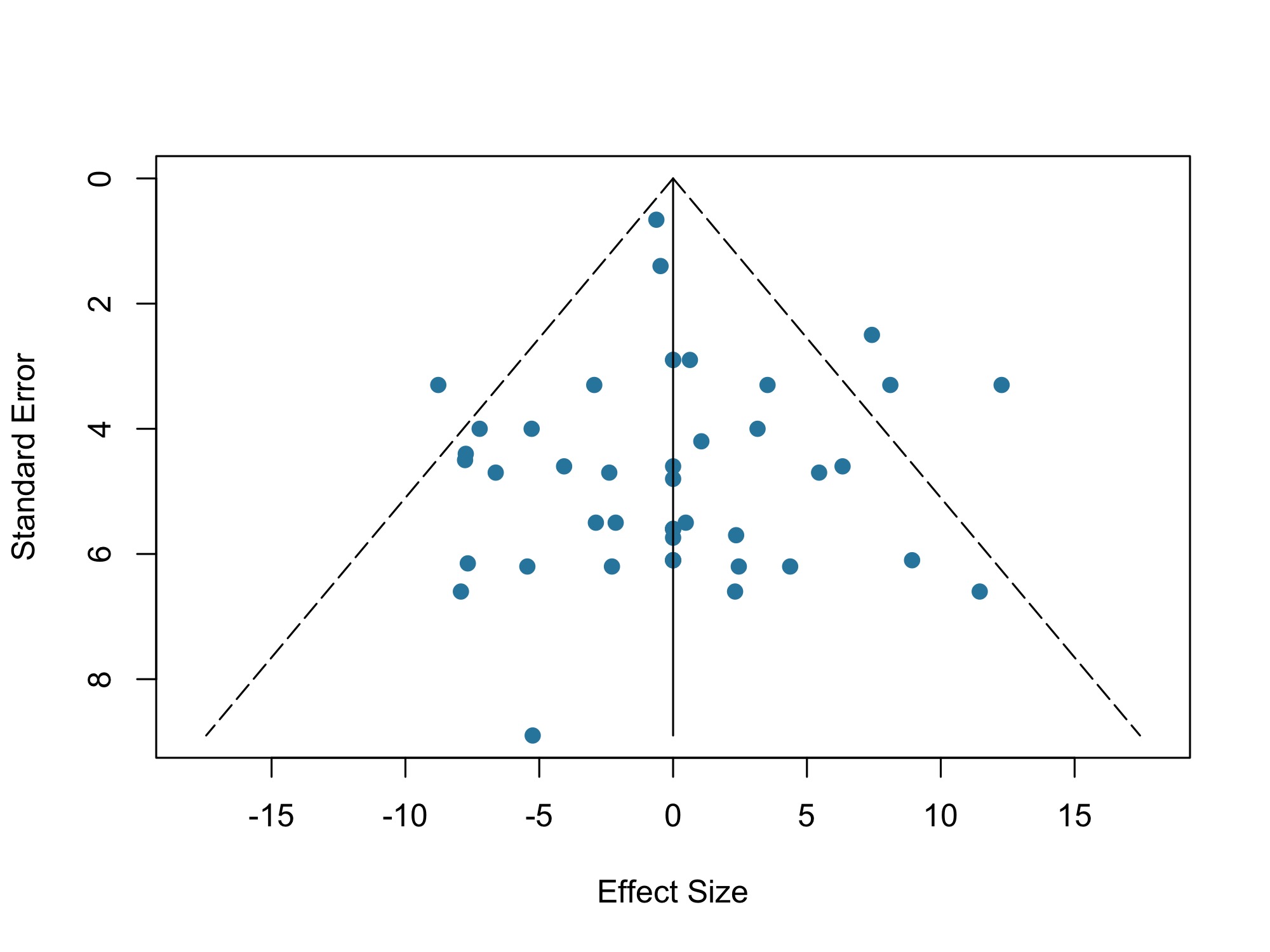


## **5.13. Total Cholesterol**

**
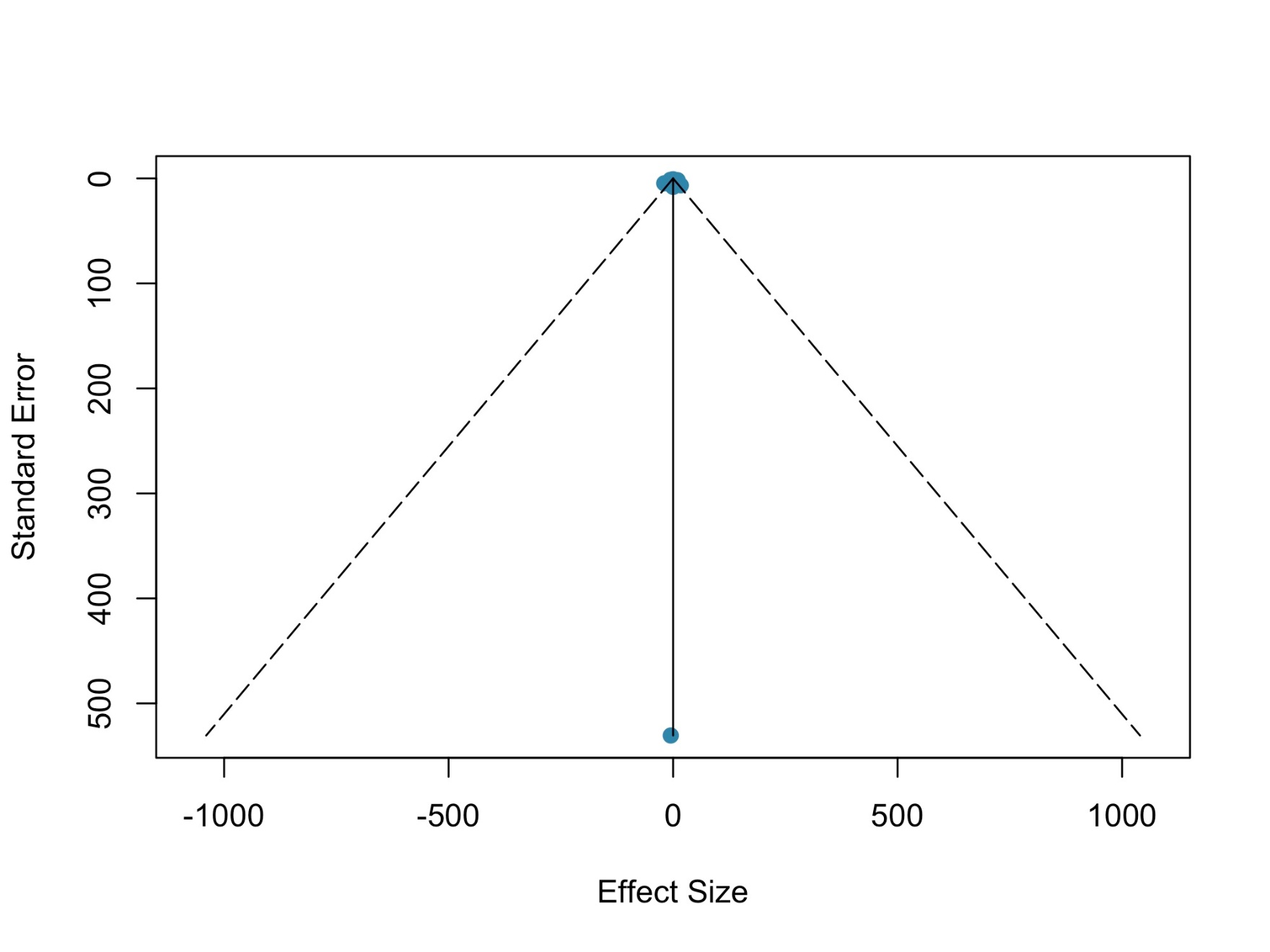
**

## **5.14. Total Cholesterol fixed**


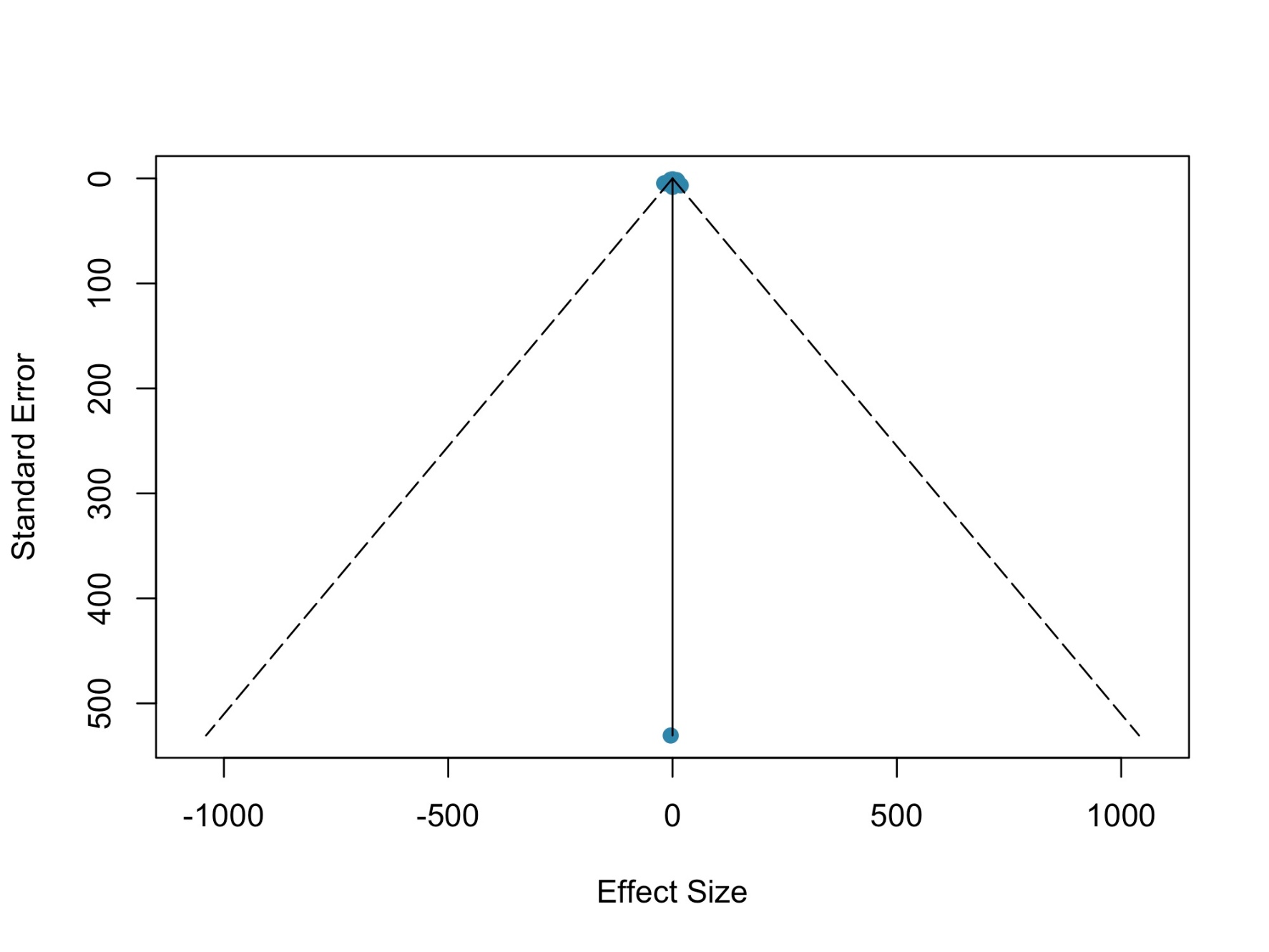


## **5.15. Total Cholesterol Low risk**


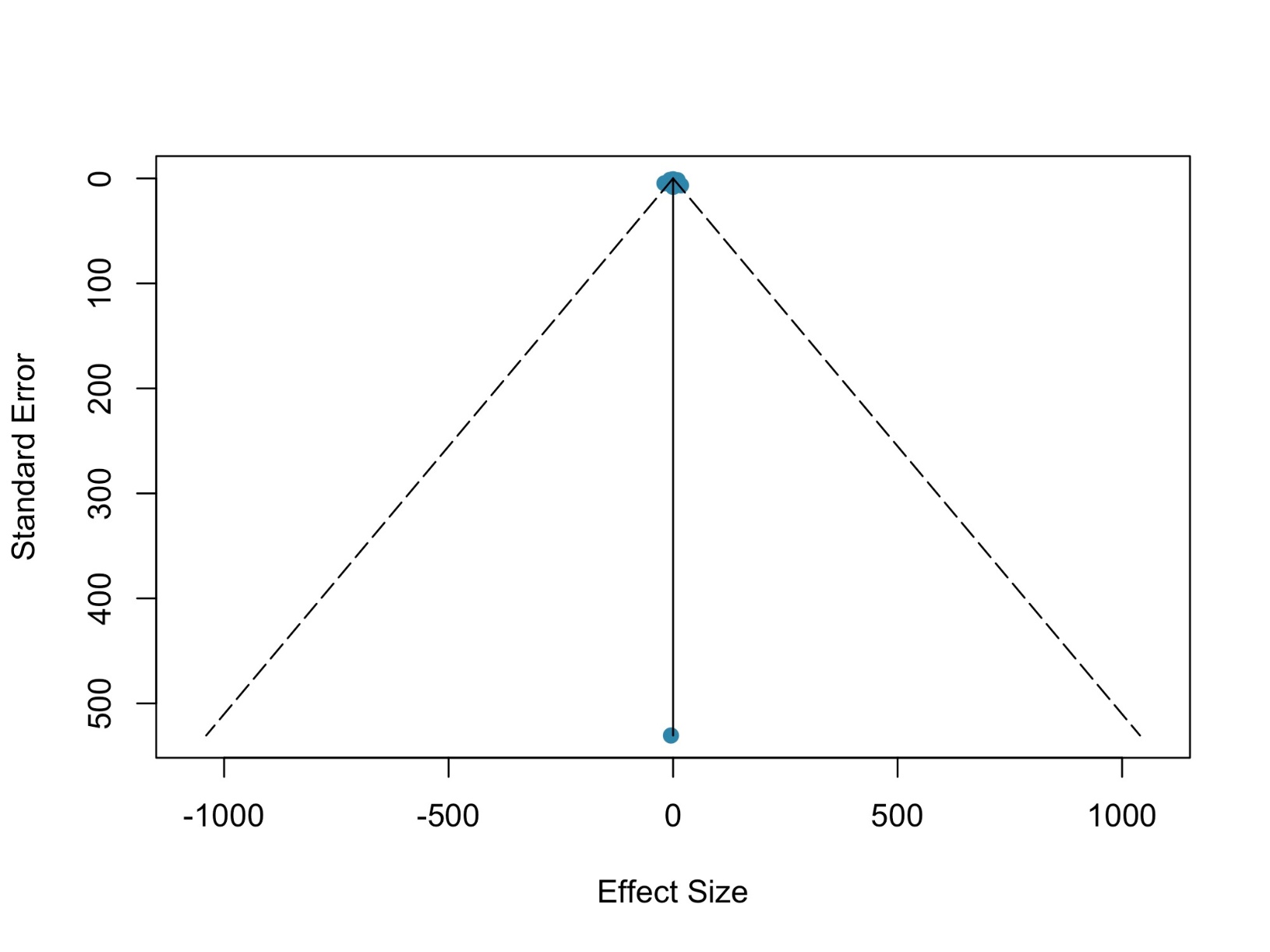


## **5.16. Total Cholesterol Large sample**

**
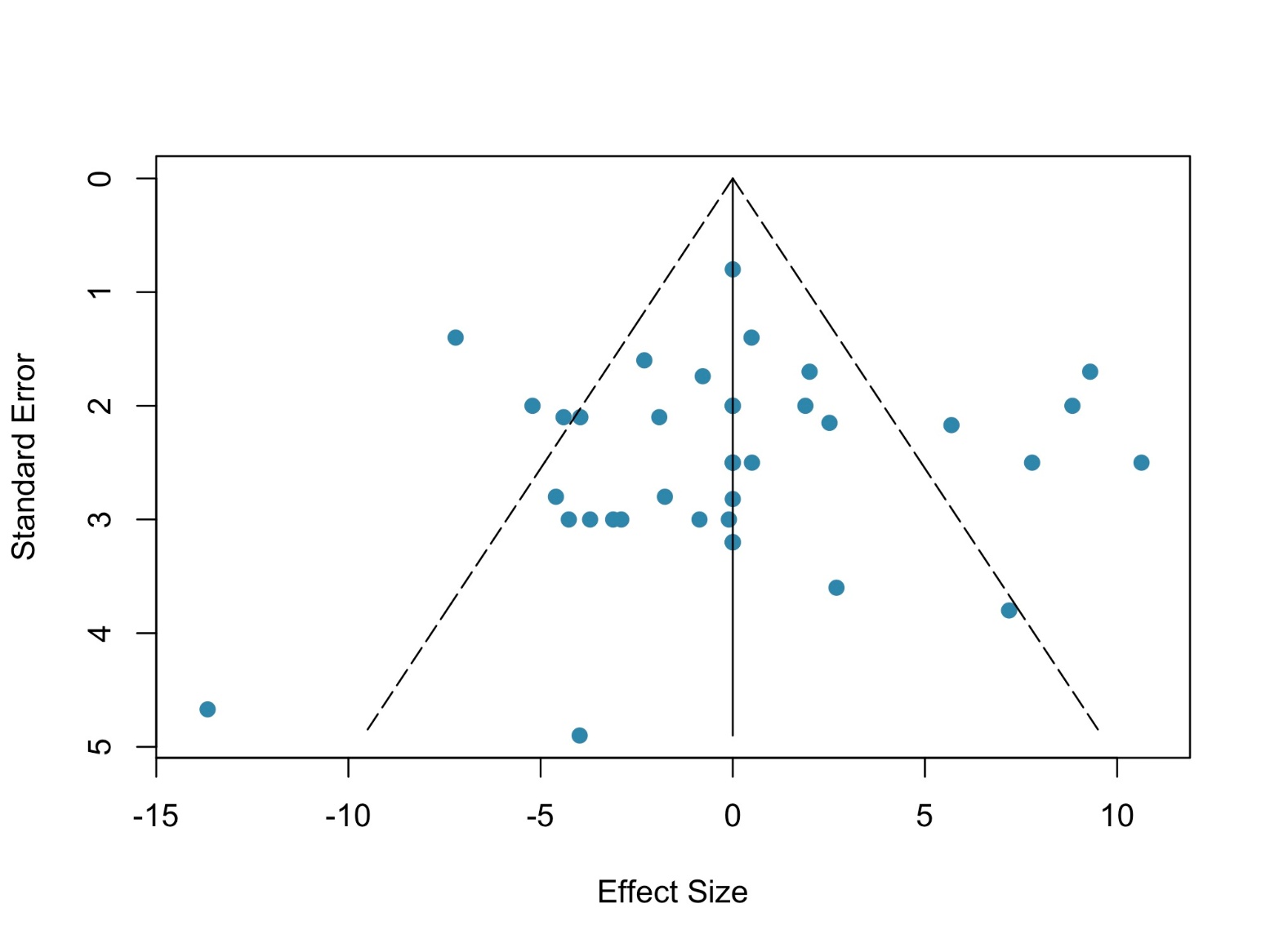
**

## **5.17. HDL**

**
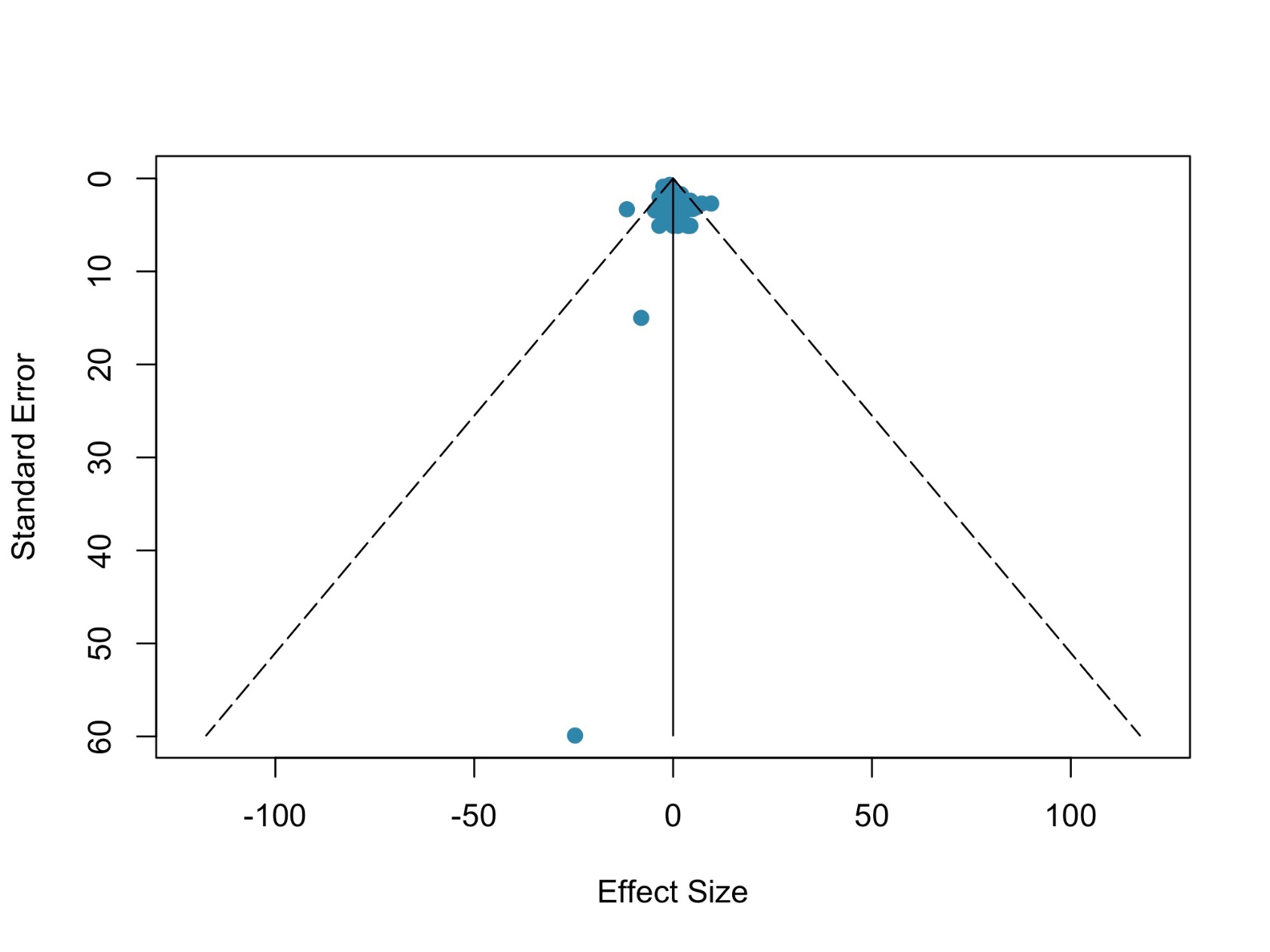
**

## **5.18. HDL fixed**

**
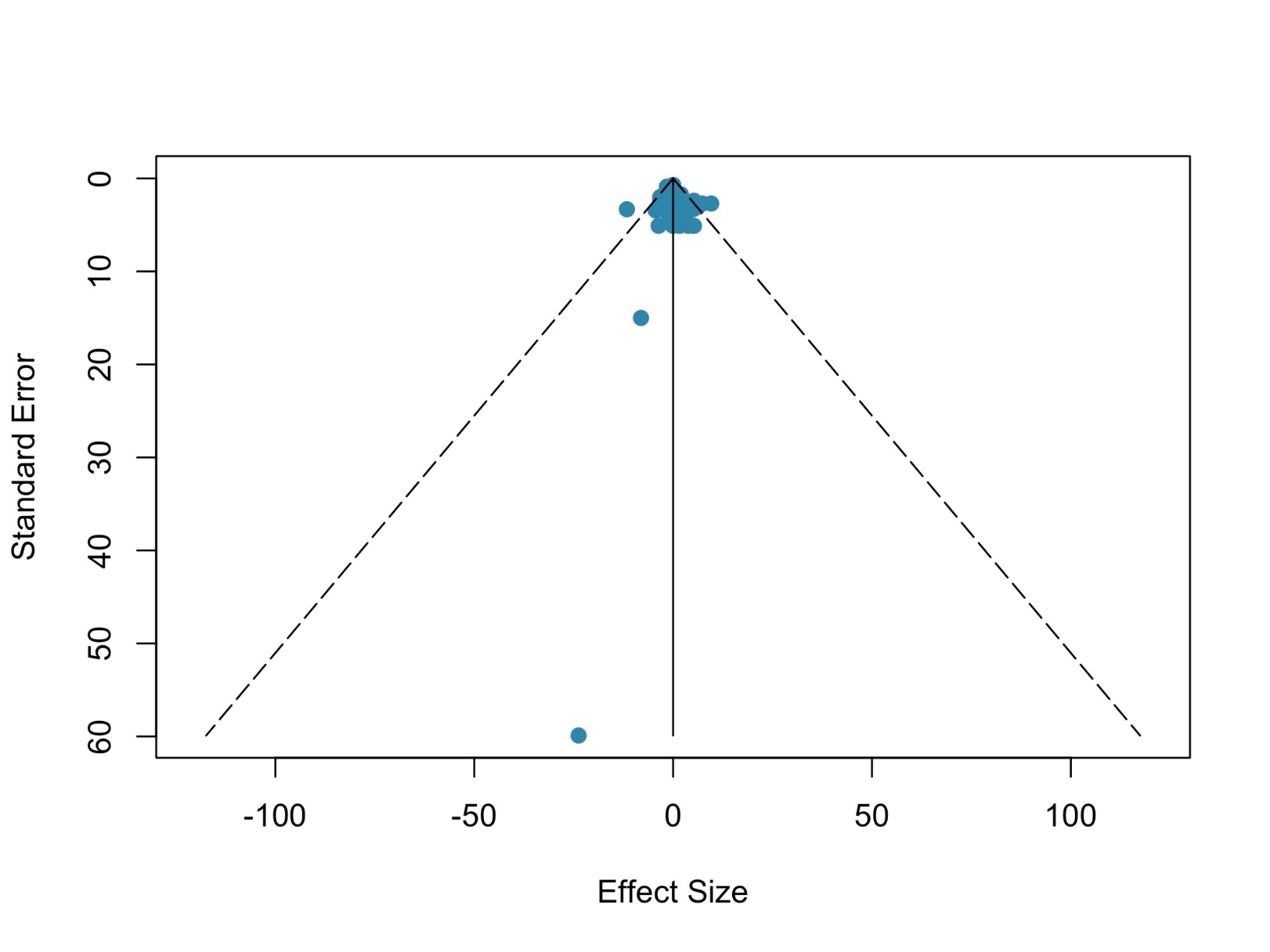
**

## **5.19. HDL Low risk**


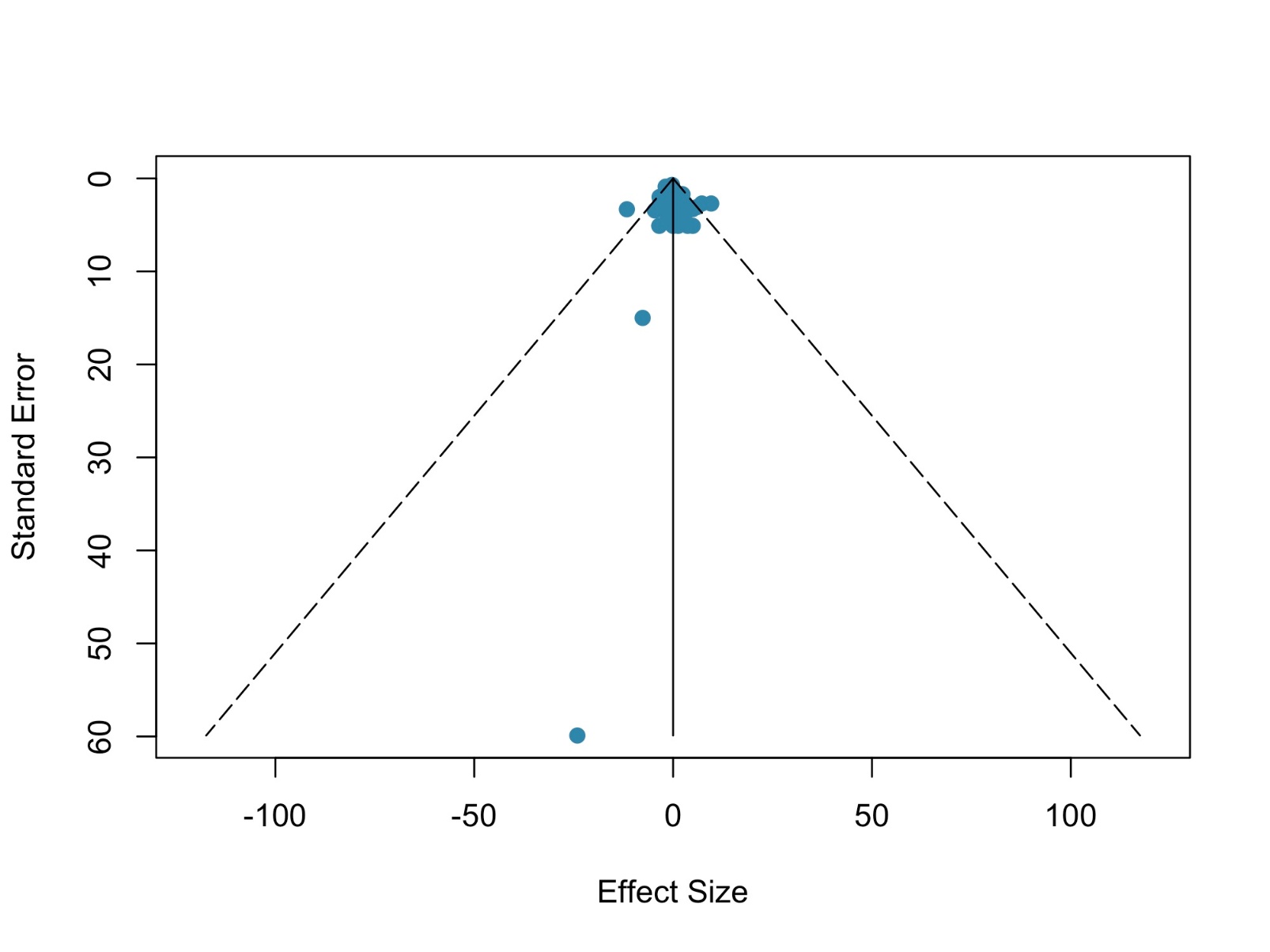


## **5.20. HDL Large sample**


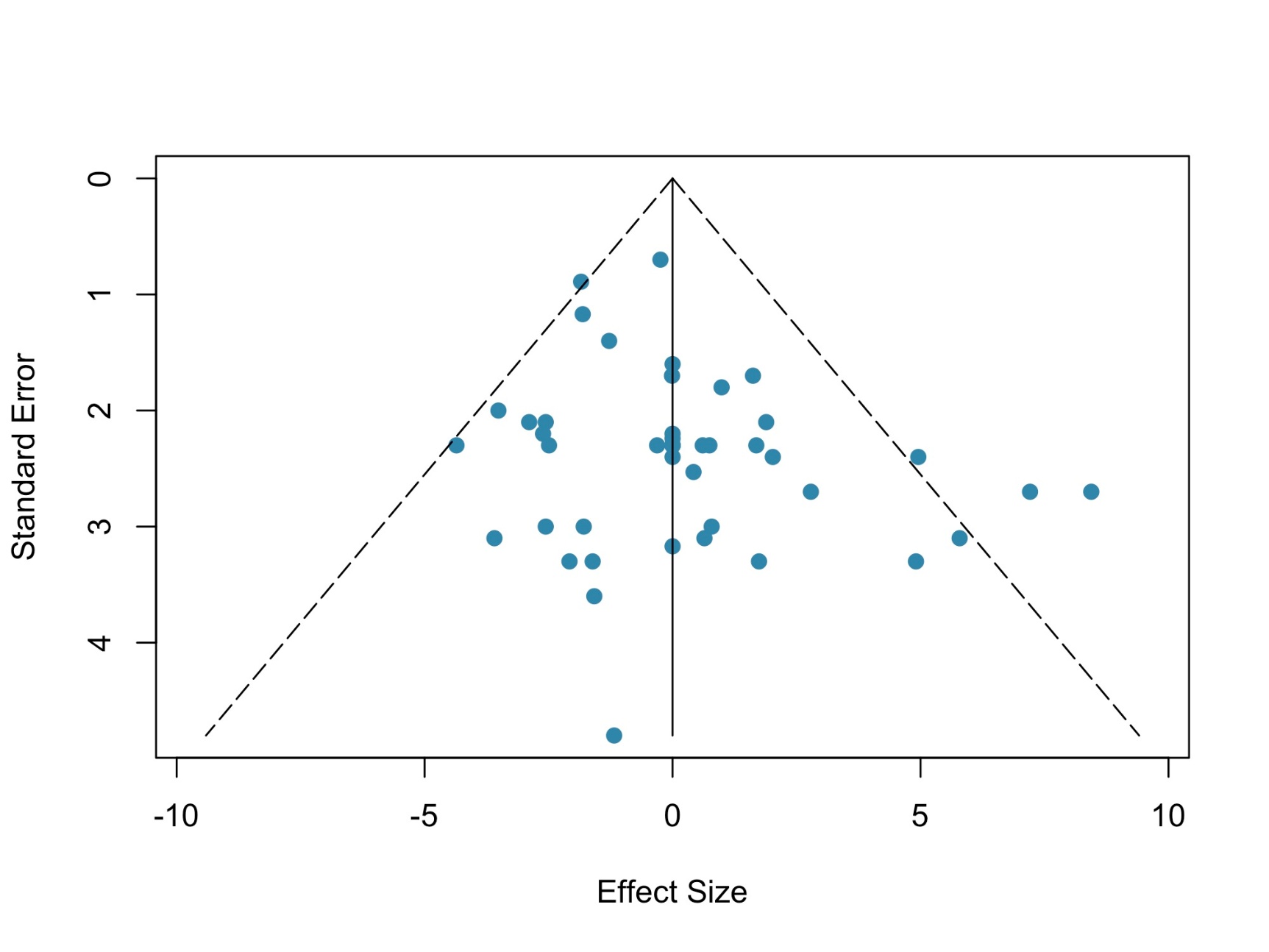


## **5.21. Adverse events**

## **5.21.1. Injection site reactions**

**
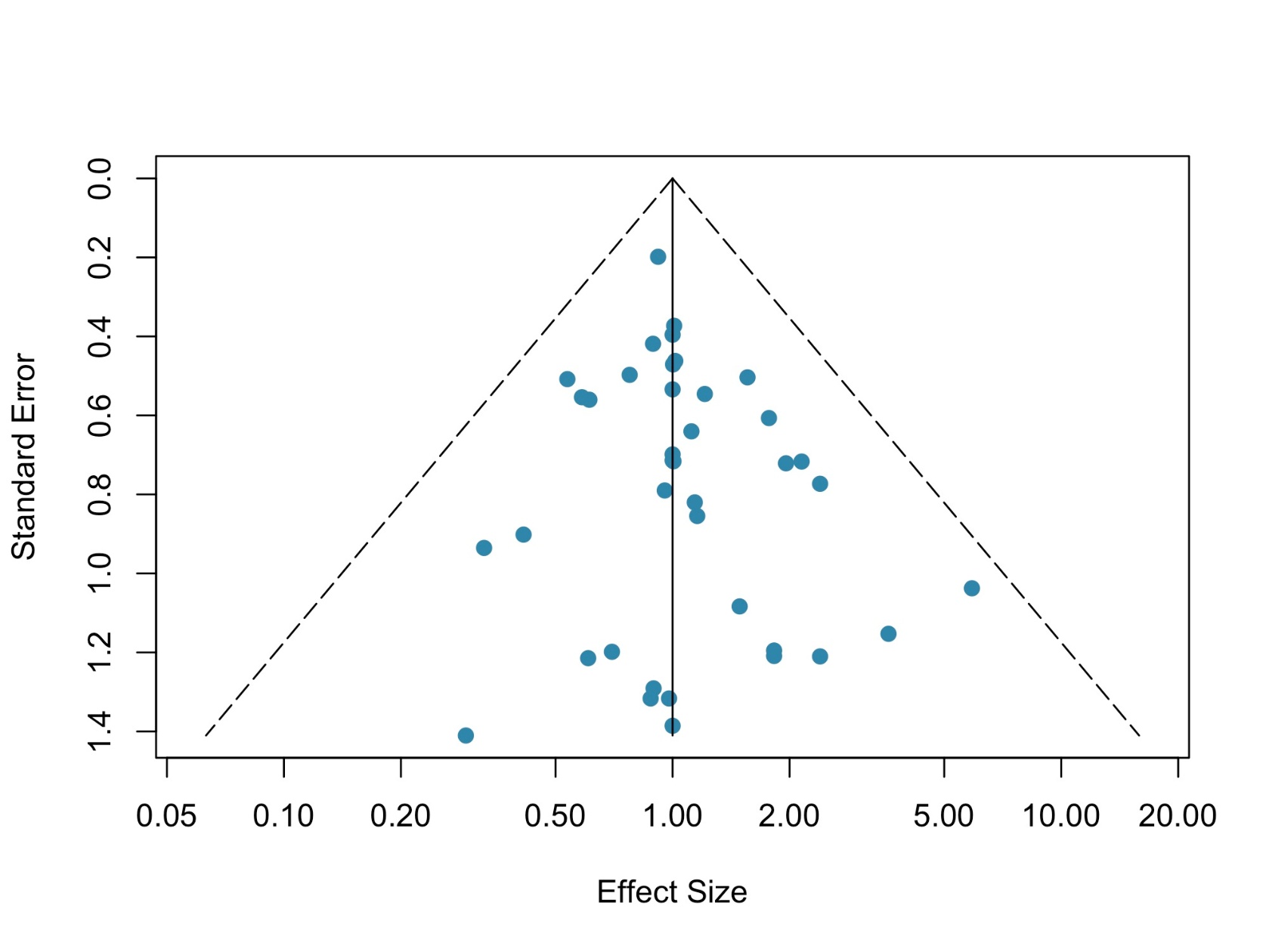
**

## **5.21.2. Injection site reactions- large sample**

**
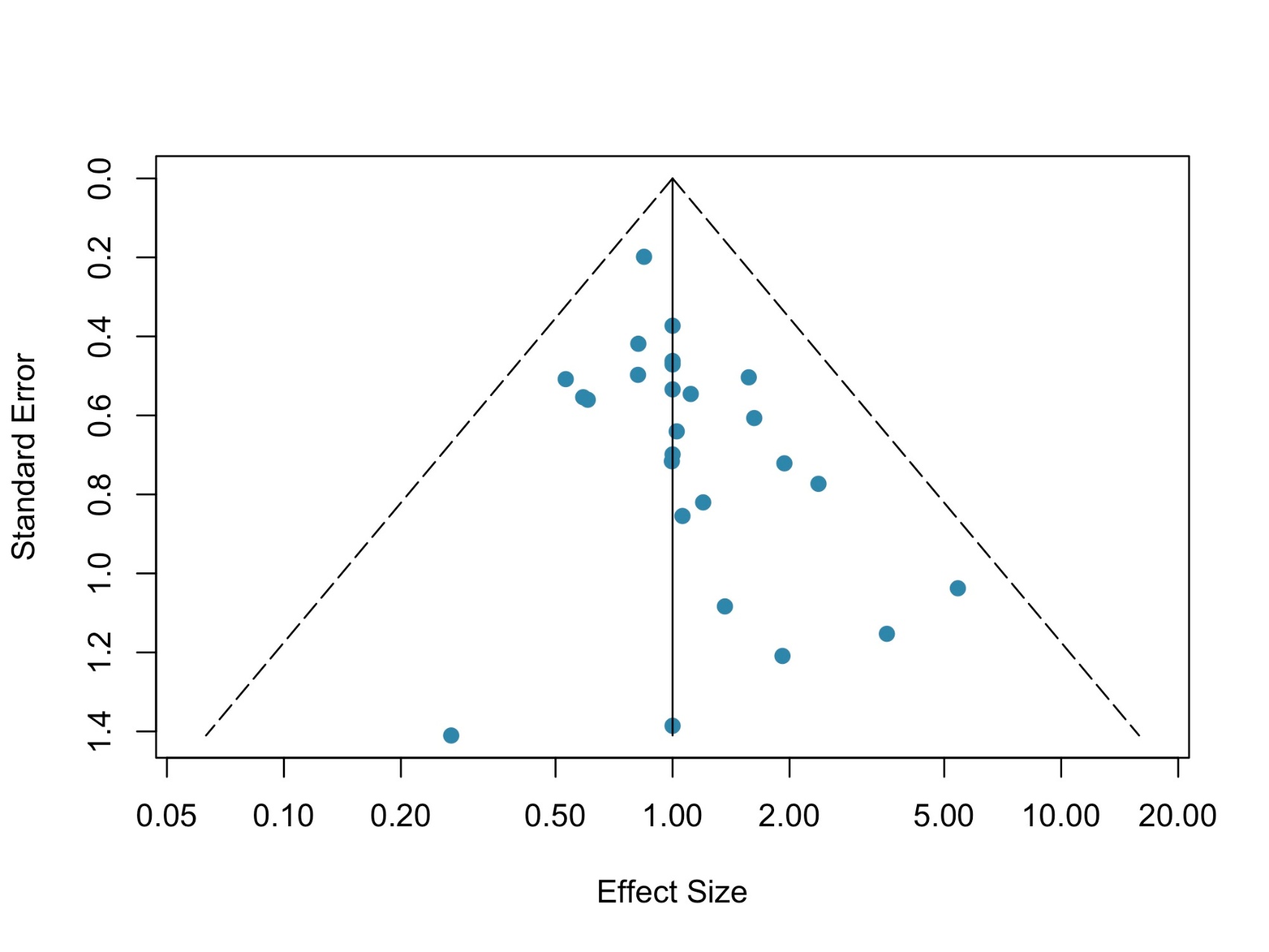
**

## **5.21.3. Injection site reactions – low-risk**


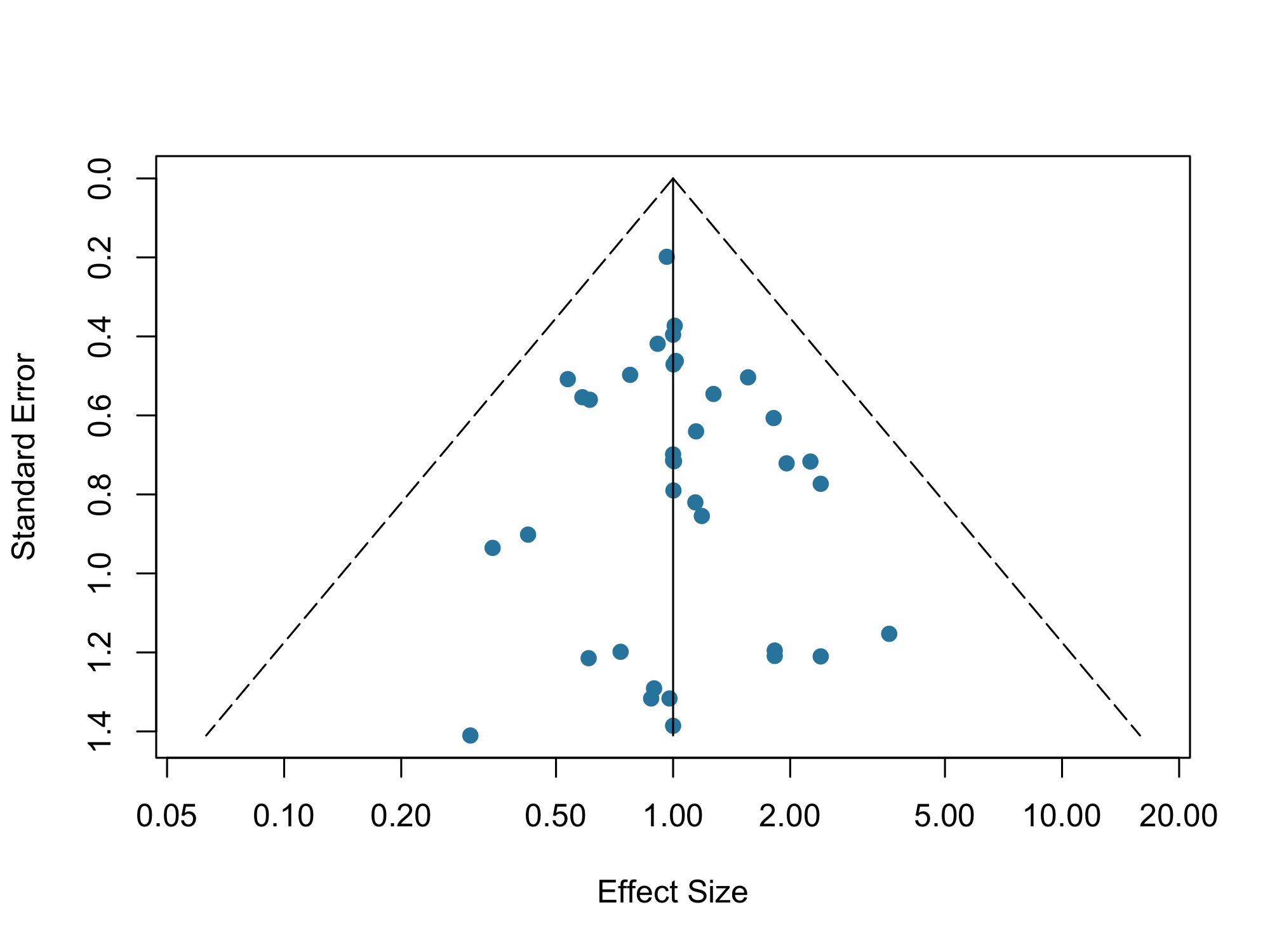


## **5.21.4. Injection site reactions- fixed**

**
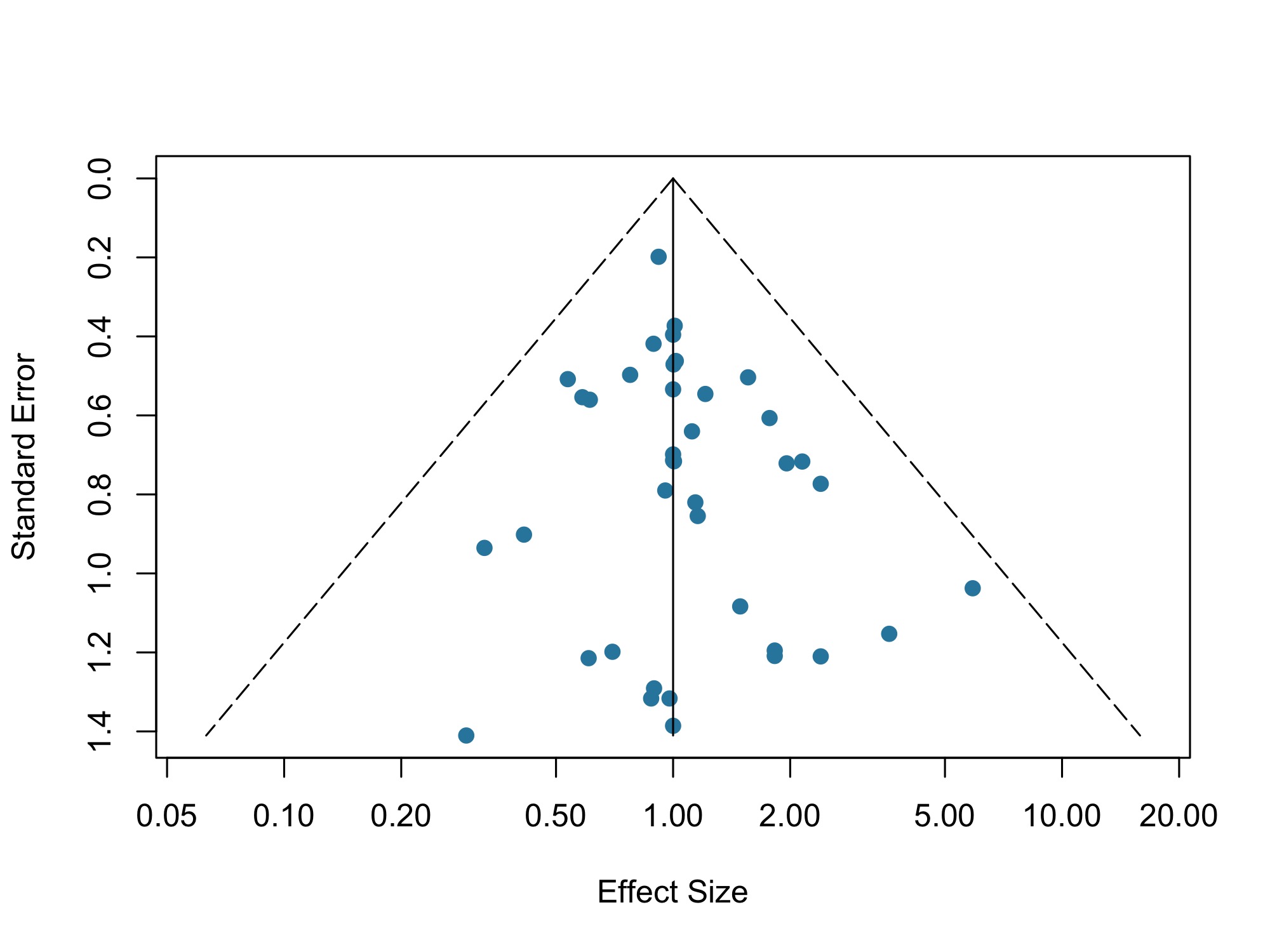
**

## **5.21.5. Serious adverse events**

**
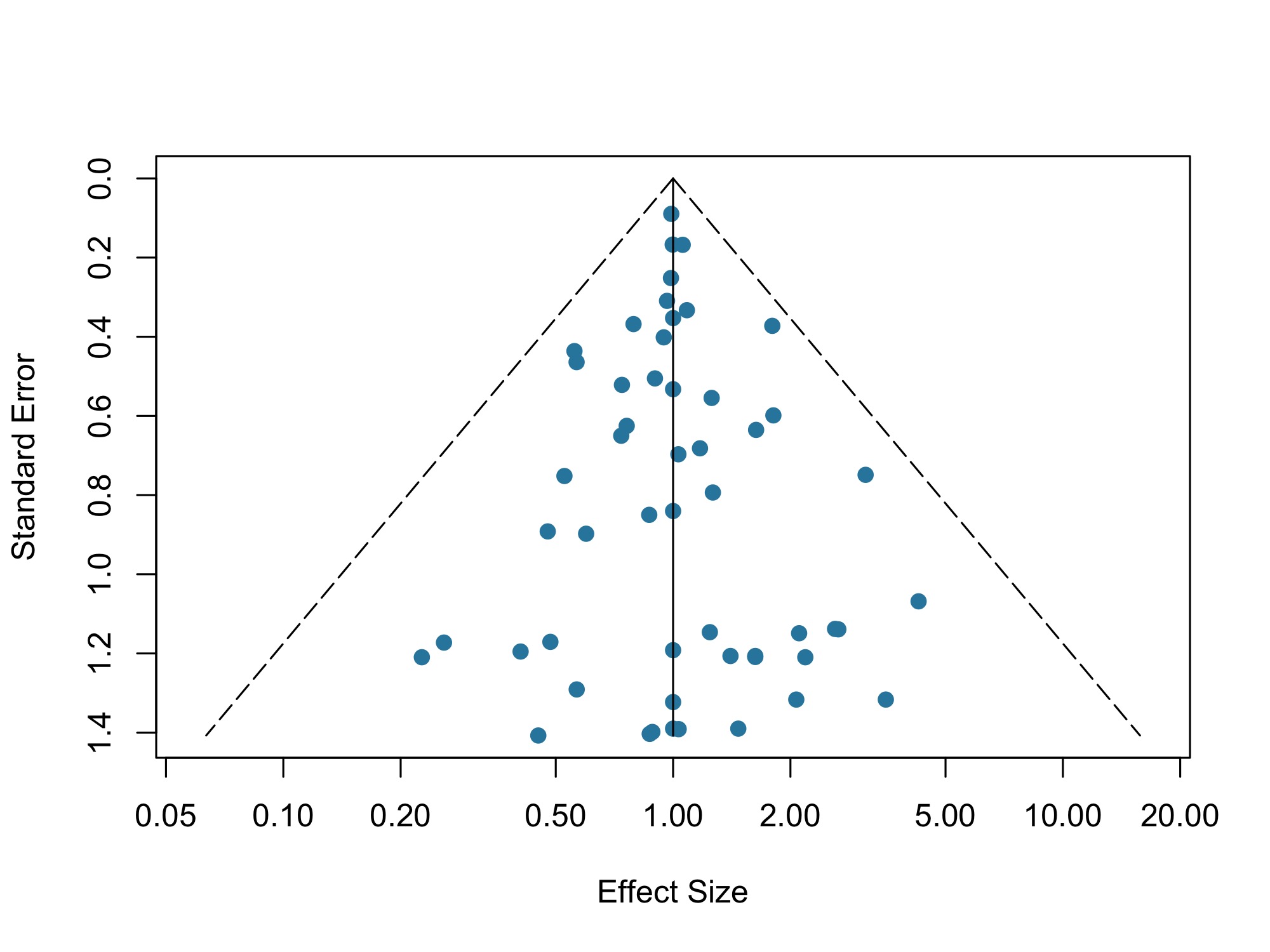
**

## **5.21.6. Serious adverse events- fixed**

**
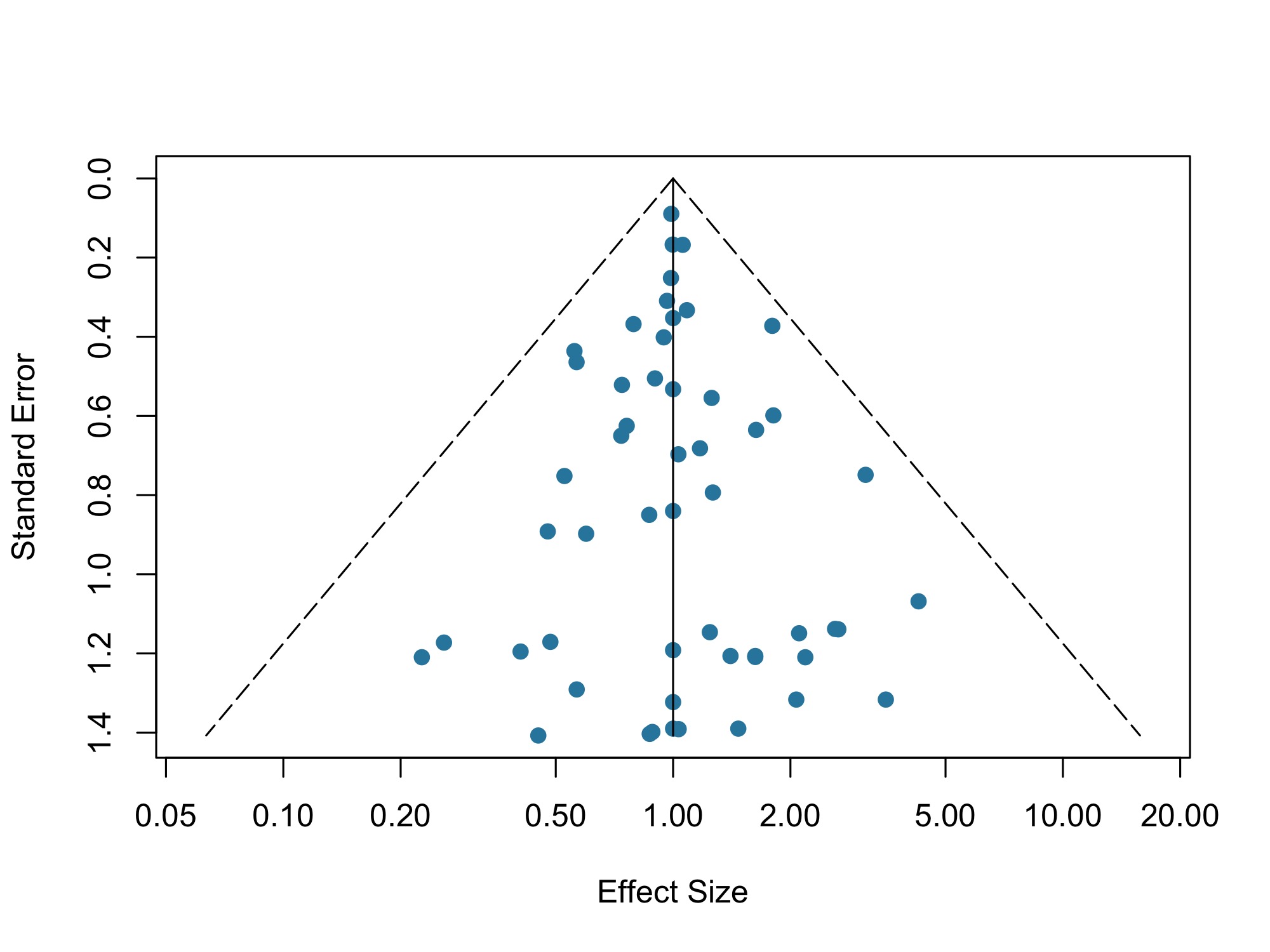
**

### **5.21.7. Serious adverse events- large sample
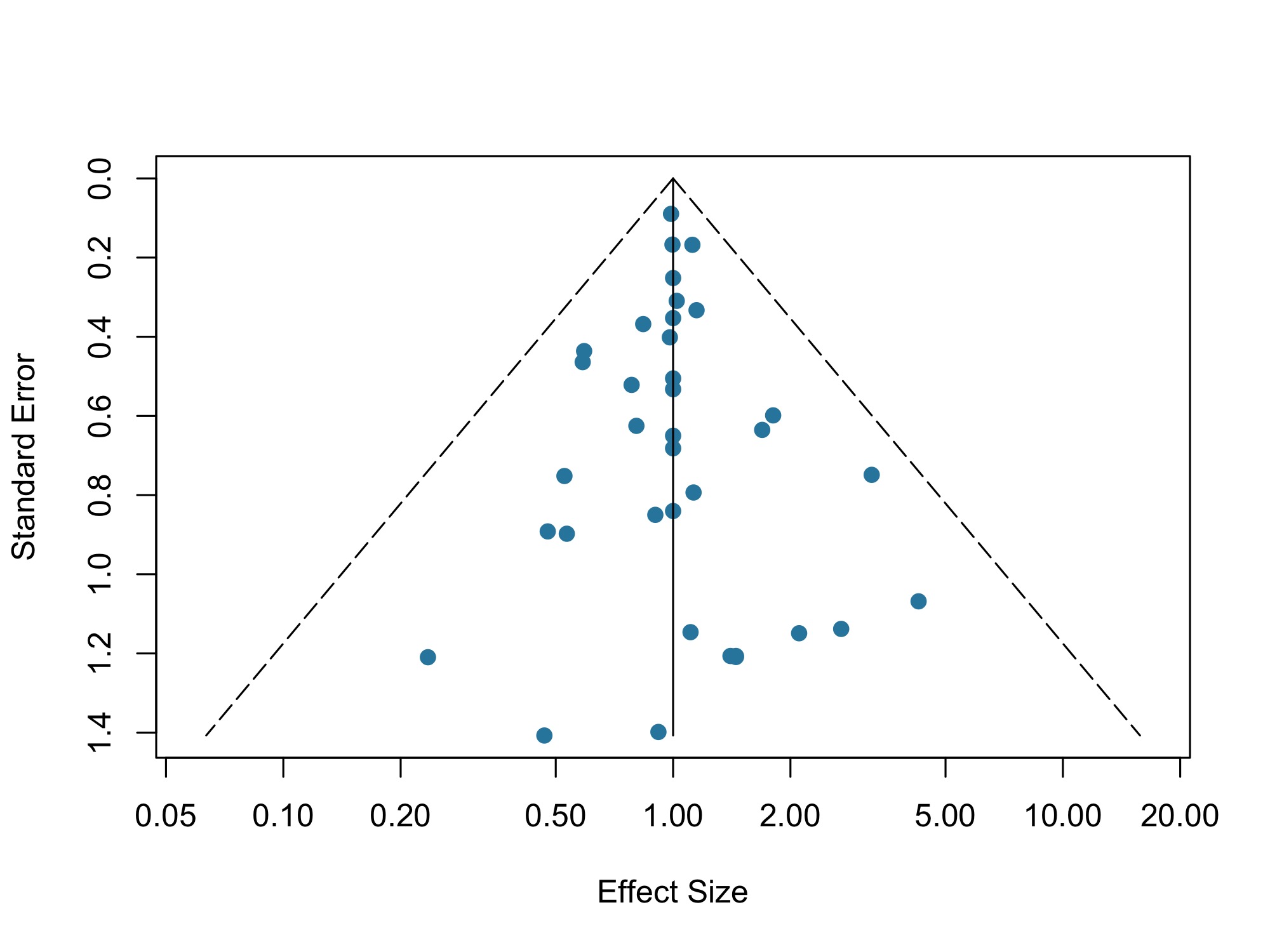
**

### **5.21.8. Serious adverse events- low risk
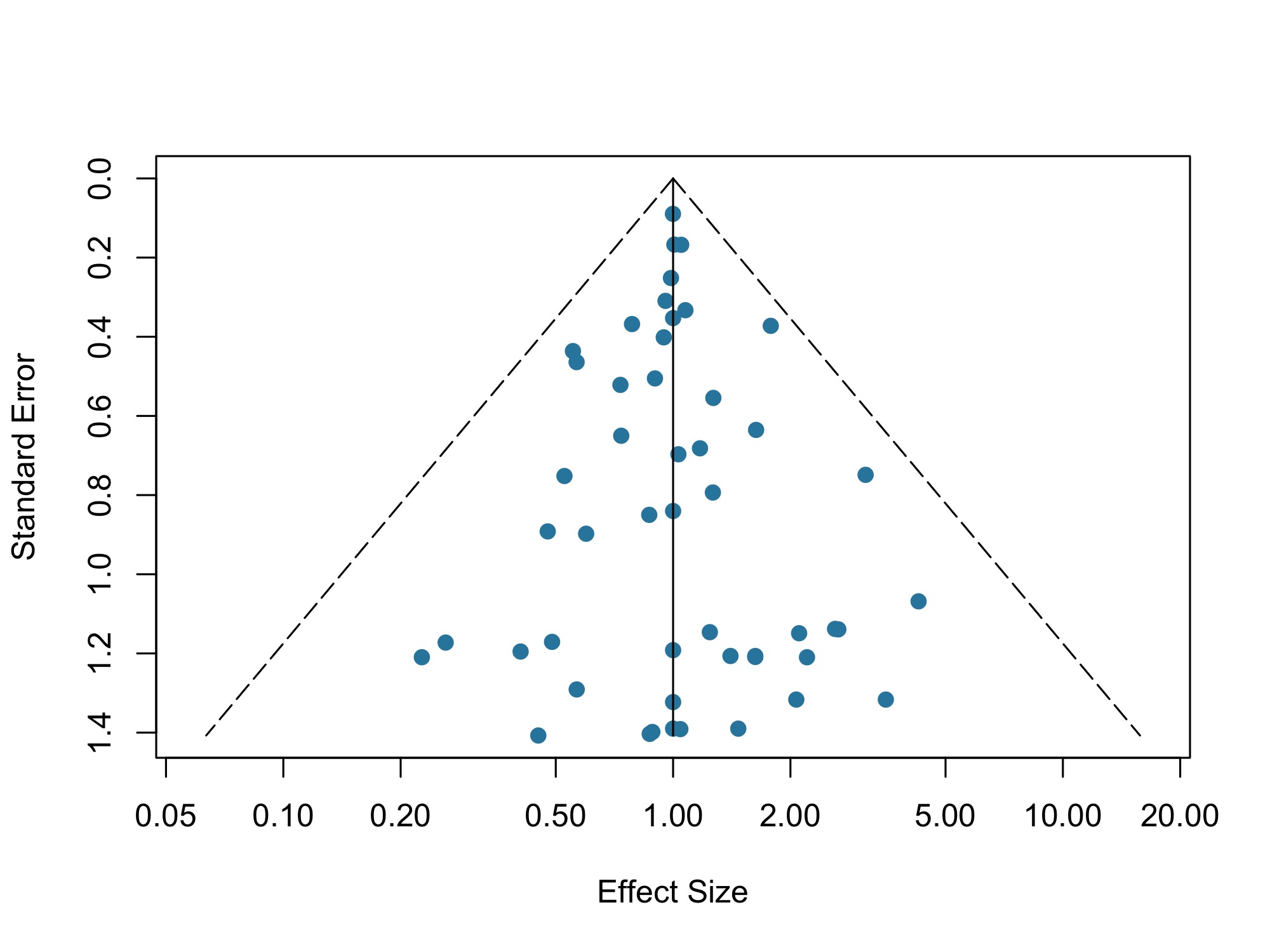
**

### **5.21.9. Treatment discontinuation due to adverse events
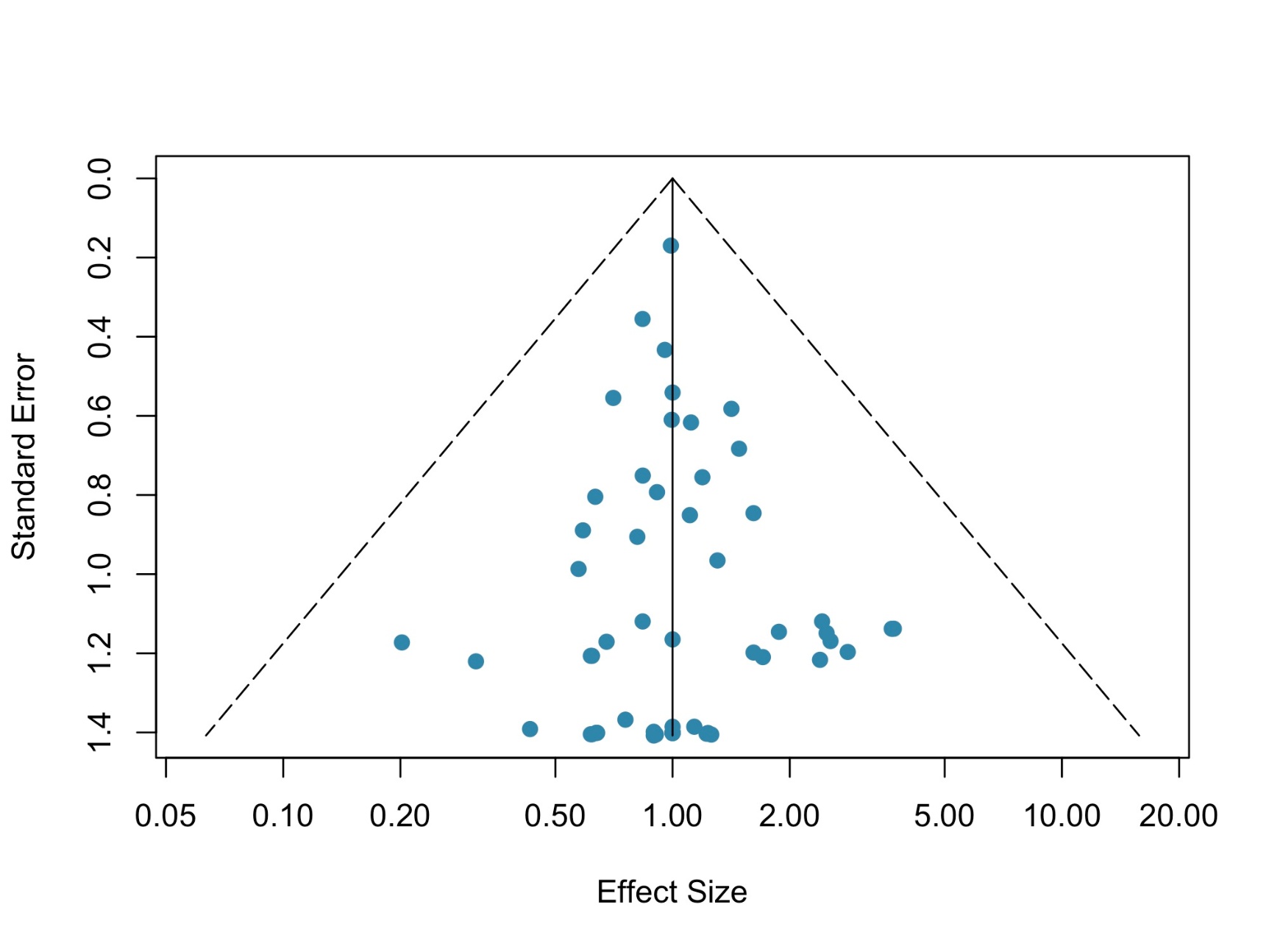
**

### **5.21.10. Treatment discontinuation due to adverse events- fixed**

**
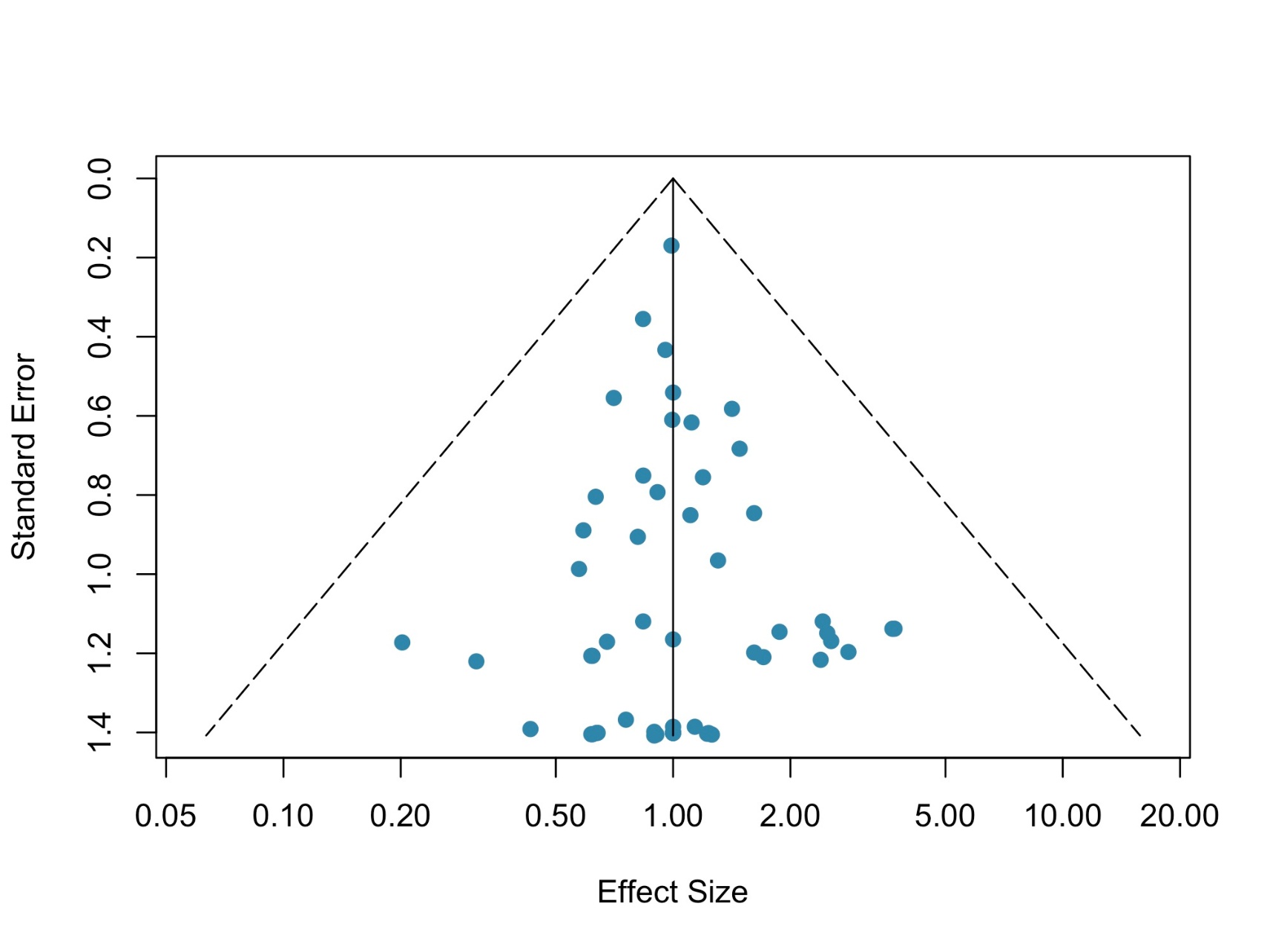
**

### **5.21.11. Treatment discontinuation due to adverse events- large sample**

**
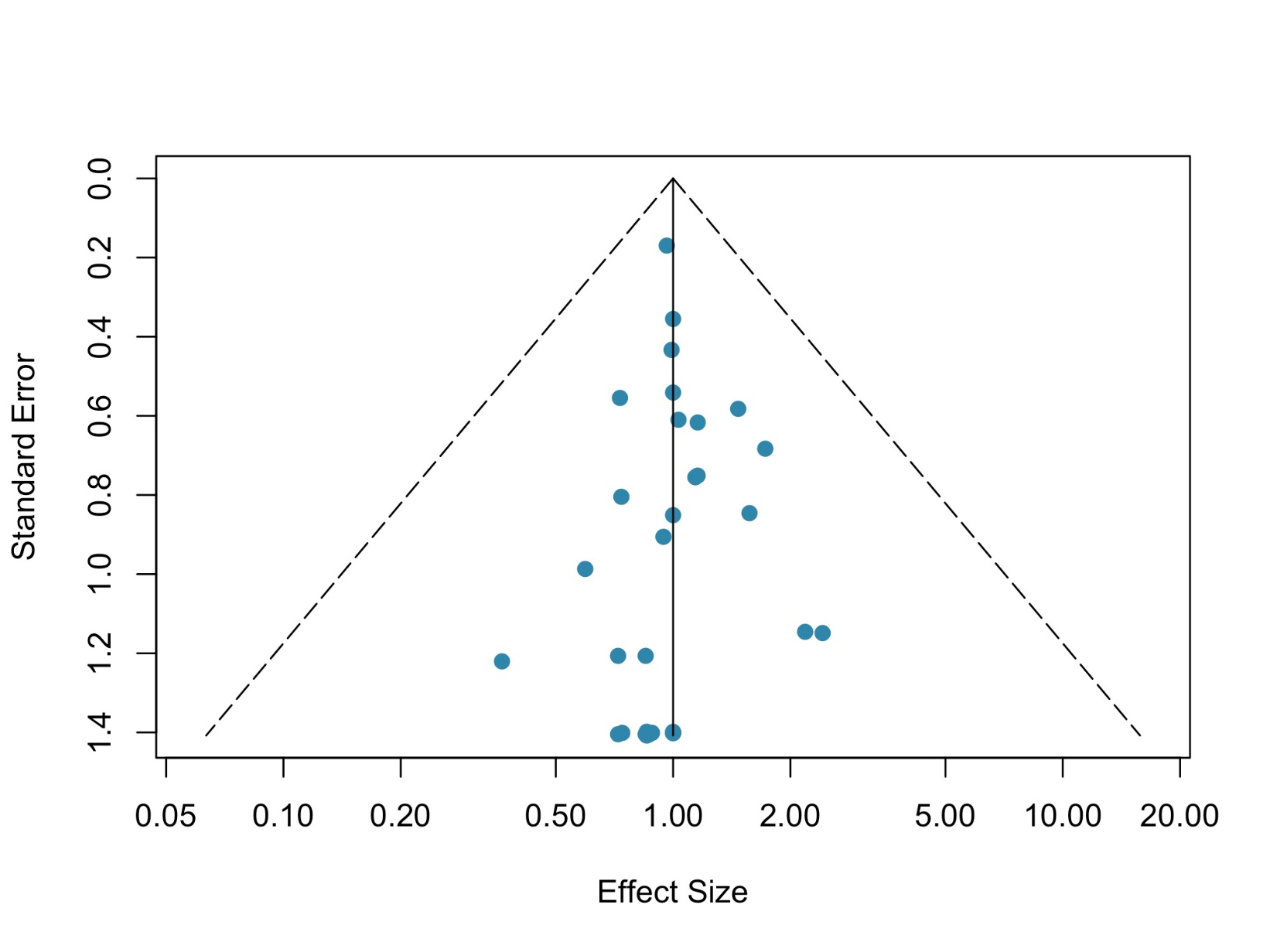
**

### **5.21.12. Treatment discontinuation due to adverse events - low risk**

**
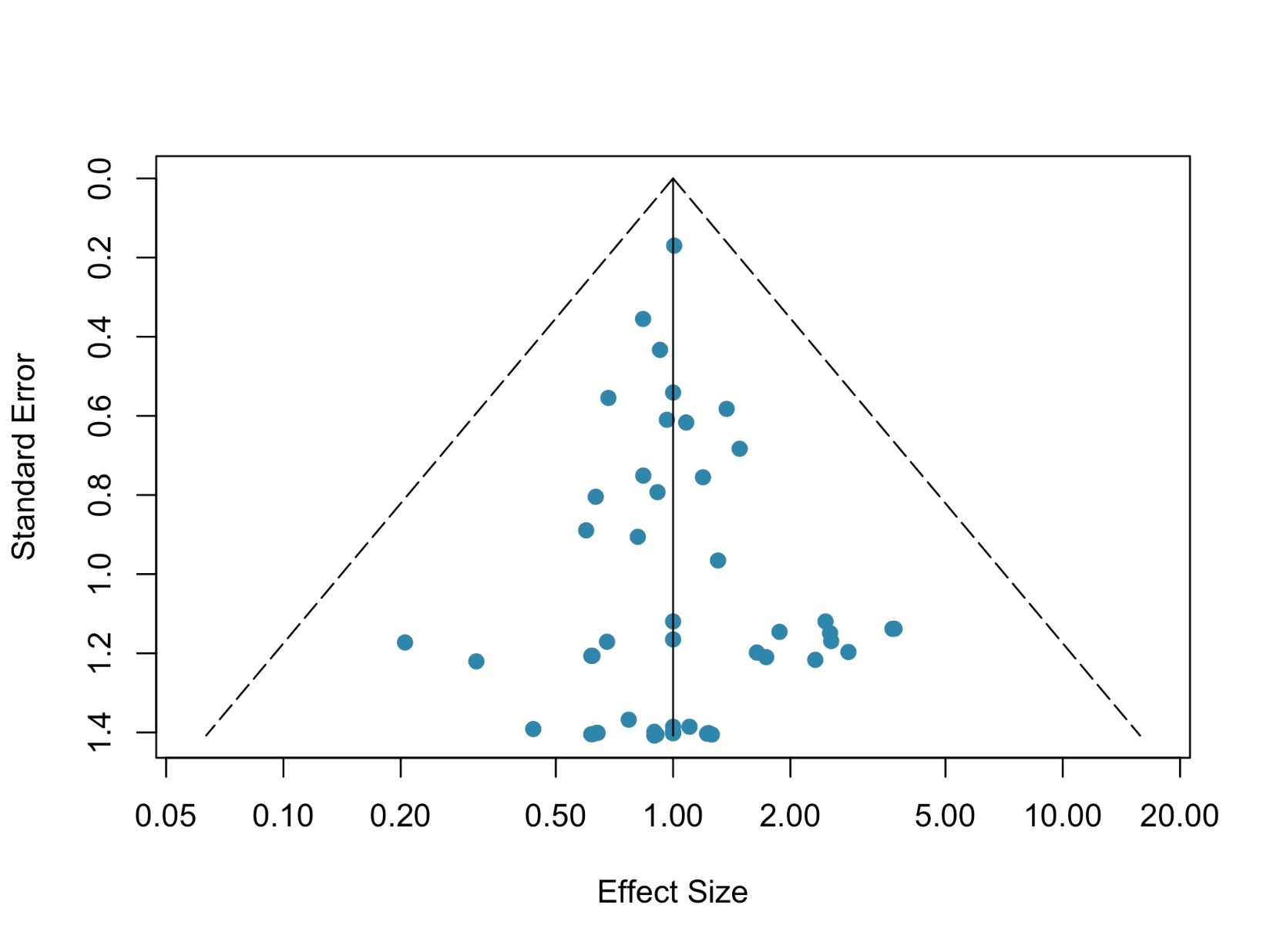
**

**Supplement 6. Network meta-analysis results (league tables)**

**6.1. Network meta-analysis results for change in Lp(a)**

|  | **Alirocumab 150mg Q2W** | **Alirocumab 300mg Q4W** | **Alirocumab 75mg Q2W** | **Evolocumab 140mg Q2W** | **Evolocumab 420mg Q4W** | **Inclisiran 300mg Q3M** | **Lepodisiran 400mg Q6M** | **Lepodisiran 96mg Q6M** | **Muvalaplin 240mg DAILY** | **Muvalaplin 60mg DAILY** | **Olpasiran 225mg Q12W** | **Olpasiran 75mg Q12W** | **Pelacarsen 200mg QW** | **Pelacarsen 80mg QW** | **Placebo** | **Zerlasiran 300mg Q8W** |
| --- | --- | --- | --- | --- | --- | --- | --- | --- | --- | --- | --- | --- | --- | --- | --- | --- |
| **Alirocumab 150mg Q2W** | Alirocumab 150mg Q2W | -19.99 [ -37.53; -2.45] | 1.20 [ -25.10; 27.50] | . | . | . | . | . | . | . | . | . | . | . | -24.56 [ -32.08; -17.05] | . |
| **Alirocumab 300mg Q4W** | -11.32 [ -24.33; 1.69] | Alirocumab 300mg Q4W | -3.40 [ -25.64; 18.84] | . | . | . | . | . | . | . | . | . | . | . | -13.52 [ -27.29; 0.26] | . |
| **Alirocumab 75mg Q2W** | 0.32 [ -10.34; 10.97] | 11.63 [ -2.16; 25.43] | Alirocumab 75mg Q2W | . | . | . | . | . | . | . | . | . | . | . | -27.20 [ -35.80; -18.61] | . |
| **Evolocumab 140mg Q2W** | 6.33 [ -4.01; 16.67] | 17.65 [ 3.39; 31.91] | 6.02 [ -5.06; 17.09] | Evolocumab 140mg Q2W | -4.77 [ -13.24; 3.69] | . | . | . | . | . | . | . | . | . | -32.59 [ -40.32; -24.87] | . |
| **Evolocumab 420mg Q4W** | 1.32 [ -7.98; 10.62] | 12.64 [ -0.89; 26.17] | 1.00 [ -9.11; 11.12] | -5.01 [ -12.57; 2.54] | Evolocumab 420mg Q4W | . | . | . | . | . | . | . | . | . | -27.02 [ -32.77; -21.27] | . |
| **Inclisiran 300mg Q3M** | 1.91 [ -13.58; 17.41] | 13.23 [ -5.11; 31.58] | 1.60 [ -14.39; 17.59] | -4.42 [ -19.87; 11.03] | 0.59 [ -14.18; 15.37] | Inclisiran 300mg Q3M | . | . | . | . | . | . | . | . | -27.17 [ -40.80; -13.53] | . |
| **Lepodisiran 400mg Q6M** | 56.33 [ 34.25; 78.41] | 67.65 [ 43.48; 91.82] | 56.01 [ 33.58; 78.45] | 50.00 [ 27.94; 72.05] | 55.01 [ 33.42; 76.60] | 54.42 [ 29.53; 79.31] | Lepodisiran 400mg Q6M | -28.40 [ -50.41; -6.39] | . | . | . | . | . | . | -74.20 [ -96.21; -52.19] | . |
| **Lepodisiran 96mg Q6M** | 35.31 [ 17.00; 53.62] | 46.63 [ 25.85; 67.41] | 35.00 [ 16.26; 53.73] | 28.98 [ 10.70; 47.26] | 33.99 [ 16.28; 51.70] | 33.40 [ 11.79; 55.01] | -21.02 [ -41.84; -0.20] | Lepodisiran 96mg Q6M | . | . | . | . | . | . | -60.56 [ -77.33; -43.80] | . |
| **Muvalaplin 240mg DAILY** | 60.55 [ 37.79; 83.31] | 71.87 [ 47.08; 96.65] | 60.23 [ 37.13; 83.33] | 54.22 [ 31.49; 76.94] | 59.23 [ 36.95; 81.50] | 58.63 [ 33.15; 84.12] | 4.22 [ -25.74; 34.17] | 25.24 [ -2.06; 52.53] | Muvalaplin 240mg DAILY | -4.10 [ -25.64; 17.44] | . | . | . | . | -85.80 [-107.34; -64.26] | . |
| **Muvalaplin 60mg DAILY** | 56.45 [ 33.69; 79.21] | 67.77 [ 42.98; 92.55] | 56.13 [ 33.03; 79.23] | 50.12 [ 27.39; 72.84] | 55.13 [ 32.85; 77.40] | 54.53 [ 29.05; 80.02] | 0.12 [ -29.84; 30.07] | 21.14 [ -6.16; 48.43] | -4.10 [ -25.64; 17.44] | Muvalaplin 60mg DAILY | . | . | . | . | -81.70 [-103.24; -60.16] | . |
| **Olpasiran 225mg Q12W** | 73.69 [ 56.60; 90.77] | 85.01 [ 65.30; 104.71] | 73.37 [ 55.83; 90.91] | 67.36 [ 50.31; 84.40] | 72.37 [ 55.93; 88.81] | 71.77 [ 51.19; 92.36] | 17.36 [ -8.55; 43.27] | 38.37 [ 15.59; 61.16] | 13.14 [ -13.35; 39.63] | 17.24 [ -9.25; 43.73] | Olpasiran 225mg Q12W | -3.15 [ -18.57; 12.27] | . | . | -98.94 [-114.36; -83.52] | . |
| **Olpasiran 75mg Q12W** | 70.54 [ 53.45; 87.63] | 81.86 [ 62.15; 101.57] | 70.22 [ 52.69; 87.76] | 64.21 [ 47.16; 81.26] | 69.22 [ 52.78; 85.66] | 68.63 [ 48.04; 89.21] | 14.21 [ -11.70; 40.12] | 35.23 [ 12.45; 58.01] | 9.99 [ -16.50; 36.48] | 14.09 [ -12.40; 40.58] | -3.15 [ -18.57; 12.27] | Olpasiran 75mg Q12W | . | . | -95.79 [-111.21; -80.37] | . |
| **Pelacarsen 200mg QW** | 26.15 [ 6.91; 45.38] | 37.47 [ 15.87; 59.06] | 25.83 [ 6.19; 45.47] | 19.81 [ 0.61; 39.02] | 24.83 [ 6.16; 43.49] | 24.23 [ 1.83; 46.64] | -30.18 [ -57.56; -2.81] | -9.17 [ -33.60; 15.27] | -34.40 [ -62.33; -6.48] | -30.30 [ -58.23; -2.38] | -47.54 [ -71.07; -24.01] | -44.39 [ -67.93; -20.86] | Pelacarsen 200mg QW | . | -51.40 [ -69.17; -33.62] | . |
| **Pelacarsen 80mg QW** | 66.11 [ 47.83; 84.39] | 77.43 [ 56.68; 98.18] | 65.79 [ 47.09; 84.49] | 59.78 [ 41.54; 78.02] | 64.79 [ 47.12; 82.46] | 64.20 [ 42.61; 85.78] | 9.78 [ -16.93; 36.49] | 30.80 [ 7.11; 54.48] | 5.56 [ -21.71; 32.83] | 9.66 [ -17.61; 36.93] | -7.58 [ -30.33; 15.17] | -4.43 [ -27.18; 18.32] | 39.96 [ 15.55; 64.37] | Pelacarsen 80mg QW | -91.36 [-108.09; -74.63] | . |
| **Placebo** | -25.25 [ -32.61; -17.89] | -13.93 [ -26.20; -1.66] | -25.57 [ -33.92; -17.21] | -31.58 [ -38.85; -24.32] | -26.57 [ -32.27; -20.88] | -27.17 [ -40.80; -13.53] | -81.58 [-102.40; -60.76] | -60.56 [ -77.33; -43.80] | -85.80 [-107.34; -64.26] | -81.70 [-103.24; -60.16] | -98.94 [-114.36; -83.52] | -95.79 [-111.21; -80.37] | -51.40 [ -69.17; -33.62] | -91.36 [-108.09; -74.63] | Placebo | 82.71 [ 60.28; 105.15] |
| **Zerlasiran 300mg Q8W** | 57.46 [ 33.85; 81.07] | 68.78 [ 43.21; 94.35] | 57.15 [ 33.21; 81.08] | 51.13 [ 27.55; 74.71] | 56.14 [ 33.00; 79.29] | 55.55 [ 29.30; 81.80] | 1.13 [ -29.47; 31.74] | 22.15 [ -5.86; 50.16] | -3.09 [ -34.18; 28.01] | 1.01 [ -30.08; 32.11] | -16.22 [ -43.45; 11.00] | -13.08 [ -40.30; 14.14] | 31.32 [ 2.70; 59.94] | -8.65 [ -36.63; 19.34] | 82.71 [ 60.28; 105.15] | Zerlasiran 300mg Q8W |

The network meta-analysis outcomes are detailed in the lower half, while the upper half displays pairwise meta-analysis results. Primary treatments are listed in order of efficacy ranking.

## **6.2. Network meta-analysis results for change in LDL-C**

|  | **Alirocumab 150mg Q2W** | **Alirocumab 300mg Q4W** | **Alirocumab 75mg Q2W** | **Evolocumab 140mg Q2W** | **Evolocumab 420mg Q4W** | **Inclisiran 300mg Q3M** | **Lepodisiran 400mg Q6M** | **Lepodisiran 96mg Q6M** | **Muvalaplin 240mg DAILY** | **Muvalaplin 60mg DAILY** | **Olpasiran 225mg Q12W** | **Olpasiran 75mg Q12W** | **Pelacarsen 200mg QW** | **Pelacarsen 80mg QW** | **Placebo** | **Zerlasiran 300mg Q8W** |
| --- | --- | --- | --- | --- | --- | --- | --- | --- | --- | --- | --- | --- | --- | --- | --- | --- |
| **Alirocumab 150mg Q2W** | Alirocumab 150mg Q2W | -25.40 [-47.56; -3.24] | -9.50 [-28.98; 9.98] | . | . | . | . | . | . | . | . | . | . | . | -56.74 [-64.38; -49.10] | . |
| **Alirocumab 300mg Q4W** | -16.00 [-28.26; -3.73] | Alirocumab 300mg Q4W | 0.10 [-18.90; 19.10] | . | . | . | . | . | . | . | . | . | . | . | -40.42 [-52.04; -28.80] | . |
| **Alirocumab 75mg Q2W** | -8.18 [-17.41; 1.06] | 7.82 [ -3.85; 19.48] | Alirocumab 75mg Q2W | . | . | . | . | . | . | . | . | . | . | . | -49.74 [-56.16; -43.32] | . |
| **Evolocumab 140mg Q2W** | 3.62 [ -5.42; 12.67] | 19.62 [ 7.73; 31.51] | 11.80 [ 3.64; 19.96] | Evolocumab 140mg Q2W | -3.03 [ -8.95; 2.88] | . | . | . | . | . | . | . | . | . | -61.01 [-66.47; -55.54] | . |
| **Evolocumab 420mg Q4W** | 1.39 [ -7.26; 10.05] | 17.39 [ 5.79; 28.98] | 9.57 [ 1.85; 17.29] | -2.23 [ -7.67; 3.21] | Evolocumab 420mg Q4W | . | . | . | . | . | . | . | . | . | -59.13 [-63.69; -54.57] | . |
| **Inclisiran 300mg Q3M** | -7.92 [-21.79; 5.95] | 8.07 [ -7.80; 23.95] | 0.26 [-13.05; 13.57] | -11.54 [-24.39; 1.30] | -9.31 [-21.89; 3.26] | Inclisiran 300mg Q3M | . | . | . | . | . | . | . | . | -49.07 [-60.81; -37.33] | . |
| **Lepodisiran 400mg Q6M** | -46.10 [-67.01; -25.18] | -30.10 [-52.39; -7.81] | -37.92 [-58.46; -17.37] | -49.72 [-69.97; -29.47] | -47.49 [-67.56; -27.41] | -38.17 [-60.99; -15.36] | Lepodisiran 400mg Q6M | -4.80 [-24.36; 14.76] | . | . | . | . | . | . | -10.90 [-30.46; 8.66] | . |
| **Lepodisiran 96mg Q6M** | -50.90 [-71.81; -29.98] | -34.90 [-57.19; -12.61] | -42.72 [-63.26; -22.17] | -54.52 [-74.77; -34.27] | -52.29 [-72.36; -32.21] | -42.97 [-65.79; -20.16] | -4.80 [-24.36; 14.76] | Lepodisiran 96mg Q6M | . | . | . | . | . | . | -6.10 [-25.66; 13.46] | . |
| **Muvalaplin 240mg DAILY** | -35.70 [-57.68; -13.71] | -19.70 [-43.00; 3.60] | -27.52 [-49.16; -5.88] | -39.32 [-60.67; -17.96] | -37.09 [-58.28; -15.89] | -27.77 [-51.58; -3.97] | 10.40 [-18.09; 38.89] | 15.20 [-13.29; 43.69] | Muvalaplin 240mg DAILY | -5.30 [-26.01; 15.41] | . | . | . | . | -21.30 [-42.01; -0.59] | . |
| **Muvalaplin 60mg DAILY** | -41.00 [-62.98; -19.01] | -25.00 [-48.30; -1.70] | -32.82 [-54.46; -11.18] | -44.62 [-65.97; -23.26] | -42.39 [-63.58; -21.19] | -33.07 [-56.88; -9.27] | 5.10 [-23.39; 33.59] | 9.90 [-18.59; 38.39] | -5.30 [-26.01; 15.41] | Muvalaplin 60mg DAILY | . | . | . | . | -16.00 [-36.71; 4.71] | . |
| **Olpasiran 225mg Q12W** | -47.77 [-64.24; -31.30] | -31.77 [-49.96; -13.59] | -39.59 [-55.59; -23.60] | -51.39 [-67.01; -35.78] | -49.16 [-64.55; -33.77] | -39.85 [-58.67; -21.02] | -1.67 [-26.16; 22.81] | 3.13 [-21.36; 27.61] | -12.07 [-37.48; 13.33] | -6.77 [-32.18; 18.63] | Olpasiran 225mg Q12W | -2.59 [-17.31; 12.13] | . | . | -9.23 [-23.94; 5.49] | . |
| **Olpasiran 75mg Q12W** | -50.36 [-66.83; -33.90] | -34.37 [-52.55; -16.18] | -42.19 [-58.18; -26.19] | -53.98 [-69.60; -38.37] | -51.75 [-67.14; -36.36] | -42.44 [-61.27; -23.62] | -4.27 [-28.75; 20.21] | 0.53 [-23.95; 25.01] | -14.67 [-40.07; 10.74] | -9.37 [-34.77; 16.04] | -2.59 [-17.31; 12.13] | Olpasiran 75mg Q12W | . | . | -6.63 [-21.35; 8.08] | . |
| **Pelacarsen 200mg QW** | -33.10 [-54.79; -11.40] | -17.10 [-40.13; 5.93] | -24.92 [-46.26; -3.57] | -36.72 [-57.78; -15.66] | -34.49 [-55.38; -13.59] | -25.17 [-48.71; -1.63] | 13.00 [-15.27; 41.27] | 17.80 [-10.47; 46.07] | 2.60 [-26.47; 31.67] | 7.90 [-21.17; 36.97] | 14.67 [-10.48; 39.83] | 17.27 [ -7.89; 42.42] | Pelacarsen 200mg QW | . | -23.90 [-44.30; -3.50] | . |
| **Pelacarsen 80mg QW** | -36.95 [-57.90; -15.99] | -20.95 [-43.28; 1.38] | -28.77 [-49.35; -8.18] | -40.57 [-60.86; -20.28] | -38.34 [-58.46; -18.22] | -29.02 [-51.88; -6.17] | 9.15 [-18.55; 36.85] | 13.95 [-13.75; 41.65] | -1.25 [-29.77; 27.27] | 4.05 [-24.47; 32.57] | 10.82 [-13.69; 35.34] | 13.42 [-11.10; 37.93] | -3.85 [-32.15; 24.45] | Pelacarsen 80mg QW | -20.05 [-39.66; -0.44] | . |
| **Placebo** | -57.00 [-64.38; -49.61] | -41.00 [-51.69; -30.31] | -48.82 [-55.09; -42.55] | -60.62 [-65.84; -55.40] | -58.39 [-62.90; -53.88] | -49.07 [-60.81; -37.33] | -10.90 [-30.46; 8.66] | -6.10 [-25.66; 13.46] | -21.30 [-42.01; -0.59] | -16.00 [-36.71; 4.71] | -9.23 [-23.94; 5.49] | -6.63 [-21.35; 8.08] | -23.90 [-44.30; -3.50] | -20.05 [-39.66; -0.44] | Placebo | 19.49 [ -0.20; 39.17] |
| **Zerlasiran 300mg Q8W** | -37.51 [-58.54; -16.48] | -21.51 [-43.91; 0.89] | -29.33 [-49.99; -8.67] | -41.13 [-61.50; -20.76] | -38.90 [-59.10; -18.70] | -29.59 [-52.51; -6.67] | 8.59 [-19.17; 36.34] | 13.39 [-14.37; 41.14] | -1.81 [-30.39; 26.76] | 3.49 [-25.09; 32.06] | 10.26 [-14.32; 34.84] | 12.85 [-11.73; 37.43] | -4.41 [-32.77; 23.94] | -0.56 [-28.35; 27.22] | 19.49 [ -0.20; 39.17] | Zerlasiran 300mg Q8W |

The network meta-analysis outcomes are detailed in the lower half, while the upper half displays pairwise meta-analysis results. Primary treatments are listed in order of efficacy ranking.

**6.3. Network meta-analysis results for change in TG**

|  | **Alirocumab 150mg Q2W** | **Alirocumab 300mg Q4W** | **Alirocumab 75mg Q2W** | **Evolocumab 140mg Q2W** | **Evolocumab 420mg Q4W** | **Inclisiran 300mg Q3M** | **Lepodisiran 400mg Q6M** | **Lepodisiran 96mg Q6M** | **Muvalaplin 240mg DAILY** | **Muvalaplin 60mg DAILY** | **Pelacarsen 200mg QW** | **Pelacarsen 80mg QW** | **Placebo** |
| --- | --- | --- | --- | --- | --- | --- | --- | --- | --- | --- | --- | --- | --- |
| **Alirocumab 150mg Q2W** | Alirocumab 150mg Q2W | -6.24 [-19.21; 6.73] | -0.36 [ -9.84; 9.12] | . | . | . | . | . | . | . | . | . | -14.43 [-19.10; -9.76] |
| **Alirocumab 300mg Q4W** | -1.86 [ -9.55; 5.83] | Alirocumab 300mg Q4W | -5.50 [-15.23; 4.23] | . | . | . | . | . | . | . | . | . | -12.22 [-20.01; -4.44] |
| **Alirocumab 75mg Q2W** | -5.04 [-10.35; 0.27] | -3.18 [-10.34; 3.99] | Alirocumab 75mg Q2W | . | . | . | . | . | . | . | . | . | -8.79 [-12.39; -5.19] |
| **Evolocumab 140mg Q2W** | 2.51 [ -3.73; 8.76] | 4.38 [ -3.78; 12.54] | 7.55 [ 1.95; 13.16] | Evolocumab 140mg Q2W | -0.69 [ -5.74; 4.37] | . | . | . | . | . | . | . | -16.09 [-20.76; -11.41] |
| **Evolocumab 420mg Q4W** | 1.44 [ -4.04; 6.92] | 3.30 [ -4.29; 10.89] | 6.48 [ 1.74; 11.21] | -1.08 [ -5.56; 3.41] | Evolocumab 420mg Q4W | . | . | . | . | . | . | . | -15.08 [-18.27; -11.89] |
| **Inclisiran 300mg Q3M** | 0.31 [-12.77; 13.39] | 2.17 [-11.92; 16.26] | 5.35 [ -7.43; 18.13] | -2.21 [-15.24; 10.83] | -1.13 [-13.82; 11.56] | Inclisiran 300mg Q3M | . | . | . | . | . | . | -14.30 [-26.58; -2.02] |
| **Lepodisiran 400mg Q6M** | -7.59 [-21.84; 6.66] | -5.73 [-20.91; 9.45] | -2.55 [-16.53; 11.42] | -10.11 [-24.31; 4.10] | -9.03 [-22.92; 4.86] | -7.90 [-26.17; 10.37] | Lepodisiran 400mg Q6M | -3.70 [-17.22; 9.82] | . | . | . | . | -6.40 [-19.92; 7.12] |
| **Lepodisiran 96mg Q6M** | -11.29 [-25.54; 2.96] | -9.43 [-24.61; 5.75] | -6.25 [-20.23; 7.72] | -13.81 [-28.01; 0.40] | -12.73 [-26.62; 1.16] | -11.60 [-29.87; 6.67] | -3.70 [-17.22; 9.82] | Lepodisiran 96mg Q6M | . | . | . | . | -2.70 [-16.22; 10.82] |
| **Muvalaplin 240mg DAILY** | -26.49 [-41.50; -11.48] | -24.63 [-40.53; -8.72] | -21.45 [-36.21; -6.69] | -29.01 [-43.98; -14.03] | -27.93 [-42.61; -13.25] | -26.80 [-45.68; -7.92] | -18.90 [-38.60; 0.80] | -15.20 [-34.90; 4.50] | Muvalaplin 240mg DAILY | 9.60 [ -4.73; 23.93] | . | . | 12.50 [ -1.83; 26.83] |
| **Muvalaplin 60mg DAILY** | -16.89 [-31.90; -1.88] | -15.03 [-30.93; 0.88] | -11.85 [-26.61; 2.91] | -19.41 [-34.38; -4.43] | -18.33 [-33.01; -3.65] | -17.20 [-36.08; 1.68] | -9.30 [-29.00; 10.40] | -5.60 [-25.30; 14.10] | 9.60 [ -4.73; 23.93] | Muvalaplin 60mg DAILY | . | . | 2.90 [-11.43; 17.23] |
| **Pelacarsen 200mg QW** | -3.99 [-28.47; 20.49] | -2.13 [-27.17; 22.91] | 1.05 [-23.28; 25.37] | -6.51 [-30.97; 17.95] | -5.43 [-29.71; 18.85] | -4.30 [-31.32; 22.72] | 3.60 [-24.01; 31.21] | 7.30 [-20.31; 34.91] | 22.50 [ -5.51; 50.51] | 12.90 [-15.11; 40.91] | Pelacarsen 200mg QW | . | -10.00 [-34.07; 14.07] |
| **Pelacarsen 80mg QW** | -7.14 [-21.60; 7.32] | -5.28 [-20.66; 10.10] | -2.10 [-16.29; 12.09] | -9.66 [-24.08; 4.77] | -8.58 [-22.69; 5.53] | -7.45 [-25.89; 10.99] | 0.45 [-18.83; 19.73] | 4.15 [-15.13; 23.43] | 19.35 [ -0.51; 39.21] | 9.75 [-10.11; 29.61] | -3.15 [-30.87; 24.57] | Pelacarsen 80mg QW | -6.85 [-20.60; 6.90] |
| **Placebo** | -13.99 [-18.47; -9.51] | -12.13 [-19.03; -5.23] | -8.95 [-12.47; -5.43] | -16.51 [-20.86; -12.15] | -15.43 [-18.59; -12.26] | -14.30 [-26.58; -2.02] | -6.40 [-19.92; 7.12] | -2.70 [-16.22; 10.82] | 12.50 [ -1.83; 26.83] | 2.90 [-11.43; 17.23] | -10.00 [-34.07; 14.07] | -6.85 [-20.60; 6.90] | Placebo |

The network meta-analysis outcomes are detailed in the lower half, while the upper half displays pairwise meta-analysis results. Primary treatments are listed in order of efficacy ranking.

**6.4. Network meta-analysis results for change in** **Total Cholesterol**

|  | **Alirocumab 150mg Q2W** | **Alirocumab 300mg Q4W** | **Alirocumab 75mg Q2W** | **Evolocumab 140mg Q2W** | **Evolocumab 420mg Q4W** | **Inclisiran 300mg Q3M** | **Lepodisiran 400mg Q6M** | **Lepodisiran 96mg Q6M** | **Muvalaplin 240mg DAILY** | **Muvalaplin 60mg DAILY** | **Pelacarsen 200mg QW** | **Pelacarsen 80mg QW** | **Placebo** | **Zerlasiran 300mg Q8W** |
| --- | --- | --- | --- | --- | --- | --- | --- | --- | --- | --- | --- | --- | --- | --- |
| **Alirocumab 150mg Q2W** | Alirocumab 150mg Q2W | -16.53 [-32.97; -0.09] | -1.73 [-17.22; 13.76] | -38.40 [-60.10; -16.70] | . | . | . | . | . | . | . | . | -37.06 [-44.82; -29.31] | . |
| **Alirocumab 300mg Q4W** | -13.24 [-25.61; -0.87] | Alirocumab 300mg Q4W | -3.40 [-25.39; 18.59] | . | . | . | . | . | . | . | . | . | -26.72 [-39.89; -13.55] | . |
| **Alirocumab 75mg Q2W** | -8.13 [-17.85; 1.59] | 5.11 [ -8.05; 18.26] | Alirocumab 75mg Q2W | . | . | . | . | . | . | . | . | . | -32.52 [-40.87; -24.16] | . |
| **Evolocumab 140mg Q2W** | -6.68 [-16.22; 2.85] | 6.55 [ -7.07; 20.18] | 1.45 [ -9.18; 12.07] | Evolocumab 140mg Q2W | -1.19 [-10.26; 7.87] | . | . | . | . | . | . | . | -38.71 [-46.68; -30.74] | . |
| **Evolocumab 420mg Q4W** | -5.61 [-14.80; 3.57] | 7.63 [ -5.57; 20.82] | 2.52 [ -7.48; 12.51] | 1.07 [ -6.69; 8.84] | Evolocumab 420mg Q4W | . | . | . | . | . | . | . | -36.48 [-42.65; -30.31] | . |
| **Inclisiran 300mg Q3M** | -12.94 [-28.47; 2.60] | 0.30 [-17.84; 18.44] | -4.81 [-20.76; 11.15] | -6.25 [-21.80; 9.29] | -7.32 [-22.40; 7.75] | Inclisiran 300mg Q3M | . | . | . | . | . | . | -27.96 [-41.76; -14.15] | . |
| **Lepodisiran 400mg Q6M** | -33.39 [-56.70; -10.09] | -20.16 [-45.27; 4.96] | -25.26 [-48.85; -1.68] | -26.71 [-50.02; -3.40] | -27.78 [-50.78; -4.78] | -20.46 [-46.59; 5.68] | Lepodisiran 400mg Q6M | -4.60 [-26.79; 17.59] | . | . | . | . | -7.50 [-29.69; 14.69] | . |
| **Lepodisiran 96mg Q6M** | -37.99 [-61.30; -14.69] | -24.76 [-49.87; 0.36] | -29.86 [-53.45; -6.28] | -31.31 [-54.62; -8.00] | -32.38 [-55.38; -9.38] | -25.06 [-51.19; 1.08] | -4.60 [-26.79; 17.59] | Lepodisiran 96mg Q6M | . | . | . | . | -2.90 [-25.09; 19.29] | . |
| **Muvalaplin 240mg DAILY** | -31.19 [-54.82; -7.56] | -17.96 [-43.38; 7.46] | -23.06 [-46.97; 0.85] | -24.51 [-48.15; -0.87] | -25.58 [-48.91; -2.25] | -18.26 [-44.68; 8.17] | 2.20 [-29.42; 33.82] | 6.80 [-24.82; 38.42] | Muvalaplin 240mg DAILY | -2.50 [-25.03; 20.03] | . | . | -9.70 [-32.23; 12.83] | . |
| **Muvalaplin 60mg DAILY** | -33.69 [-57.32; -10.06] | -20.46 [-45.88; 4.96] | -25.56 [-49.47; -1.65] | -27.01 [-50.65; -3.37] | -28.08 [-51.41; -4.75] | -20.76 [-47.18; 5.67] | -0.30 [-31.92; 31.32] | 4.30 [-27.32; 35.92] | -2.50 [-25.03; 20.03] | Muvalaplin 60mg DAILY | . | . | -7.20 [-29.73; 15.33] | . |
| **Pelacarsen 200mg QW** | -23.89 [-47.85; 0.07] | -10.66 [-36.38; 15.07] | -15.76 [-39.99; 8.47] | -17.21 [-41.18; 6.76] | -18.28 [-41.94; 5.38] | -10.96 [-37.67; 15.76] | 9.50 [-22.37; 41.37] | 14.10 [-17.77; 45.97] | 7.30 [-24.81; 39.41] | 9.80 [-22.31; 41.91] | Pelacarsen 200mg QW | . | -17.00 [-39.88; 5.88] | . |
| **Pelacarsen 80mg QW** | -35.91 [-59.36; -12.47] | -22.68 [-47.92; 2.57] | -27.78 [-51.51; -4.06] | -29.23 [-52.68; -5.78] | -30.30 [-53.44; -7.16] | -22.98 [-49.24; 3.28] | -2.52 [-34.01; 28.97] | 2.08 [-29.41; 33.57] | -4.72 [-36.45; 27.01] | -2.22 [-33.95; 29.51] | -12.02 [-43.99; 19.95] | Pelacarsen 80mg QW | -4.98 [-27.32; 17.36] | . |
| **Placebo** | -40.89 [-48.02; -33.77] | -27.66 [-39.42; -15.89] | -32.76 [-40.76; -24.77] | -34.21 [-41.36; -27.06] | -35.28 [-41.33; -29.23] | -27.96 [-41.76; -14.15] | -7.50 [-29.69; 14.69] | -2.90 [-25.09; 19.29] | -9.70 [-32.23; 12.83] | -7.20 [-29.73; 15.33] | -17.00 [-39.88; 5.88] | -4.98 [-27.32; 17.36] | Placebo | 9.00 [-17.71; 35.71] |
| **Zerlasiran 300mg Q8W** | -31.89 [-59.54; -4.25] | -18.66 [-47.85; 10.53] | -23.76 [-51.65; 4.12] | -25.21 [-52.86; 2.44] | -26.28 [-53.67; 1.11] | -18.96 [-49.03; 11.11] | 1.50 [-33.23; 36.23] | 6.10 [-28.63; 40.83] | -0.70 [-35.65; 34.25] | 1.80 [-33.15; 36.75] | -8.00 [-43.17; 27.17] | 4.02 [-30.80; 38.84] | 9.00 [-17.71; 35.71] | Zerlasiran 300mg Q8W |

The network meta-analysis outcomes are detailed in the lower half, while the upper half displays pairwise meta-analysis results. Primary treatments are listed in order of efficacy ranking.

**6.5. Network meta-analysis results for change in HDL-C**

|  | **Alirocumab 150mg Q2W** | **Alirocumab 300mg Q4W** | **Alirocumab 75mg Q2W** | **Evolocumab 140mg Q2W** | **Evolocumab 420mg Q4W** | **Inclisiran 300mg Q3M** | **Lepodisiran 400mg Q6M** | **Lepodisiran 96mg Q6M** | **Muvalaplin 240mg DAILY** | **Muvalaplin 60mg DAILY** | **Pelacarsen 200mg QW** | **Pelacarsen 80mg QW** | **Placebo** |
| --- | --- | --- | --- | --- | --- | --- | --- | --- | --- | --- | --- | --- | --- |
| **Alirocumab 150mg Q2W** | **Alirocumab 150mg Q2W** | **-1.26 [ -7.21; 4.70]** | **0.30 [ -5.37; 5.97]** | **.** | **.** | **.** | **.** | **.** | **.** | **.** | **.** | **.** | **5.75 [ 3.87; 7.64]** |
| **Alirocumab 300mg Q4W** | **-0.68 [ -4.34; 2.99]** | **Alirocumab 300mg Q4W** | **.** | **.** | **.** | **.** | **.** | **.** | **.** | **.** | **.** | **.** | **6.47 [ 2.90; 10.03]** |
| **Alirocumab 75mg Q2W** | **-1.51 [ -4.16; 1.14]** | **-0.84 [ -4.79; 3.12]** | **Alirocumab 75mg Q2W** | **.** | **.** | **.** | **.** | **.** | **.** | **.** | **.** | **.** | **7.42 [ 5.32; 9.52]** |
| **Evolocumab 140mg Q2W** | **-1.13 [ -3.83; 1.56]** | **-0.45 [ -4.38; 3.47]** | **0.38 [ -2.47; 3.23]** | **Evolocumab 140mg Q2W** | **-0.52 [ -2.80; 1.76]** | **.** | **.** | **.** | **.** | **.** | **.** | **.** | **7.32 [ 5.20; 9.45]** |
| **Evolocumab 420mg Q4W** | **-1.51 [ -3.82; 0.80]** | **-0.83 [ -4.50; 2.84]** | **0.01 [ -2.48; 2.49]** | **-0.38 [ -2.40; 1.65]** | **Evolocumab 420mg Q4W** | **.** | **.** | **.** | **.** | **.** | **.** | **.** | **7.32 [ 5.92; 8.73]** |
| **Inclisiran 300mg Q3M** | **-1.26 [ -6.17; 3.65]** | **-0.58 [ -6.26; 5.10]** | **0.26 [ -4.74; 5.25]** | **-0.13 [ -5.09; 4.84]** | **0.25 [ -4.52; 5.01]** | **Inclisiran 300mg Q3M** | **.** | **.** | **.** | **.** | **.** | **.** | **6.88 [ 2.32; 11.43]** |
| **Lepodisiran 400mg Q6M** | **5.82 [ 0.10; 11.53]** | **6.49 [ 0.11; 12.88]** | **7.33 [ 1.54; 13.12]** | **6.95 [ 1.19; 12.71]** | **7.32 [ 1.74; 12.91]** | **7.08 [ 0.00; 14.15]** | **Lepodisiran 400mg Q6M** | **-0.70 [ -6.05; 4.65]** | **.** | **.** | **.** | **.** | **-0.20 [ -5.61; 5.21]** |
| **Lepodisiran 96mg Q6M** | **5.12 [ -0.69; 10.92]** | **5.79 [ -0.67; 12.26]** | **6.63 [ 0.75; 12.51]** | **6.25 [ 0.40; 12.10]** | **6.62 [ 0.95; 12.30]** | **6.38 [ -0.77; 13.52]** | **-0.70 [ -6.05; 4.65]** | **Lepodisiran 96mg Q6M** | **.** | **.** | **.** | **.** | **0.50 [ -5.00; 6.00]** |
| **Muvalaplin 240mg DAILY** | **11.62 [ 5.81; 17.42]** | **12.29 [ 5.83; 18.76]** | **13.13 [ 7.25; 19.01]** | **12.75 [ 6.90; 18.60]** | **13.12 [ 7.45; 18.80]** | **12.88 [ 5.73; 20.02]** | **5.80 [ -1.92; 13.52]** | **6.50 [ -1.28; 14.28]** | **Muvalaplin 240mg DAILY** | **-2.10 [ -7.60; 3.40]** | **.** | **.** | **-6.00 [-11.50; -0.50]** |
| **Muvalaplin 60mg DAILY** | **9.52 [ 3.71; 15.32]** | **10.19 [ 3.73; 16.66]** | **11.03 [ 5.15; 16.91]** | **10.65 [ 4.80; 16.50]** | **11.02 [ 5.35; 16.70]** | **10.78 [ 3.63; 17.92]** | **3.70 [ -4.02; 11.42]** | **4.40 [ -3.38; 12.18]** | **-2.10 [ -7.60; 3.40]** | **Muvalaplin 60mg DAILY** | **.** | **.** | **-3.90 [ -9.40; 1.60]** |
| **Pelacarsen 200mg QW** | **13.32 [ 2.67; 23.96]** | **13.99 [ 2.98; 25.01]** | **14.83 [ 4.15; 25.51]** | **14.45 [ 3.78; 25.12]** | **14.82 [ 4.25; 25.40]** | **14.58 [ 3.14; 26.01]** | **7.50 [ -4.30; 19.30]** | **8.20 [ -3.64; 20.04]** | **1.70 [-10.14; 13.54]** | **3.80 [ -8.04; 15.64]** | **Pelacarsen 200mg QW** | **.** | **-7.70 [-18.18; 2.78]** |
| **Pelacarsen 80mg QW** | **-0.76 [ -7.97; 6.44]** | **-0.09 [ -7.84; 7.67]** | **0.75 [ -6.52; 8.02]** | **0.37 [ -6.87; 7.61]** | **0.74 [ -6.36; 7.85]** | **0.50 [ -7.83; 8.82]** | **-6.58 [-15.40; 2.24]** | **-5.88 [-14.76; 3.00]** | **-12.38 [-21.26; -3.50]** | **-10.28 [-19.16; -1.40]** | **-14.08 [-26.67; -1.49]** | **Pelacarsen 80mg QW** | **6.38 [ -0.59; 13.35]** |
| **Placebo** | **5.62 [ 3.78; 7.45]** | **6.29 [ 2.90; 9.69]** | **7.13 [ 5.07; 9.19]** | **6.75 [ 4.78; 8.72]** | **7.12 [ 5.73; 8.52]** | **6.88 [ 2.32; 11.43]** | **-0.20 [ -5.61; 5.21]** | **0.50 [ -5.00; 6.00]** | **-6.00 [-11.50; -0.50]** | **-3.90 [ -9.40; 1.60]** | **-7.70 [-18.18; 2.78]** | **6.38 [ -0.59; 13.35]** | **Placebo** |

The network meta-analysis outcomes are detailed in the lower half, while the upper half displays pairwise meta-analysis results. Primary treatments are listed in order of efficacy ranking.

**6.6. Adverse events**

### **6.6.1 Injection site reactions**

|  | **Alirocumab 150mg Q2W** | **Alirocumab 300mg Q4W** | **Alirocumab 75mg Q2W** | **Evolocumab 140mg Q2W** | **Evolocumab 420mg Q4W** | **Inclisiran 300mg Q3M** | **Olpasiran 225mg Q12W** | **Olpasiran 75mg Q12W** | **Placebo** | **Zerlasiran 300 mg Q8W** |
| --- | --- | --- | --- | --- | --- | --- | --- | --- | --- | --- |
| Alirocumab 150mg Q2W | Alirocumab 150mg Q2W | 0.94 [0.43; 2.04] | 3.57 [1.25; 10.16] | . | . | . | . | . | 1.53 [1.10; 2.13] | . |
| Alirocumab 300mg Q4W | 0.84 [0.40; 1.78] | Alirocumab 300mg Q4W | . | . | . | . | . | . | 3.50 [0.86; 14.18] | . |
| Alirocumab 75mg Q2W | 1.26 [0.74; 2.16] | 1.50 [0.61; 3.70] | Alirocumab 75mg Q2W | . | . | . | . | . | 1.69 [1.02; 2.82] | . |
| Evolocumab 140mg Q2W | 1.65 [0.79; 3.41] | 1.95 [0.70; 5.46] | 1.30 [0.58; 2.92] | Evolocumab 140mg Q2W | 1.07 [0.52; 2.22] | . | . | . | 1.10 [0.52; 2.31] | . |
| Evolocumab 420mg Q4W | 1.87 [1.03; 3.38] | 2.21 [0.87; 5.64] | 1.48 [0.74; 2.93] | 1.13 [0.60; 2.15] | Evolocumab 420mg Q4W | . | . | . | 0.83 [0.50; 1.39] | . |
| Inclisiran 300mg Q3M | 3.23 [0.21; 49.83] | 3.84 [0.23; 64.96] | 2.56 [0.16; 40.30] | 1.96 [0.12; 32.10] | 1.73 [0.11; 27.44] | Inclisiran 300mg Q3M | . | . | 0.51 [0.03; 7.76] | . |
| Olpasiran 225mg Q12W | 0.87 [0.35; 2.17] | 1.04 [0.32; 3.32] | 0.69 [0.26; 1.83] | 0.53 [0.18; 1.56] | 0.47 [0.17; 1.26] | 0.27 [0.02; 4.66] | Olpasiran 225mg Q12W | 1.12 [0.55; 2.26] | 1.90 [0.81; 4.46] | . |
| Olpasiran 75mg Q12W | 0.98 [0.39; 2.47] | 1.16 [0.36; 3.76] | 0.77 [0.29; 2.08] | 0.59 [0.20; 1.76] | 0.52 [0.19; 1.43] | 0.30 [0.02; 5.24] | 1.12 [0.55; 2.26] | Olpasiran 75mg Q12W | 1.70 [0.71; 4.06] | . |
| Placebo | 1.66 [1.21; 2.29] | 1.97 [0.89; 4.35] | 1.31 [0.82; 2.11] | 1.01 [0.52; 1.94] | 0.89 [0.54; 1.47] | 0.51 [0.03; 7.76] | 1.90 [0.81; 4.46] | 1.70 [0.71; 4.04] | Placebo | 0.54 [0.14; 2.11] |
| Zerlasiran 300 mg Q8W | 0.89 [0.22; 3.63] | 1.06 [0.22; 5.14] | 0.70 [0.17; 3.00] | 0.54 [0.12; 2.47] | 0.48 [0.11; 2.05] | 0.28 [0.01; 5.77] | 1.02 [0.20; 5.12] | 0.91 [0.18; 4.61] | 0.54 [0.14; 2.11] | Zerlasiran 300 mg Q8W |

The network meta-analysis outcomes are detailed in the lower half, while the upper half displays pairwise meta-analysis results. Primary treatments are listed in order of efficacy ranking.

### **6.6.2** **Serious adverse events**

|  | **Alirocumab 150mg Q2W** | **Alirocumab 300mg Q4W** | **Alirocumab 75mg Q2W** | **Evolocumab 140mg Q2W** | **Evolocumab 420mg Q4W** | **Inclisiran 300mg Q3M** | **Lepodisiran 96mg Q6M** | **Olpasiran 225mg Q12W** | **Olpasiran 75mg Q12W** | **Placebo** | **Zerlasiran 300 mg Q8W** |
| --- | --- | --- | --- | --- | --- | --- | --- | --- | --- | --- | --- |
| **Alirocumab 150mg Q2W** | Alirocumab 150mg Q2W | 0.90 [0.06; 13.77] | 2.31 [0.81; 6.57] | . | . | . | . | . | . | 0.97 [0.83; 1.13] | . |
| **Alirocumab 300mg Q4W** | 1.23 [0.77; 1.96] | Alirocumab 300mg Q4W | 0.94 [0.47; 1.89] | . | . | . | . | . | . | 0.75 [0.46; 1.22] | . |
| **Alirocumab 75mg Q2W** | 1.01 [0.78; 1.30] | 0.82 [0.51; 1.31] | Alirocumab 75mg Q2W | . | . | . | . | . | . | 0.99 [0.80; 1.24] | . |
| **Evolocumab 140mg Q2W** | 0.72 [0.38; 1.37] | 0.58 [0.27; 1.26] | 0.71 [0.37; 1.38] | Evolocumab 140mg Q2W | 1.43 [0.61; 3.33] | . | . | . | . | 1.23 [0.61; 2.49] | . |
| **Evolocumab 420mg Q4W** | 0.86 [0.55; 1.35] | 0.70 [0.38; 1.30] | 0.86 [0.53; 1.38] | 1.20 [0.62; 2.31] | Evolocumab 420mg Q4W | . | . | . | . | 1.13 [0.73; 1.75] | . |
| **Inclisiran 300mg Q3M** | 0.95 [0.09; 9.88] | 0.78 [0.07; 8.37] | 0.95 [0.09; 9.88] | 1.33 [0.12; 14.90] | 1.11 [0.10; 11.89] | Inclisiran 300mg Q3M | . | . | . | 1.03 [0.10; 10.62] | . |
| **Lepodisiran 96mg Q6M** | 1.95 [0.15; 26.23] | 1.59 [0.11; 22.14] | 1.94 [0.14; 26.21] | 2.73 [0.19; 39.27] | 2.27 [0.16; 31.46] | 2.05 [0.06; 67.33] | Lepodisiran 96mg Q6M | . | . | 0.50 [0.04; 6.68] | . |
| **Olpasiran 225mg Q12W** | 1.23 [0.48; 3.14] | 1.01 [0.36; 2.81] | 1.23 [0.47; 3.17] | 1.72 [0.56; 5.25] | 1.43 [0.52; 3.97] | 1.30 [0.11; 15.98] | 0.63 [0.04; 9.89] | Olpasiran 225mg Q12W | 1.77 [0.54; 5.76] | 0.81 [0.32; 2.03] | . |
| **Olpasiran 75mg Q12W** | 2.07 [0.66; 6.50] | 1.69 [0.50; 5.72] | 2.06 [0.65; 6.53] | 2.89 [0.79; 10.56] | 2.41 [0.72; 8.09] | 2.18 [0.16; 29.23] | 1.06 [0.06; 17.97] | 1.68 [0.52; 5.46] | Olpasiran 75mg Q12W | 0.47 [0.15; 1.49] | . |
| **Placebo** | 0.98 [0.84; 1.13] | 0.80 [0.51; 1.25] | 0.97 [0.78; 1.21] | 1.36 [0.73; 2.55] | 1.14 [0.74; 1.74] | 1.03 [0.10; 10.62] | 0.50 [0.04; 6.68] | 0.79 [0.31; 2.00] | 0.47 [0.15; 1.46] | Placebo | 1.87 [0.36; 9.72] |
| **Zerlasiran 300 mg Q8W** | 1.83 [0.35; 9.56] | 1.49 [0.27; 8.22] | 1.82 [0.35; 9.58] | 2.55 [0.44; 14.87] | 2.13 [0.39; 11.66] | 1.92 [0.11; 33.51] | 0.94 [0.04; 20.20] | 1.48 [0.22; 9.80] | 0.88 [0.12; 6.51] | 1.87 [0.36; 9.72] | Zerlasiran 300 mg Q8W |

The network meta-analysis outcomes are detailed in the lower half, while the upper half displays pairwise meta-analysis results. Primary treatments are listed in order of efficacy ranking.

### **6.6.3 Treatment discontinuation due to adverse events**

|  | **Alirocumab 150mg Q2W** | **Alirocumab 300mg Q4W** | **Alirocumab 75mg Q2W** | **Evolocumab 140mg Q2W** | **Evolocumab 420mg Q4W** | **Inclisiran 300mg Q3M** | **Olpasiran 225mg Q12W** | **Olpasiran 75mg Q12W** | **Pelacarsen 80mg QW** | **Placebo** | **Zerlasiran 300 mg Q8W** |
| --- | --- | --- | --- | --- | --- | --- | --- | --- | --- | --- | --- |
| **Alirocumab 150mg Q2W** | Alirocumab 150mg Q2W | 0.69 [0.16; 2.98] | 3.58 [0.95; 13.51] | . | . | . | . | . | . | 1.24 [0.92; 1.68] | . |
| **Alirocumab 300mg Q4W** | 1.43 [0.76; 2.71] | Alirocumab 300mg Q4W | 1.06 [0.37; 3.07] | . | . | . | . | . | . | 0.79 [0.41; 1.49] | . |
| **Alirocumab 75mg Q2W** | 1.48 [0.88; 2.47] | 1.03 [0.52; 2.04] | Alirocumab 75mg Q2W | . | . | . | . | . | . | 0.88 [0.56; 1.38] | . |
| **Evolocumab 140mg Q2W** | 1.49 [0.63; 3.52] | 1.04 [0.38; 2.82] | 1.01 [0.40; 2.53] | Evolocumab 140mg Q2W | 1.12 [0.46; 2.72] | . | . | . | . | 0.81 [0.33; 1.99] | . |
| **Evolocumab 420mg Q4W** | 1.56 [0.79; 3.10] | 1.09 [0.47; 2.56] | 1.06 [0.50; 2.26] | 1.05 [0.47; 2.35] | Evolocumab 420mg Q4W | . | . | . | . | 0.81 [0.43; 1.50] | . |
| **Inclisiran 300mg Q3M** | 2.42 [0.16; 37.18] | 1.69 [0.10; 27.19] | 1.64 [0.10; 25.66] | 1.63 [0.10; 27.68] | 1.55 [0.10; 25.06] | Inclisiran 300mg Q3M | . | . | . | 0.51 [0.03; 7.76] | . |
| **Olpasiran 225mg Q12W** | 1.29 [0.08; 20.41] | 0.90 [0.05; 14.91] | 0.87 [0.05; 14.08] | 0.87 [0.05; 15.18] | 0.82 [0.05; 13.75] | 0.53 [0.01; 25.34] | Olpasiran 225mg Q12W | 1.04 [0.07; 16.16] | . | 0.96 [0.06; 15.03] | . |
| **Olpasiran 75mg Q12W** | 1.33 [0.08; 21.15] | 0.93 [0.06; 15.45] | 0.90 [0.06; 14.59] | 0.90 [0.05; 15.72] | 0.85 [0.05; 14.24] | 0.55 [0.01; 26.26] | 1.04 [0.07; 16.16] | Olpasiran 75mg Q12W | . | 0.93 [0.06; 14.52] | . |
| **Pelacarsen 80mg QW** | 4.35 [0.43; 43.48] | 3.04 [0.29; 32.06] | 2.94 [0.29; 30.12] | 2.92 [0.26; 32.96] | 2.78 [0.26; 29.59] | 1.80 [0.05; 62.46] | 3.37 [0.09; 120.06] | 3.26 [0.09; 115.96] | Pelacarsen 80mg QW | 0.29 [0.03; 2.80] | . |
| **Placebo** | 1.24 [0.92; 1.67] | 0.87 [0.48; 1.56] | 0.84 [0.54; 1.31] | 0.84 [0.37; 1.88] | 0.79 [0.43; 1.47] | 0.51 [0.03; 7.76] | 0.96 [0.06; 15.03] | 0.93 [0.06; 14.52] | 0.29 [0.03; 2.80] | Placebo | 0.94 [0.06; 14.52] |
| **Zerlasiran 300 mg Q8W** | 1.16 [0.07; 18.33] | 0.81 [0.05; 13.39] | 0.79 [0.05; 12.65] | 0.78 [0.04; 13.63] | 0.74 [0.04; 12.35] | 0.48 [0.01; 22.79] | 0.90 [0.02; 43.73] | 0.87 [0.02; 42.23] | 0.27 [0.01; 9.48] | 0.94 [0.06; 14.52] | Zerlasiran 300 mg Q8W |

The network meta-analysis outcomes are detailed in the lower half, while the upper half displays pairwise meta-analysis results. Primary treatments are listed in order of efficacy ranking.

**Supplement 7. Sensitivity Analysis**

To evaluate the robustness of the network meta-analysis findings, we conducted prespecified sensitivity analyses for the primary outcome and all secondary outcomes. These included sequential leave-one-out analyses (iteratively excluding one trial at a time), exclusion of trials at high risk of bias, restriction to larger studies, and re-estimation under a common-effects (fixed-effect) framework. These analyses were intended to determine whether individual trials or model assumptions unduly influenced pooled estimates and treatment rankings, and to assess whether heterogeneity and any inconsistency signals persisted under alternative analytic scenarios.

- **Primary outcome (Lp(a)):** Findings for placebo-adjusted percent change in Lp(a) were stable across sensitivity analyses. In the main random-effects model, heterogeneity was considerable (I² = 90.9% [95% CI 88.8–92.5%]; τ² = 119.16). Nevertheless, treatment effects and rankings remained directionally consistent across exclusion of high-risk-of-bias trials (k = 50), restriction to larger studies (k = 29), common-effects modeling, and leave-one-out analyses. Heterogeneity persisted after exclusion of high-risk-of-bias trials (I² ≈ 91%; τ² ≈ 120.5), indicating that variability reflects underlying clinical and methodological diversity rather than the influence of a single outlying trial. Inconsistency assessments showed evidence of between-design inconsistency in the high-risk-of-bias exclusion scenario (Q = 103.45, df = 8, p < 0.001), whereas no between-design inconsistency was detected after restricting to larger studies (Q = 2.95, df = 2, p = 0.229).
- **Secondary efficacy outcomes (LDL-C, total cholesterol, triglycerides, HDL-C).** Sensitivity analyses similarly supported the robustness of secondary lipid findings. For LDL-C, between-study heterogeneity remained substantial in the main analysis (I² = 87.6% [95% CI 84.6–90.1%]; τ² = 80.27), but exclusion of high-risk-of-bias trials (k = 47), restriction to larger studies (k = 29), common-effects modeling, and leave-one-out analyses did not materially alter the treatment hierarchy or principal conclusions. For total cholesterol, treatment rankings and effect estimates were broadly preserved after excluding high-risk-of-bias trials (k = 38) and after restriction to larger studies (k = 22); leave-one-out analyses showed stable heterogeneity estimates (I² ≈ 80–81%; τ² ≈ 24–26). For triglycerides, heterogeneity was moderate in the main analysis (I² = 42.7% [95% CI 17.2–60.4%]; τ² = 13.72), and leave-one-out analyses confirmed that no single trial disproportionately influenced the results (I² ≈ 34–44% across iterations), with conclusions preserved under exclusion of high-risk-of-bias studies (k = 39) and restriction to larger studies (k = 25). For HDL-C, heterogeneity was low to moderate (I² = 33.1% [95% CI 2.5–54.0%]; τ² = 2.60); sensitivity analyses remained directionally consistent, although pelacarsen 80 mg became nominally significant under the common-effects assumption, which was not supported in random-effects analyses and should be interpreted cautiously.
- **Safety outcomes (injection-site reactions, serious adverse events, discontinuation due to adverse events).** Safety findings were generally robust across sensitivity analyses, with low heterogeneity and limited evidence of inconsistency. For injection-site reactions, heterogeneity and inconsistency were negligible in the main analysis (I² = 0.0% [0.0–43.9%]; τ² = 0), and the overall pattern remained stable across leave-one-out analyses, exclusion of high-risk-of-bias studies (I² = 0.0% [0.0–45.4%]; τ² = 0), and restriction to larger studies (I² = 12.4%; τ² = 0.0545). For serious adverse events, heterogeneity was negligible (I² = 0.0% [0.0–38.7%]; τ² = 0.000) with no evidence of global inconsistency (Q = 21.94, df = 33, p = 0.929); however, estimates were imprecise due to low event counts. For discontinuation due to adverse events, heterogeneity and inconsistency were negligible (I² = 0%; τ² = 0), and sensitivity analyses excluding high-risk-of-bias and small-sample studies produced highly consistent estimates; leave-one-out analyses did not materially change pooled estimates.
- **Conclusion of sensitivity analyses.** Across the expanded evidence base comparing emerging Lp(a)-lowering therapies and PCSK9-pathway inhibitors, sequential leave-one-out analyses, risk-of-bias restrictions, large-study restrictions, and alternative model assumptions yielded directionally consistent results for both efficacy and safety outcomes. Persistent heterogeneity, particularly for Lp(a) and LDL-C, appears to be an intrinsic feature of the evidence base rather than dependence on any single study.

# **Supplement 8. Supplementary Results and Exploratory Heterogeneity Analyses**

## **8.1.** **Exploratory heterogeneity analyses for the primary Lp(a) outcome**

### **8.1.1. Phase-stratified analyses**

Phase-stratified analyses were performed for the primary Lp(a) outcome among phase 2 and phase 3 trials. Phase 1 and phase 4 trials were not analyzed separately because of the limited number of studies and sparse network structure.
**Supplementary Figure S8.1.** Phase 2 network plot for the primary Lp(a) outcome.


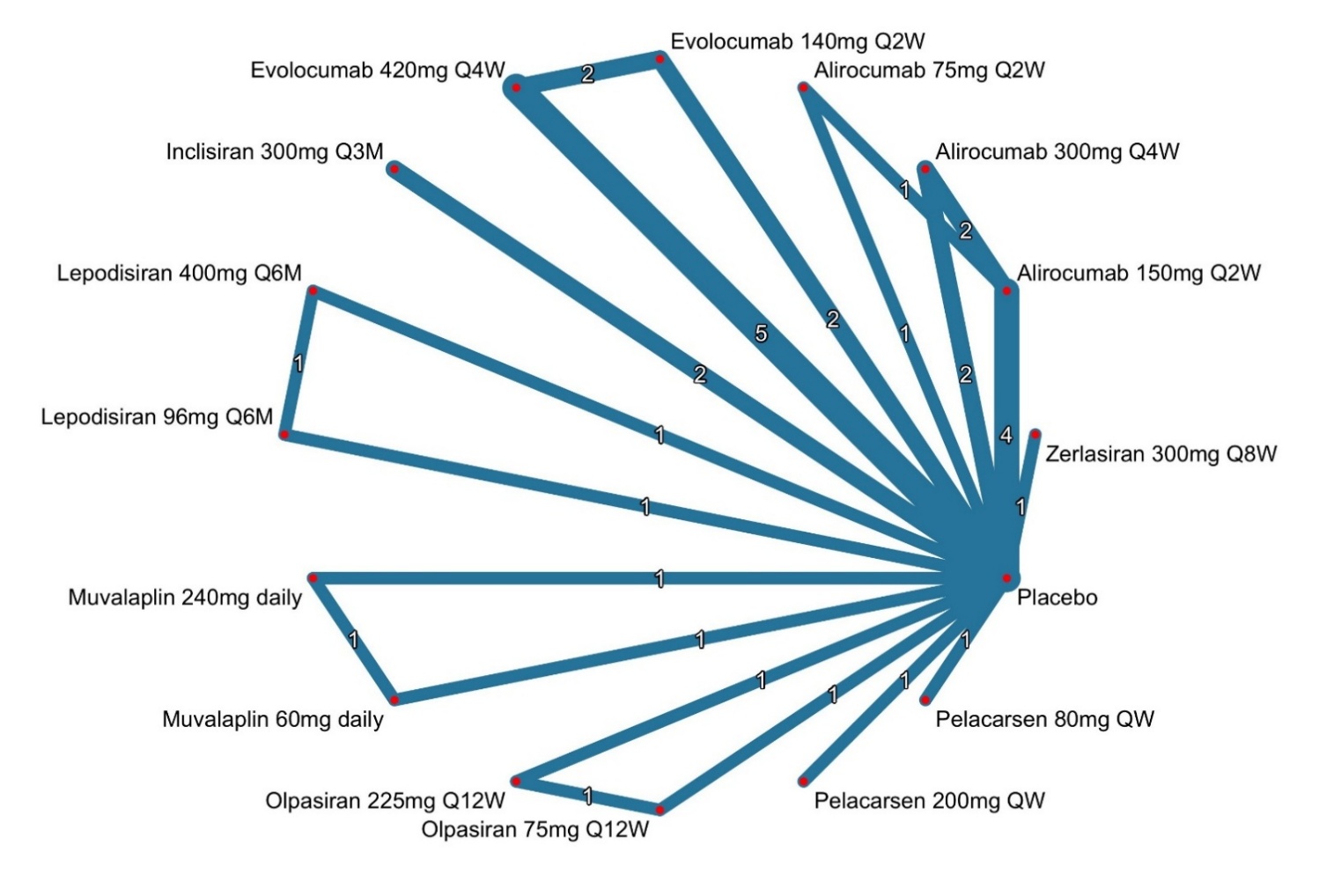


**Supplementary Figure S8.2.** Phase 2 forest plot for the primary Lp(a) outcome.

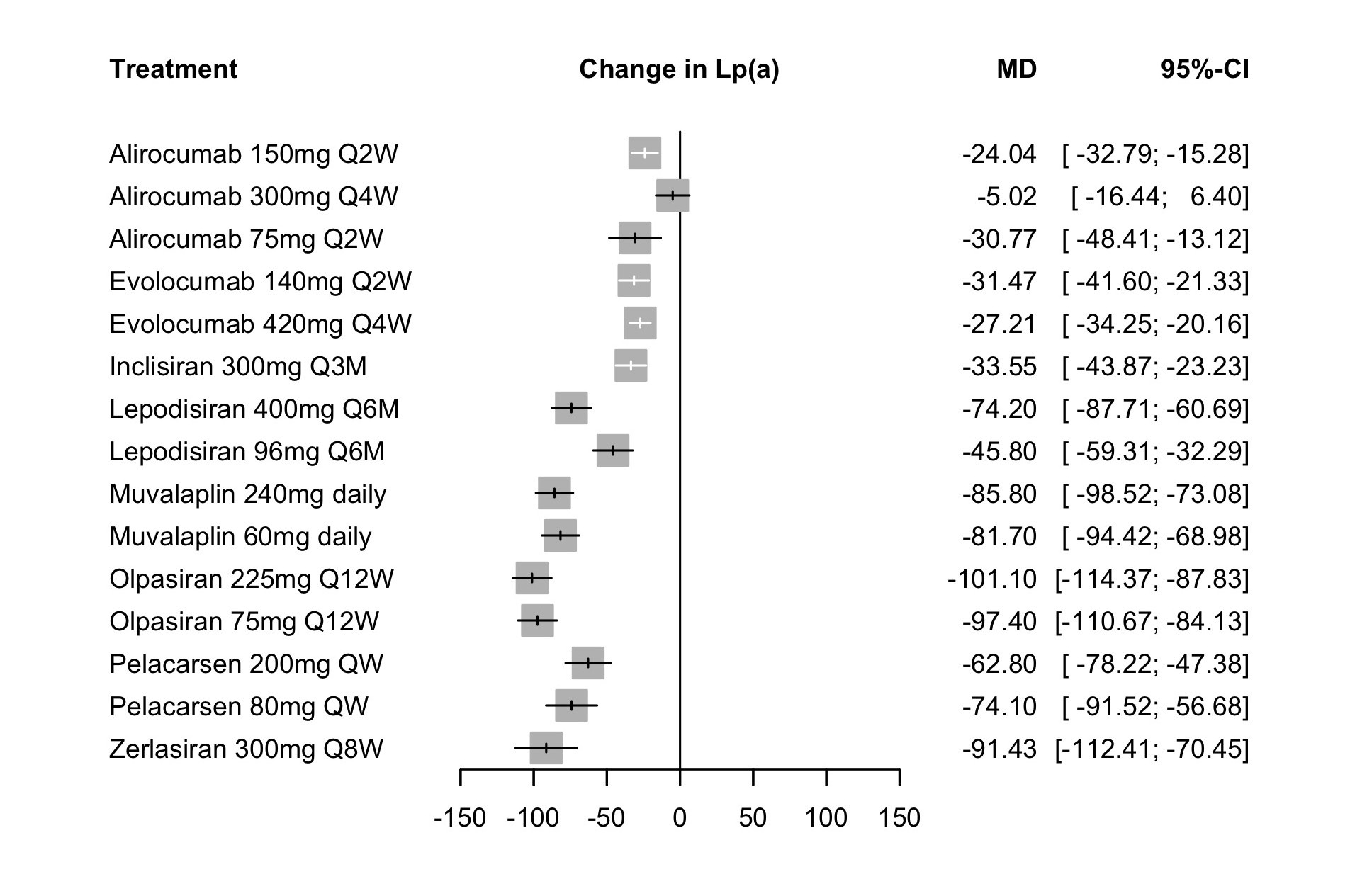


**Supplementary Figure S8.3.** Phase 2 funnel plot for the primary Lp(a) outcome.
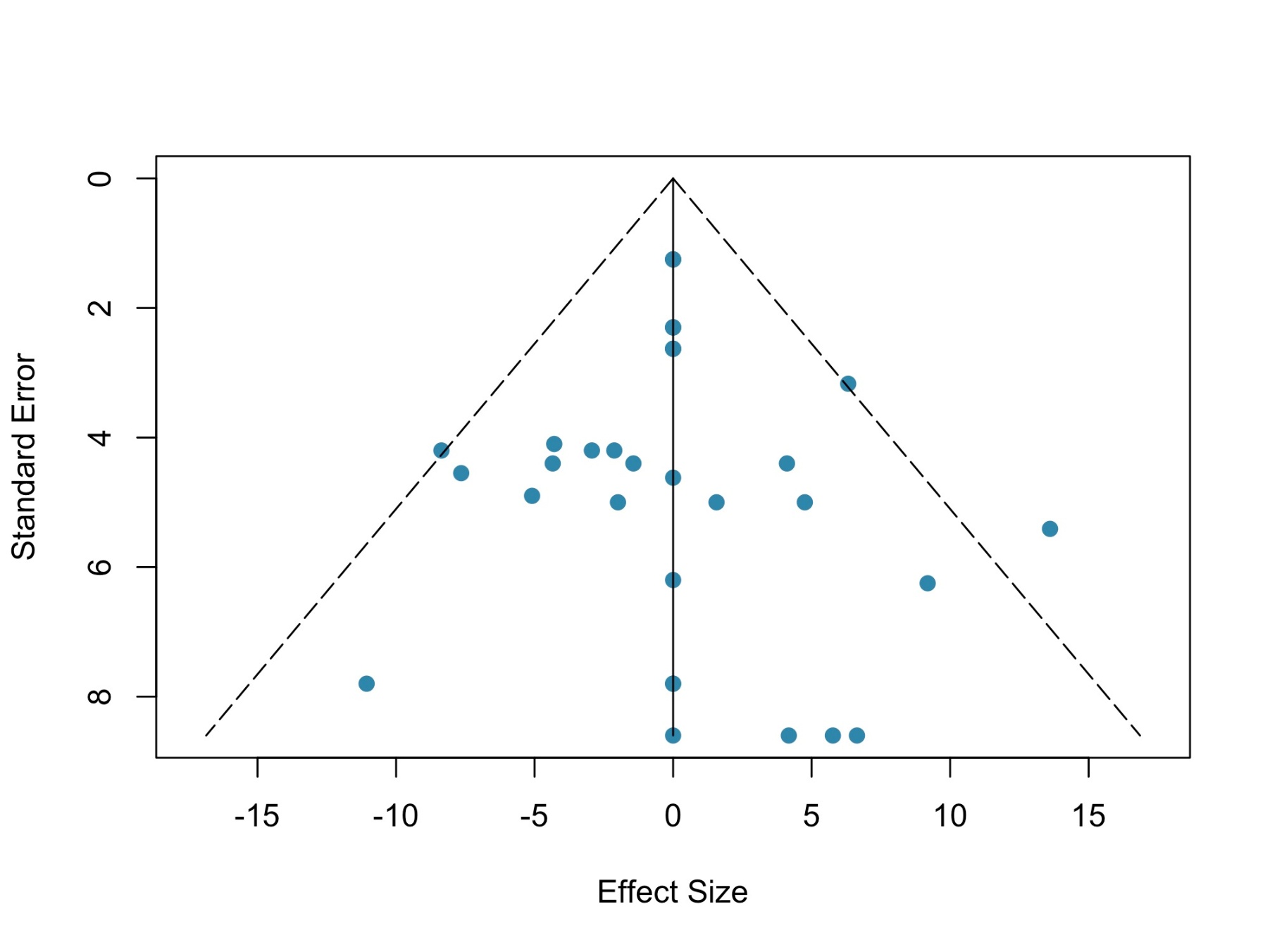
 **Supplementary Figure S8.4.** Phase 3 network plot for the primary Lp(a) outcome.
**
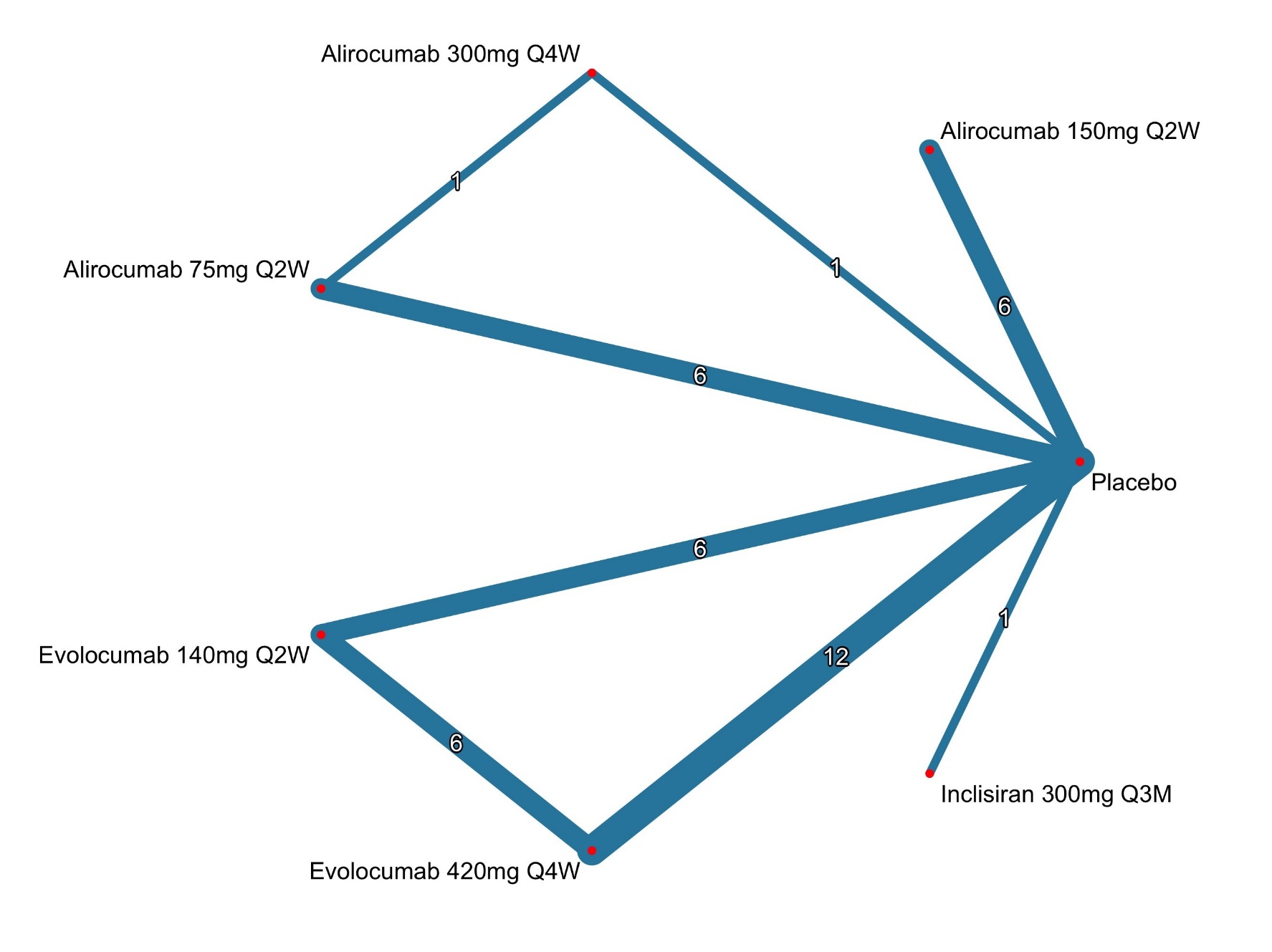
**

**Supplementary Figure S8.5.** Phase 3 forest plot for the primary Lp(a) outcome.

 **
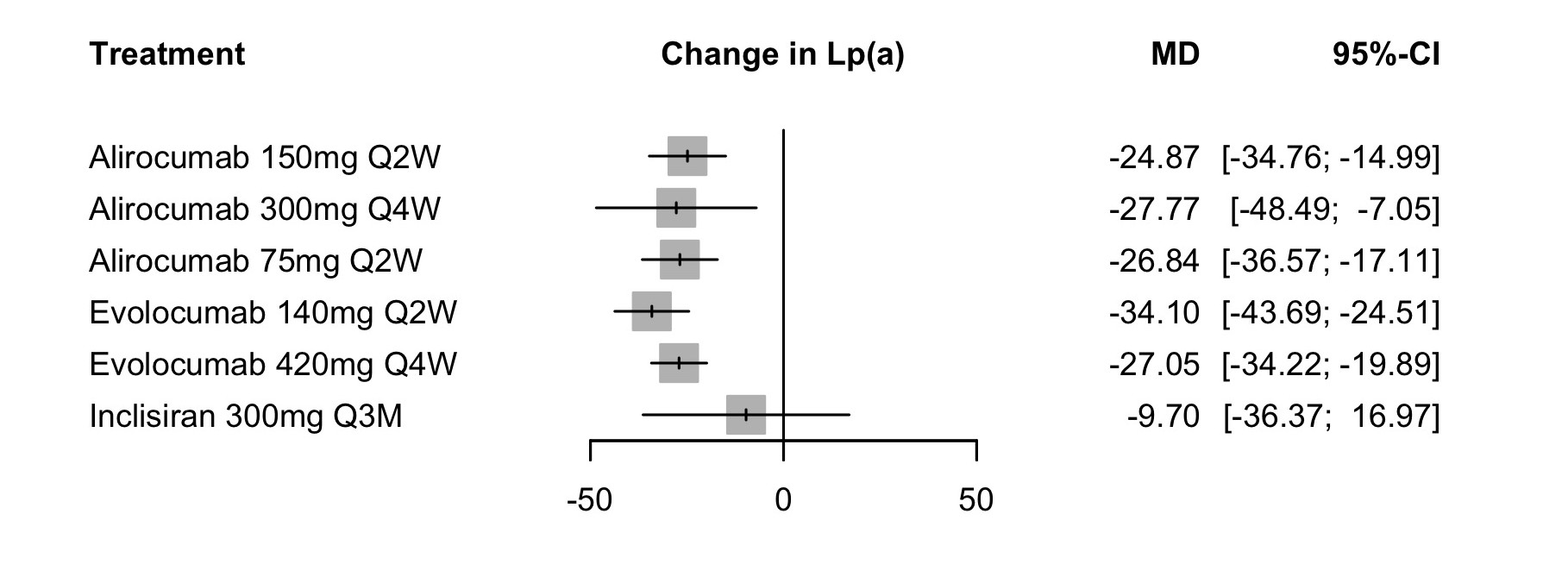
**

**Supplementary Figure S8.6.** Phase 3 funnel plot for the primary Lp(a) outcome.
**
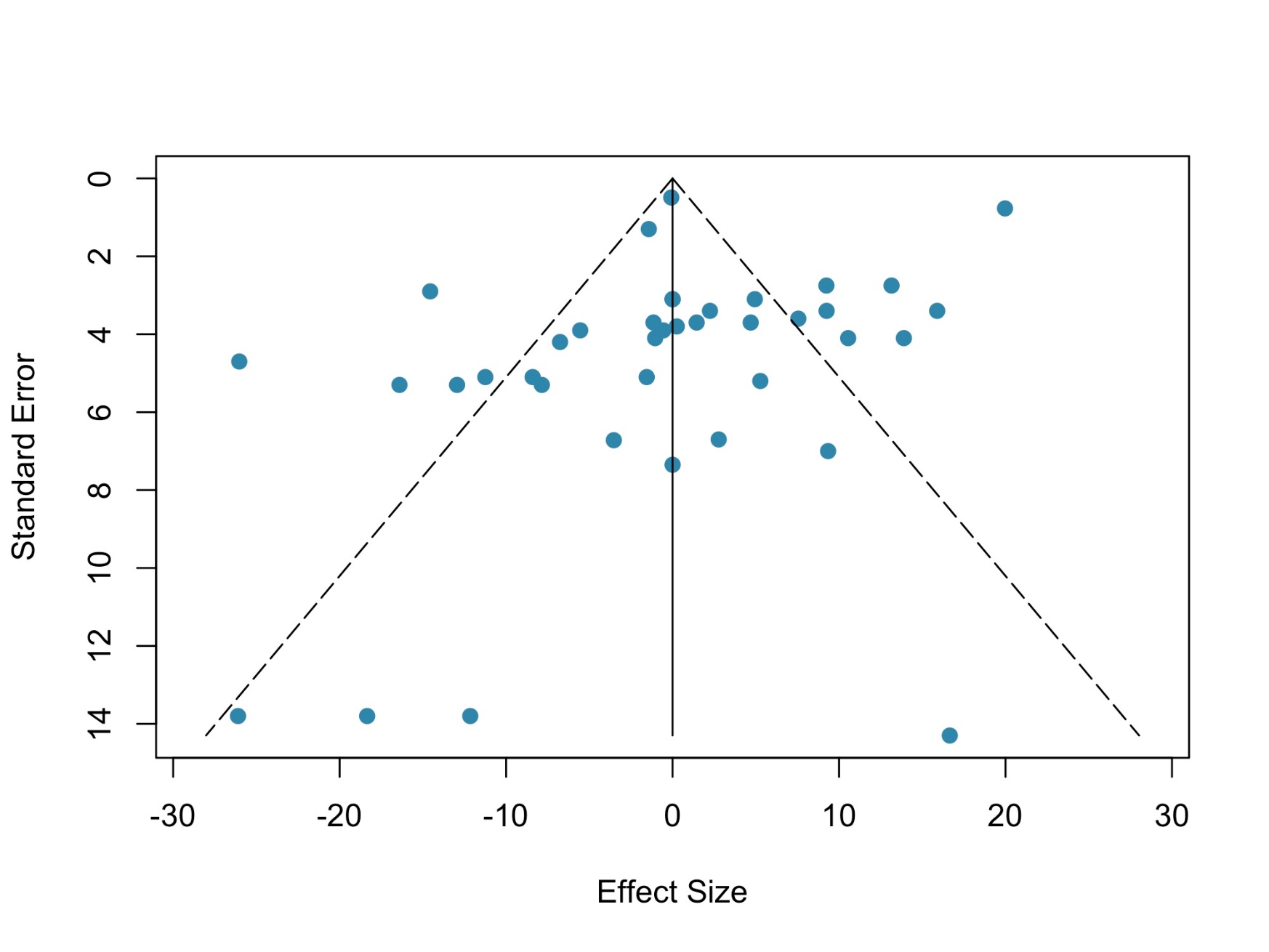
**

### **8.1.2. Univariable meta-regression analyses**

**Supplementary Table S8.1.**Exploratory univariable meta-regression analyses for the primary Lp(a) outcome.
Exploratory univariable meta-regression analyses were performed to assess potential study-level contributors to heterogeneity in the primary Lp(a) network.

| Covariate | Coefficient | SE | p-value |
| --- | --- | --- | --- |
| Age | 0.43 | 0.51 | 0.405 |
| Sample size | 0.011 | 0.0067 | 0.108 |
| Follow-up duration | −0.15 | 0.15 | 0.31 |
| Trial phase | 16.52 | 3.93 | <0.001 |
| Background statin use | 0.21 | 0.11 | 0.069 |
| Background ezetimibe use | −0.052 | 0.15 | 0.735 |
| Background PCSK9 therapy | −2.24 | 0.53 | <0.001 |
| Baseline Lp(a) | −0.13 | 0.04 | 0.003 |
| Treatment class | −19.94 | 3.2 | <0.001 |

### **8.1.3. Population-based subgroup analyses**

**Supplementary Table S8.2.**Population-based subgroup analyses for the primary Lp(a) outcome.
Analyses were exploratory and performed using the random-effects model. Heterogeneity p-values refer to the total Q test. Subgroups with one or two studies were considered too sparse for reliable heterogeneity estimation.

| Population subgroup | Studies, k | Pairwise comparisons, m | Treatments, n | Designs, d | τ² | I² | Heterogeneity p-value |
| --- | --- | --- | --- | --- | --- | --- | --- |
| CAD | 13 | 15 | 10 | 8 | 36.66 | 59.30% | 0.031 |
| DM2 | 3 | 5 | 4 | 3 | 0 | 0% | 0.372 |
| Elevated Lp(a) | 8 | 14 | 10 | 7 | 488.75 | 92.70% | <0.001 |
| Familial hypercholesterolemia | 8 | 12 | 6 | 6 | 0 | 0% | 0.803 |
| Healthy | 2 | 2 | 3 | 2 | NA | NA | NA |
| HIV | 1 | 1 | 2 | 1 | NA | NA | NA |
| Hypercholesterolemia | 14 | 32 | 6 | 7 | 73.62 | 79.50% | <0.001 |

## **8.2. Total cholesterol**

In the random-effects network meta-analysis of 39 randomized controlled trials evaluating 14 active treatments versus placebo (full regimen-specific estimates are shown in Supplementary Figure S8.7 below), PCSK9-directed regimens were associated with the largest reductions versus placebo. The greatest reduction was seen with alirocumab 150 mg Q2W (MD −40.89, 95% CI −48.02 to −33.77), followed by evolocumab 420 mg Q4W (MD −35.28, 95% CI −41.33 to −29.23) and evolocumab 140 mg Q2W (MD −34.21, 95% CI −41.36 to −27.06). Other PCSK9 regimens also reduced total cholesterol, including alirocumab 75 mg Q2W (MD −32.76, 95% CI −40.76 to −24.77), inclisiran 300 mg Q3M (MD −27.96, 95% CI −41.76 to −14.15), and alirocumab 300 mg Q4W (MD −27.66, 95% CI −39.42 to −15.89). In contrast, Lp(a)-targeted therapies were not associated with statistically significant changes in total cholesterol except for pelacarsen 200 mg QW (MD −17.00, 95% CI −39.88 to 5.88). Ranking analyses were consistent with these findings, with alirocumab 150mg Q2W ranking highest (P-score 0.97), followed by evolocumab 420 mg Q4W (P-score 0.84) and evolocumab 140 mg Q2W (P-score 0.81).

Heterogeneity was substantial (I² = 80.6%, 95% CI 74.0–85.5%; τ² = 24.71). Effect estimates and treatment ordering were directionally consistent across sensitivity analyses (excluding high-risk-of-bias trials, restricting to larger studies, common-effects, and leave-one-out), although some between-design inconsistency was observed in the high–risk-of-bias–excluded analysis (Q_between = 26.44, p = 0.0004). Meta-regression did not identify effect modification by age, sample size, or study duration, whereas trial phase was associated with smaller effects in later-phase trials (β = −7.88, SE = 3.13, p = 0.012).

According to the CINeMA framework, the certainty of evidence for total cholesterol was low to very low overall, with most comparisons rated very low, mainly due to heterogeneity, imprecision, and reporting bias (Supplement 11). Funnel-plot asymmetry suggested small-study effects (Supplement 5).


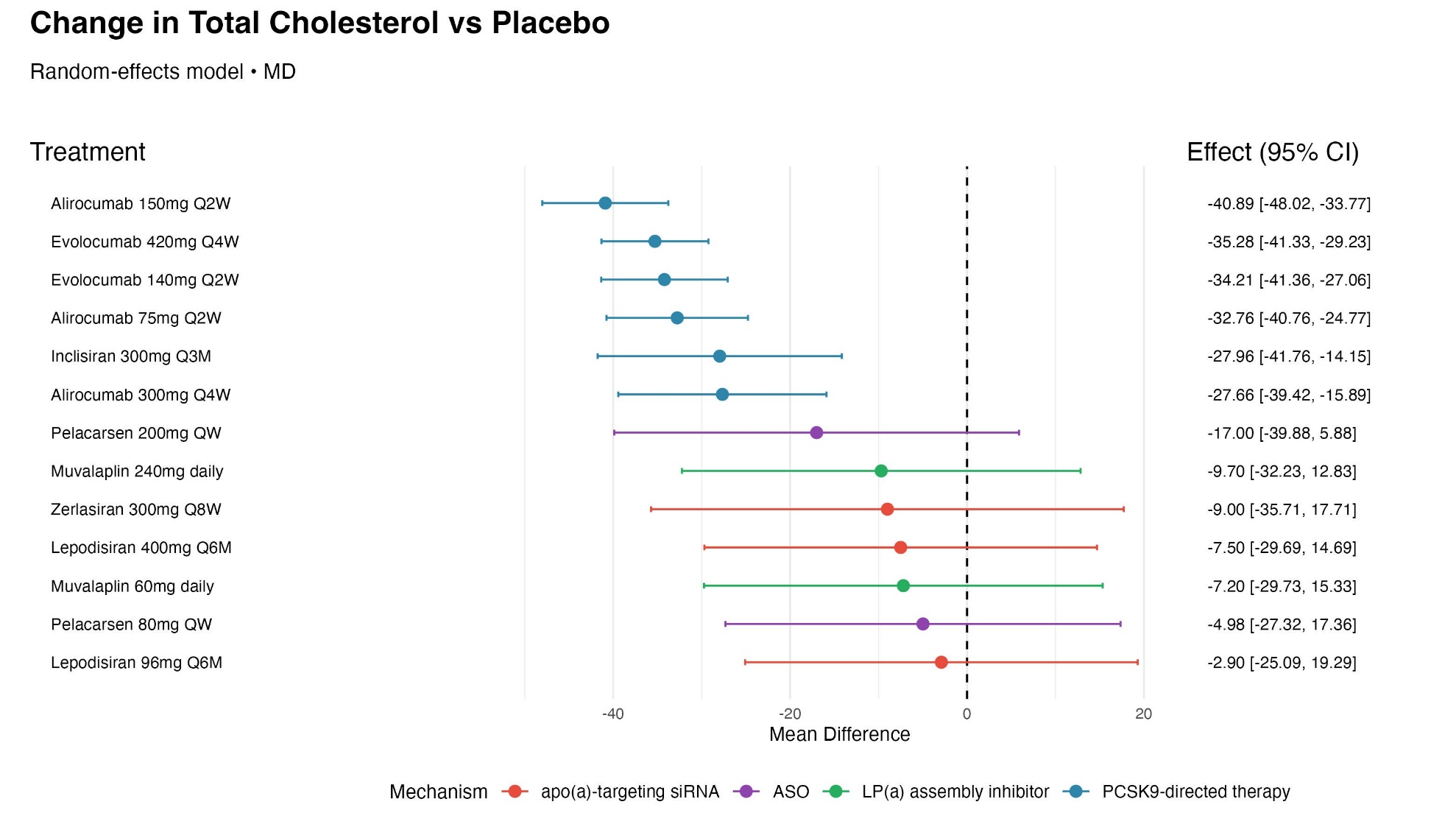


**Supplementary Figure S8.7.** Network meta-analysis estimates for total cholesterol versus placebo. Effect sizes are presented as placebo-adjusted MDs in percent change from baseline (pp) with 95% CIs. More negative values indicate greater reductions in total cholesterol. Abbreviations: QW weekly; Q2W every 2 weeks; Q4W every 4 weeks; Q8W every 8 weeks; Q3M every 3 months; Q6M every 6 months.

## **8.3. HDL-C**

In the random-effects network meta-analysis (full regimen-specific estimates are shown in Supplementary Figure 8.8 below), PCSK9-directed therapies were associated with increases in HDL-C versus placebo, whereas emerging Lp(a) therapies were generally not associated with statistically significant increases, except for muvalaplin 240 mg daily, which was associated with lower HDL-C (MD −6.00, 95% CI −11.50 to −0.50). The largest increases were observed with alirocumab 75 mg Q2W (MD +7.13, 95% CI +5.07 to +9.19), evolocumab 420 mg Q4W (MD +7.12, 95% CI +5.73 to +8.52), and inclisiran 300 mg Q3M (MD +6.88, 95% CI +2.32 to +11.43). Ranking analyses were consistent, with evolocumab 420 mg Q4W ranking highest (P-score 0.82), followed by alirocumab 75 mg Q2W (P-score 0.81) and inclisiran 300 mg Q3M (P-score 0.77).

Between-study heterogeneity was low to moderate (I² = 33.1%, 95% CI 2.5–54.0%; τ² = 2.60). Sensitivity analyses were directionally consistent: after excluding high–risk-of-bias trials (k = 41), PCSK9-directed therapies remained HDL-C–raising versus placebo, lepodisiran and pelacarsen remained largely non-significant, and muvalaplin 240 mg remained HDL-C–lowering. Restriction to larger studies (k = 25) and common-effects/leave-one-out analyses did not materially change conclusions; pelacarsen 80 mg was nominally significant only under fixed-effects assumptions and should be interpreted cautiously. Meta-regression did not identify significant effect modification (all p > 0.05).

According to the CINeMA framework, confidence in HDL-C estimates was generally low to very low, mainly due to heterogeneity, imprecision, and reporting bias (Supplement 11). Funnel-plot asymmetry suggested possible small-study effects (Supplement 5).


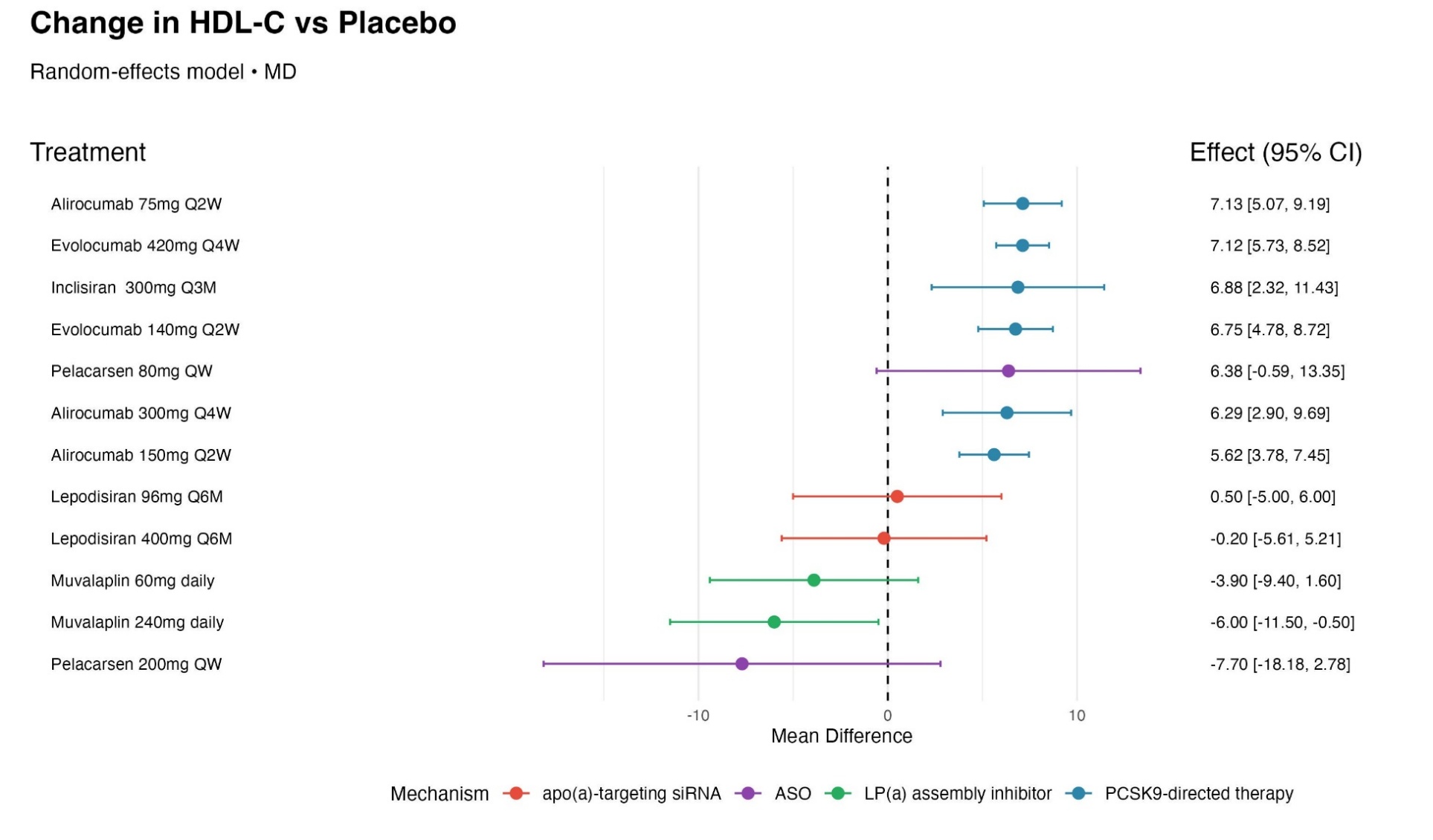


**Supplementary Figure S8.8.** Network meta-analysis estimates for HDL-C versus placebo. Effect sizes are presented as placebo-adjusted MDs in percent change from baseline (pp) with 95% CIs. More positive values indicate greater HDL-C increase. *Abbreviations:* QW weekly; Q2W every 2 weeks; Q4W every 4 weeks; Q8W every 8 weeks; Q3M every 3 months; Q6M every 6 months; QID 4 tablets (given once daily)

**Supplement 9. PRISMA Flow Diagram**


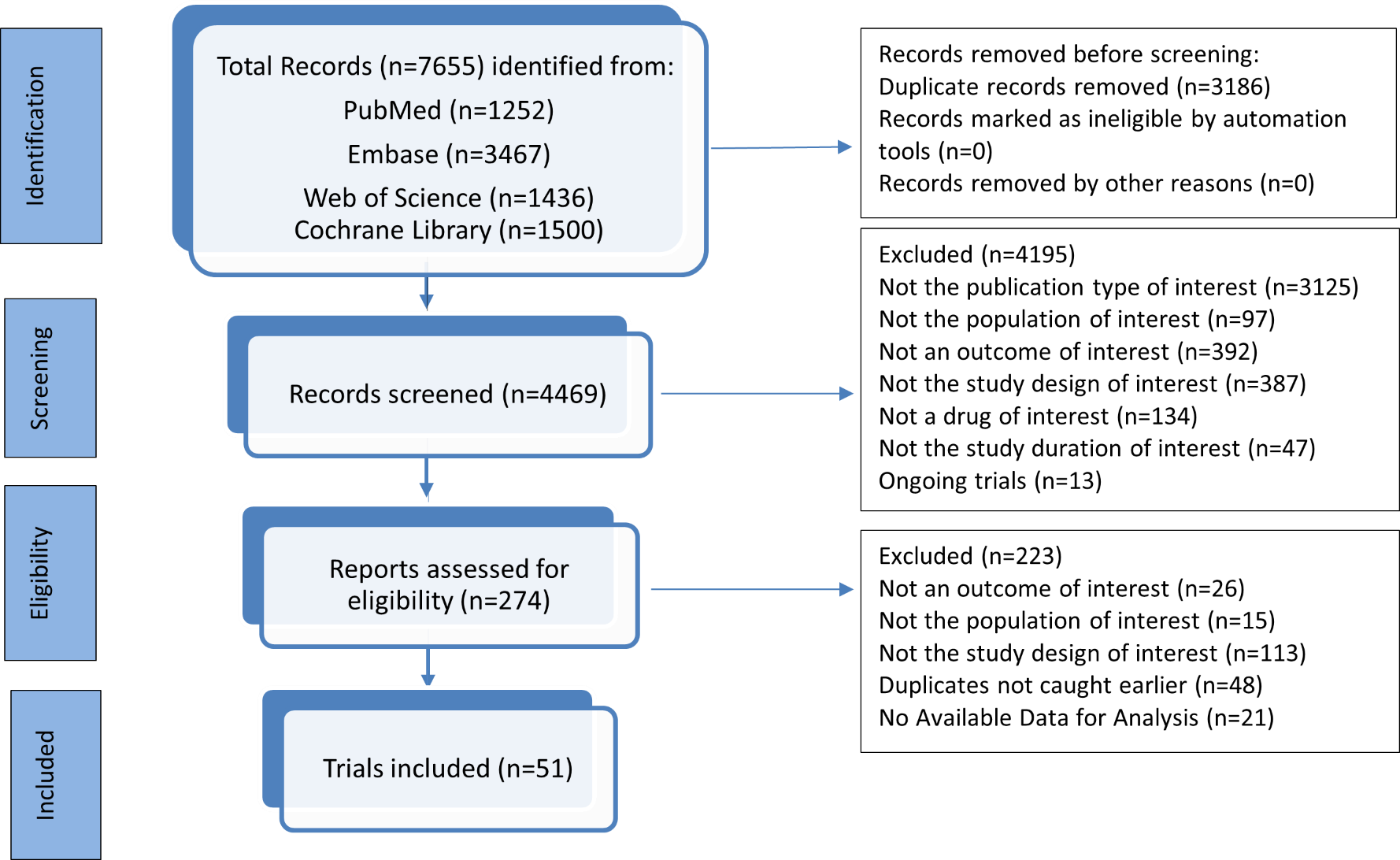


**Supplement 10. PRISMA Checklist**

| **Section and Topic** | **Item #** | **Checklist item** | **Location where item is reported** |
| --- | --- | --- | --- |
| **TITLE** | | |  |
| Title | 1 | Identify the report as a systematic review. | **Page 1**, Title |
| **ABSTRACT** | | |  |
| Abstract | 2 | See the PRISMA 2020 for Abstracts checklist. | **Page 3**, Abstract |
| **INTRODUCTION** | | |  |
| Rationale | 3 | Describe the rationale for the review in the context of existing knowledge. | **Introduction**, Paragraphs 1-3 |
| Objectives | 4 | Provide an explicit statement of the objective(s) or question(s) the review addresses. | **Introduction**, Paragraph 3 |
| **METHODS** | | |  |
| Eligibility criteria | 5 | Specify the inclusion and exclusion criteria for the review and how studies were grouped for the syntheses. | **Methods**, Section 2.2, Paragraph 1 |
| Information sources | 6 | Specify all databases, registers, websites, organisations, reference lists and other sources searched or consulted to identify studies. Specify the date when each source was last searched or consulted. | **Methods**, Section 2.3, Paragraph 1 |
| Search strategy | 7 | Present the full search strategies for all databases, registers and websites, including any filters and limits used. | **Supplement 1** |
| Selection process | 8 | Specify the methods used to decide whether a study met the inclusion criteria of the review, including how many reviewers screened each record and each report retrieved, whether they worked independently, and if applicable, details of automation tools used in the process. | **Methods**, Section 2.3, Paragraph 1 |
| Data collection process | 9 | Specify the methods used to collect data from reports, including how many reviewers collected data from each report, whether they worked independently, any processes for obtaining or confirming data from study investigators, and if applicable, details of automation tools used in the process. | **Methods**, Section 2.4, Paragraph 1 |
| Data items | 10a | List and define all outcomes for which data were sought. Specify whether all results that were compatible with each outcome domain in each study were sought (e.g. for all measures, time points, analyses), and if not, the methods used to decide which results to collect. | **Methods**, Section 2.2, Paragraph 1 |
|  | 10b | List and define all other variables for which data were sought (e.g. participant and intervention characteristics, funding sources). Describe any assumptions made about any missing or unclear information. | **Methods**, Section 2.4, Paragraph 1 |
| Study risk of bias assessment | 11 | Specify the methods used to assess risk of bias in the included studies, including details of the tool(s) used, how many reviewers assessed each study and whether they worked independently, and if applicable, details of automation tools used in the process. | **Methods**, Section 2.4, Paragraph 1,2 |
| Effect measures | 12 | Specify for each outcome the effect measure(s) (e.g. risk ratio, mean difference) used in the synthesis or presentation of results. | **Methods**, Section 2.5, Paragraph 1 |
| Synthesis methods | 13a | Describe the processes used to decide which studies were eligible for each synthesis (e.g. tabulating the study intervention characteristics and comparing against the planned groups for each synthesis (item #5)). | **Methods**, Section 2.5, Paragraph 2 |
|  | 13b | Describe any methods required to prepare the data for presentation or synthesis, such as handling of missing summary statistics, or data conversions. | **Methods**, Section 2.4, Paragraph 2 |
|  | 13c | Describe any methods used to tabulate or visually display results of individual studies and syntheses. | **Methods**, Section 3.1, Paragraph 1 |
|  | 13d | Describe any methods used to synthesize results and provide a rationale for the choice(s). If meta-analysis was performed, describe the model(s), method(s) to identify the presence and extent of statistical heterogeneity, and software package(s) used. | **Methods**, Section 2.5, Paragraph 1 |
|  | 13e | Describe any methods used to explore possible causes of heterogeneity among study results (e.g. subgroup analysis, meta-regression). | **Methods**, Section 2.6, Paragraph 1 |
|  | 13f | Describe any sensitivity analyses conducted to assess robustness of the synthesized results. | **Methods**, Section 2.6, Paragraph 1 |
| Reporting bias assessment | 14 | Describe any methods used to assess risk of bias due to missing results in a synthesis (arising from reporting biases). | **Methods**, Section 2.6, Paragraph 1 |
| Certainty assessment | 15 | Describe any methods used to assess certainty (or confidence) in the body of evidence for an outcome. | **Methods**, Section 2.4, Paragraph 2 |
| **RESULTS** | | |  |
| Study selection | 16a | Describe the results of the search and selection process, from the number of records identified in the search to the number of studies included in the review, ideally using a flow diagram. | **Methods**, Section 2.3 + Fig. 1+  **Results**, Section 3.1 |
|  | 16b | Cite studies that might appear to meet the inclusion criteria, but which were excluded, and explain why they were excluded. | **Methods**, Section 2.2 |
| Study characteristics | 17 | Cite each included study and present its characteristics. | **Supplement 3** |
| Risk of bias in studies | 18 | Present assessments of risk of bias for each included study. | **Supplement 4** |
| Results of individual studies | 19 | For all outcomes, present, for each study: (a) summary statistics for each group (where appropriate) and (b) an effect estimate and its precision (e.g. confidence/credible interval), ideally using structured tables or plots. | **Supplement 6** |
| Results of syntheses | 20a | For each synthesis, briefly summarise the characteristics and risk of bias among contributing studies. | **Results**, Section 3.2, Paragraph 1 |
|  | 20b | Present results of all statistical syntheses conducted. If meta-analysis was done, present for each the summary estimate and its precision (e.g. confidence/credible interval) and measures of statistical heterogeneity. If comparing groups, describe the direction of the effect. | **Results**, Section 3.2, Paragraph 1 |
|  | 20c | Present results of all investigations of possible causes of heterogeneity among study results. | **Results**, Section 3.2, Paragraph 3 |
|  | 20d | Present results of all sensitivity analyses conducted to assess the robustness of the synthesized results. | **Results**, Section 3.2, Paragraph 2 |
| Reporting biases | 21 | Present assessments of risk of bias due to missing results (arising from reporting biases) for each synthesis assessed. | **Supplement 5** |
| Certainty of evidence | 22 | Present assessments of certainty (or confidence) in the body of evidence for each outcome assessed. | **Extra Supplementary file- CINEMA** |
| **DISCUSSION** | | |  |
| Discussion | 23a | Provide a general interpretation of the results in the context of other evidence. | **Discussion**, Paragraph 1 |
|  | 23b | Discuss any limitations of the evidence included in the review. | **Discussion**, Paragraph 1 |
|  | 23c | Discuss any limitations of the review processes used. | **Discussion**, Paragraph 1 |
|  | 23d | Discuss implications of the results for practice, policy, and future research. | **Discussion,** Paragraphs 2-3 |
| **OTHER INFORMATION** | | |  |
| Registration and protocol | 24a | Provide registration information for the review, including register name and registration number, or state that the review was not registered. | **Methods**, Section 2.1, Paragraph 1 |
|  | 24b | Indicate where the review protocol can be accessed, or state that a protocol was not prepared. | **Methods**, Section 2.1, Paragraph 1 |
|  | 24c | Describe and explain any amendments to information provided at registration or in the protocol. |  |
| Support | 25 | Describe sources of financial or non-financial support for the review, and the role of the funders or sponsors in the review. | **Page 17**, Section 5 (Acknowledgments) |
| Competing interests | 26 | Declare any competing interests of review authors. | **Submitted as a separate file.** |
| Availability of data, code and other materials | 27 | Report which of the following are publicly available and where they can be found: template data collection forms; data extracted from included studies; data used for all analyses; analytic code; any other materials used in the review. | **Results**, Section 2.1 |
